# Meiosis Drives Extraordinary Genome Plasticity in the Haploid Fungal Plant Pathogen *Mycosphaerella graminicola*

**DOI:** 10.1371/journal.pone.0005863

**Published:** 2009-06-10

**Authors:** Alexander H. J. Wittenberg, Theo A. J. van der Lee, Sarrah Ben M'Barek, Sarah B. Ware, Stephen B. Goodwin, Andrzej Kilian, Richard G. F. Visser, Gert H. J. Kema, Henk J. Schouten

**Affiliations:** 1 Plant Research International B.V., Wageningen University and Research Centre, Wageningen, The Netherlands; 2 United States Department of Agriculture (USDA)-Agricultural Research Service (ARS), Crop Production and Pest Control Research Unit, and Department of Botany and Plant Pathology, Purdue University, West Lafayette, Indiana, United States of America; 3 Diversity Arrays P/L, Yarralumla, Canberra, Australian Capital Territory, Australia; 4 Graduate School Experimental Plant Sciences, Laboratory of Plant Breeding, Department of Plant Sciences, Wageningen University and Research Centre, Wageningen, The Netherlands; 5 Graduate School Experimental Plant Sciences, Laboratory of Phytopathology, Department of Plant Sciences, Wageningen University and Research Centre, Wageningen, The Netherlands; University of California, Berkeley, United States of America

## Abstract

Meiosis in the haploid plant-pathogenic fungus *Mycosphaerella graminicola* results in eight ascospores due to a mitotic division following the two meiotic divisions. The transient diploid phase allows for recombination among homologous chromosomes. However, some chromosomes of *M. graminicola* lack homologs and do not pair during meiosis. Because these chromosomes are not present universally in the genome of the organism they can be considered to be dispensable. To analyze the meiotic transmission of unequal chromosome numbers, two segregating populations were generated by crossing genetically unrelated parent isolates originating from Algeria and The Netherlands that had pathogenicity towards durum or bread wheat, respectively. Detailed genetic analyses of these progenies using high-density mapping (1793 DArT, 258 AFLP and 25 SSR markers) and graphical genotyping revealed that *M. graminicola* has up to eight dispensable chromosomes, the highest number reported in filamentous fungi. These chromosomes vary from 0.39 to 0.77 Mb in size, and represent up to 38% of the chromosomal complement. Chromosome numbers among progeny isolates varied widely, with some progeny missing up to three chromosomes, while other strains were disomic for one or more chromosomes. Between 15–20% of the progeny isolates lacked one or more chromosomes that were present in both parents. The two high-density maps showed no recombination of dispensable chromosomes and hence, their meiotic processing may require distributive disjunction, a phenomenon that is rarely observed in fungi. The maps also enabled the identification of individual twin isolates from a single ascus that shared the same missing or doubled chromosomes indicating that the chromosomal polymorphisms were mitotically stable and originated from nondisjunction during the second division and, less frequently, during the first division of fungal meiosis. High genome plasticity could be among the strategies enabling this versatile pathogen to quickly overcome adverse biotic and abiotic conditions in wheat fields.

## Introduction

Fungi provide attractive model systems to analyze processes that occur during meiosis. Many fungi are haploid, which greatly simplifies genetic studies. Furthermore, complete recovery of the meiotic products, or tetrads, is possible in ascomycete fungi, and these tetrads can be analyzed for the segregation of genetic markers. Tetrad analyses of *Aspergillus nidulans* and *Neurospora crassa* have been instrumental in answering fundamental questions concerning meiosis [1]–[3]. Here we describe genetic studies in another filamentous ascomycete, *Mycosphaerella graminicola* (asexual stage: *Septoria tritici*). This fungus causes septoria tritici blotch (STB) of wheat, a disease characterized by necrotic blotches on the foliage. These blotches contain asexual (pycnidia) and sexual (pseudothecia) fructifications. *M. graminicola* represents an intriguing model for fundamental genetic studies of plant-pathogenic fungi. Field isolates of this pathogen usually have 18–21 chromosomes, the highest number reported among ascomycetes. Furthermore, these chromosomes have an extraordinary size range, varying from 0.39 to 6.09 Mb [4]. Genome plasticity - comprising processes such as inversions, deletions, insertions and translocations that translate into chromosome length polymorphisms (CLPs) as well as chromosome number polymorphisms (CNPs) - results in a genome size that varies between 32 and 40 Mb, similar to other filamentous ascomycetes [4]–[9]. *M. graminicola* has an active sexual cycle under natural conditions, which is an important driver of STB epidemics and results in high genetic diversity of populations in the field [10]–[12].

Analyses of a cross between two *M. graminicola* strains that originated from bread wheat fields in The Netherlands resulted in the first genetic linkage map of a *Mycosphaerella* species [13], [14]. Although this map was a major milestone, the anonymous AFLP and RAPD markers complicated integration of genetic data sets. In addition, the number of markers was limited and the map resolution was too low to assess the complications anticipated during meiosis due to the CLPs and CNPs commonly observed among *M. graminicola* isolates [9].

The exact origin and maintenance of CNPs and CLPs are not known. A likely hypothesis is that they can be generated or lost during meiosis. Recombination between chromosomes that differ in length could give rise to derivatives with CLPs [15]. Nondisjunction during meiosis I or II would generate CNPs. To test these hypotheses, we used the recently developed Diversity Arrays Technology (DArT) for the first time on a haploid fungal genome [16]–[20]. The parallel genotyping of progeny isolates using several thousands of DNA fragments spotted on a microarray and subsequent analysis resulted in one of the most dense genetic linkage maps currently available for a fungus. This enabled high-resolution genetic linkage analyses to study the meiotic processing of CNPs and CLPs as well as the generation of new genome plasticity in *M. graminicola*. We frequently observed the loss of one or more chromosomes, disomy and translocations. This extraordinary genome plasticity helps to explain the high genetic diversity observed within natural populations of this fungus and most likely facilitates rapid adaptation to changing environments.

## Materials and Methods

### Fungal isolates and DNA extraction

We used three isolates of *M. graminicola*: IPO323 and IPO94269 were isolated from bread wheat in the Netherlands and IPO95052 was isolated from durum wheat in Algeria. Isolate IPO323 was crossed to both IPO94269 and IPO95052 using a previously developed *in planta* protocol [10], resulting in 68 and 148 progeny, respectively. All progeny isolates were collected and analyzed individually. DNA of parents and progeny was isolated using the Wizard Genomic DNA purification kit (Promega Madison, WI), starting with approximately 10 mg of lyophilized spores. Tables S1 and S2 provide an overview of the progeny isolates used in this study.

### DArT procedure

Generation of genomic representations, library construction, target preparation and image analysis were essentially performed as described previously [17], [18], with the modifications described by Wittenberg et al. [16]. The adapter and primer oligonucleotide sequences used in this study are listed in Table S3. For details see Text S1.

### Nomenclature of markers

AFLP markers were designated by the primer combination used for the amplification and the approximate length of the generated fragment [14]. For both AFLP and DArT markers the prefix A or B indicated the phase of the marker; those originating from parent IPO323 had the prefix A while markers from parent IPO95052 were indicated by the prefix B. DArT markers identified in cross IPO323×IPO94269 originating from isolate IPO95052 could be assigned the prefix A or B, as IPO94269 was not used for the library construction. Markers segregating in both populations received the prefix C. In addition, DArT markers were designated by the enzyme combination used for complexity reduction (BamHI, MseI and RsaI: BMR or HindIII, MseI, RsaI: HMR), the 384-well plate number and the position of the fragment in that plate (i.e., AHMR_04I09). Recently, 23 SSR loci were identified in *M. graminicola*, 21 of which could be positioned on the existing linkage map along with two previously published SSR loci [21], [22]. The newly generated DArT markers were used to integrate the new IPO323×IPO94269 map with the existing map of that population [14]. Moreover, six of these SSRs also differentiated the parents of the second mapping population. To enable the mapping of these SSRs in the IPO323×IPO95052 progeny, amplification reactions were performed as described by Goodwin et al. [21].

### Selection of unique segregation patterns and merging of twin isolates

The binary scores of polymorphic markers were converted to the correct allelic phase based on the scores of the parents. A Perl script was written that grouped loci with identical segregation patterns after disregarding unknown scores. The marker with the highest call rate (percentage of scored individuals) was selected as a representative for each group. The script also calculated the call rate for each individual genotype and the global call rate for the whole dataset. Individual genotypes were incorporated into the scoring table when at least 95% of the grouped markers could be scored. In *M. graminicola*, twin progeny isolates arise from the mitotic division that follows meiosis II in the ascus, resulting in four pairs of genetically identical ascospores. Although the random-ascospore progenies that resulted from the crossing protocol minimized the isolation of twin isolates, the large number of markers identified identical progeny efficiently. These were used to calculate the reproducibility of the different marker types and were merged before the mapping analyses.

### Construction and comparison of the linkage maps

The genetic linkage maps of the individual crosses as well as the bridge map were constructed with the software package JoinMap 3.0 [23]. A detailed description of the mapping process for the individual maps is given in Text S1. The use of IPO323 in both crosses enabled the efficient generation of an integrated bridge map of the *M. graminicola* genome. The bridge map was used to compare the order of the loci in the constructed IPO323×IPO94269 and IPO323×IPO95052 maps. We used MapChart 2.2 [24] for the graphical representation of the genetic linkage maps.

### Evaluation of loss or gain of chromosomes

We used graphical genotyping to compare the marker scores (A or B) and the phase (A or B) of the markers, which enabled us to identify whether each marker was present or absent in a particular progeny isolate. In cases where a linkage group (LG) was constructed from both marker types and a specific progeny isolate lacked all of these markers, we concluded that the isolate missed that LG. In cases where a LG was constructed from both marker types and a specific progeny isolate was scored present for all markers, we concluded that the isolate had an extra copy, derived from the other parent, of that particular LG. Hence, chromosome polymorphisms in progeny isolates were determined *in silico* if A and B markers that were assigned to a specific LG were always absent or present in a particular progeny.

### PCR verification of loss and gain of chromosomes

DNA samples of the parental isolates (IPO323, IPO94269 and IPO95052), progeny isolates that showed absence of specific LGs by graphical genotyping (Table S4) and two control progeny without these aberrations were used as templates in the PCR reactions. PCR was performed using SSR markers and specific primer pairs developed from the sequenced DArT markers located on the missing linkage groups (Tables S5 and S6). To assure that absence of an amplicon was not caused by PCR failure, a positive PCR control was included that should be present in all parents and progeny that were tested. The SSR marker loci *ac-0007* (LG8) and *gga-0001* (LG12) were amplified in combination with the PCR control SSR locus *ag-0003* (LG2). For the amplicons derived from the DArT marker sequences, the DArT fragments CABMR_07D07 (129 bp; LG1) or AHMR_08O09 (728 bp; LG15) served as positive PCR controls.

PCR reactions were performed in a total volume of 20 µl containing 20 ng of genomic DNA, 1×PCR buffer (Roche), 1 µl of each of the forward and reverse primers used as a control (2 µM), 2 µl of each forward and reverse primer (2 µM), 0.8 µl of dNTPs (5 mM) and 0.2 µl of Taq DNA polymerase (5 U/µl). Amplification conditions were as follows: 94°C for 2 min, 12 cycles of 94°C for 30 sec, 66°C for 30 sec minus 1°C per cycle, 72°C for 30 sec; 27 cycles of 94°C for 30 sec, 53°C for 30 sec, 72°C for 30 sec; 72°C for 7 min, followed by a cooling-down step to 10°C. The SSR amplicons were separated on 6% non-denaturating acrylamide gels using a Mega-Gel Dual High-Throughput Vertical Electrophoresis Unit (CBS Scientific, Del Mar, California, USA). Amplicons based on the DArT sequences were separated on 2.5% agarose gels.

## Results

### Marker selection and quality

Among the 68 progeny isolates from the *M. graminicola* IPO323×IPO94269 cross, 1042 new DArT markers were obtained. The DArT markers were added to the first genetic linkage map of *M. graminicola* [14], consisting of 271 AFLP markers, 57 RAPD markers and two markers for the biological traits avirulence (*Avr*) and mating type (*mat*). Twenty-five SSR markers also were added to the combined linkage map (Table S7) [21]. For the 148 progeny isolates of the *M. graminicola* IPO323×IPO95052 cross, 1154 DArT markers were obtained that were combined with six SSR markers and the markers for the two biological traits (Table S8). After analysis of the marker data, 31 twins were detected in the *M. graminicola* progenies (Table S9). These twins result from the mitotic division that follows meiosis II in the ascus. The twin data enabled the dissection of mitotic or meiotic events that drive the generation of CLPs and CNPs. Eventually, the merged scoring tables comprised 60 individuals for the IPO323×IPO94269 cross and 125 individuals for the IPO323×IPO95052 cross (Table S10). Because twins can be regarded as biological replicates, they also were used to evaluate the reproducibility of the marker scores for the different marker technologies. In our study, DArT and AFLP markers appeared to be more reproducible than the RAPD markers. Therefore, RAPD markers were excluded to improve the quality of the maps. Although the reproducibility for both DArT and AFLP was very high, the frequency of double crossovers in the final maps was much lower for DArT than for AFLP markers (0.24% compared to 0.96%), indicating the superior reliability of the DArT markers.

### Construction and comparison of the linkage maps

The combined genetic linkage maps contain 2078 markers comprising 1793 DArT, 258 AFLP, and 25 SSR DNA markers, plus the two markers that co-segregate with the biological traits *Mat* and *Avr* (Table S11). The grouping and the order of the markers in the *M. graminicola* IPO323×IPO94269 cross were highly similar to those in the previous maps [14], [21]. Compared to the previous map both new maps span a considerably larger part of the genome. In both crosses close to 99% of the segregating markers were reliably positioned, indicating that the current genetic linkage maps cover the complete genome.

The new genetic linkage map of the IPO323×IPO94269 cross is 638 cM longer than the first linkage map, and spans 1854 cM with 1317 markers on 451 unique map positions, with an average distance of 4.1 cM between the markers (Table S12). Nearly all markers (98.2%) were positioned on 24 LGs. Some of the smaller LGs that were observed in the first map merged with other LGs [14]: 10 LGs in the first map merged into five larger LGs, while six small new LGs were formed. For example, LGs 3 and 4 in the first map merged with LGs 22 and 17, respectively, in the new map. The order of the AFLP markers in the first and new map remained similar, although more AFLP markers were positioned in the latter (223 vs. 258 out of 271, representing 82.3% and 95.2%, respectively). The genetic linkage map of the *M. graminicola* isolate IPO323×IPO95052 cross spans 1946 cM and contains 1144 markers on 486 unique map positions on 23 LGs (comprising 98.5% of the generated markers), with an average distance of 4.0 cM between the markers (Table S12).

**Figure 1:**
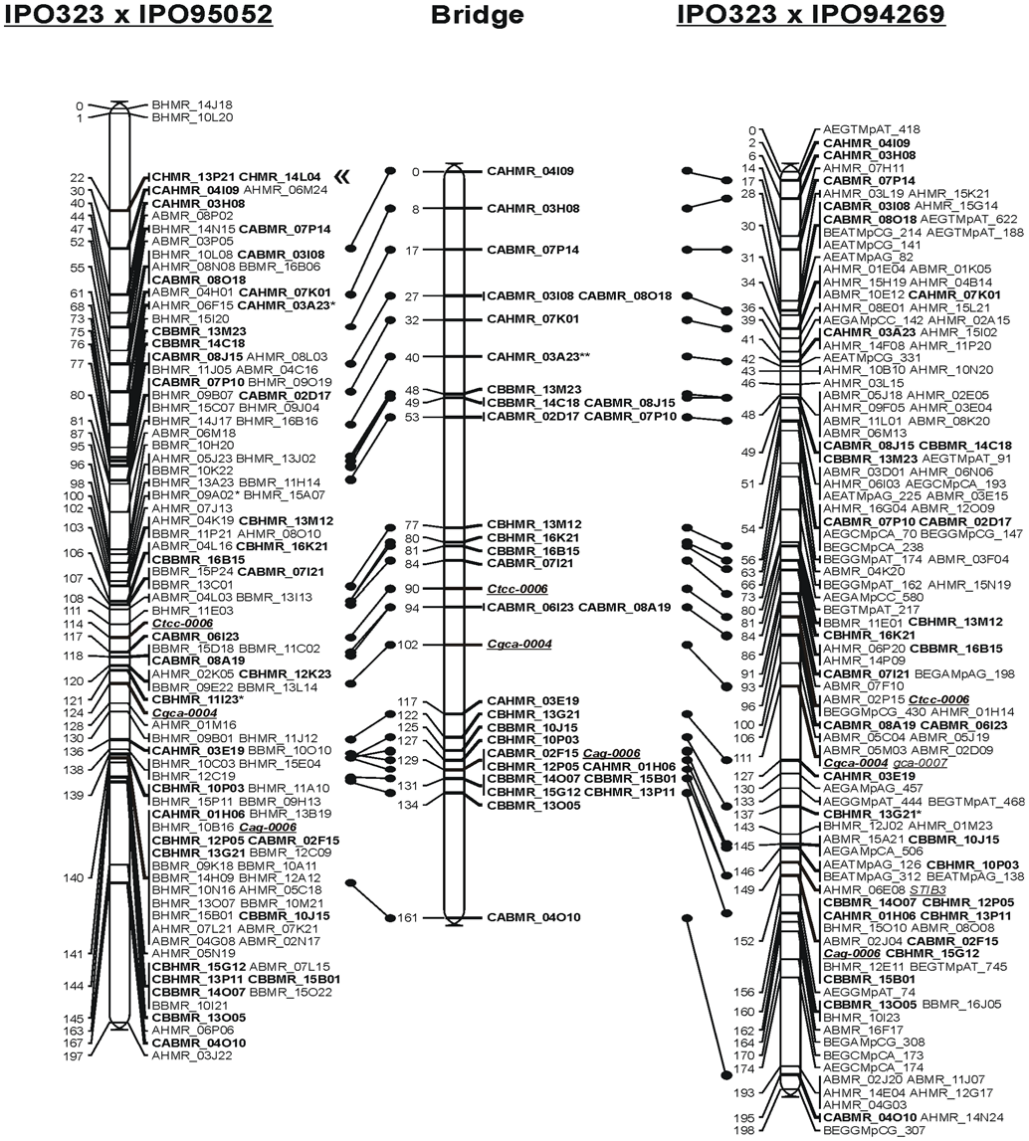
Co-linearity of genetic linkage maps for Mycosphaerella graminicola crosses IPO323×IPO95052 (left) and IPO323×IPO94269 (right) with a bridge map (middle) generated with markers that segregated in both crosses. Common markers are shown in bold and start with the prefix C, SSR markers are shown in blue and markers that are translocated in red. DArT markers were named according to phase of the marker (A = IPO323, B = IPO95052 or IPO94269), complexity reduction method used (BMR or HMR), and location in the spotting plate (e.g. BBMR_15L11). LG and AFLP nomenclature is according to Kema et al., 2002. Segregation distortion of the markers is indicated with * (P<0.05), ** (P<0.01), *** (P<0.005) or **** (P<0.001).

We also constructed a bridge map to compare the individual linkage maps using markers that segregated in both mapping populations. The resulting integrated map spans 1435 cM (∼75% of both individual maps) and contains 372 markers on 251 unique map positions. A total of 22 LGs from each of the individual crosses was aligned with the bridge map, and the marker order was similar to those on the two individual genetic maps (Figure 1 and Figure S1). The 21 LGs in the bridge map is close to the estimated number of chromosomes based on electrophoretic and cytological karyotyping [4], [14] and is identical to the number of chromosomes of the finished genome sequence (http://genome.jgi-psf.org/Mycgr3/Mycgr3.home.html) (Table S13).

### Translocations

We identified eight DArT markers that were positioned very differently in the two maps, which is indicative of translocations. They represented five translocations between isolate IPO323 and either IPO94269 or IPO95052 and involved four inter-LG and one intra-LG translocations (CBBMR_14G17 in LG 6) (Table S14). Another translocation between IPO323 and IPO94269 involved an SSR locus [21] that segregated in a diploid fashion in the isolate IPO323×IPO94269 cross (1∶1∶1∶1 ratio, χ^2^ = 1.25, 0.25<P<0.75) and was mapped on LG 21 in IPO323 and on LG 4+17 in IPO94269. In addition, we obtained indications for a possible larger translocation involving LG F (Figure S1).

### Meiosis drives extraordinary genome plasticity

#### Parental CNPs

LGs 21 and C in the *M. graminicola* IPO323×IPO94269 cross span less than 2 cM and contain 21 and 36 markers (AFLP, SSR and DArT), respectively. Interestingly, all of these markers are inherited from isolate IPO323. This suggests that these two LGs are present in IPO323 but absent in isolate IPO94269. In the progeny of the IPO323×IPO95052 cross these linkage groups do show recombination, which resulted in much larger genetic distances of 21 cM and 24 cM, respectively. These results indicate that both linkage groups are present in isolates IPO323 and IPO95052, but are absent in IPO94269. An example of the difference in recombination frequency is shown for LG 21 in Figure S2.

#### Meiotic transmission of CNPs

**Figure 2:**
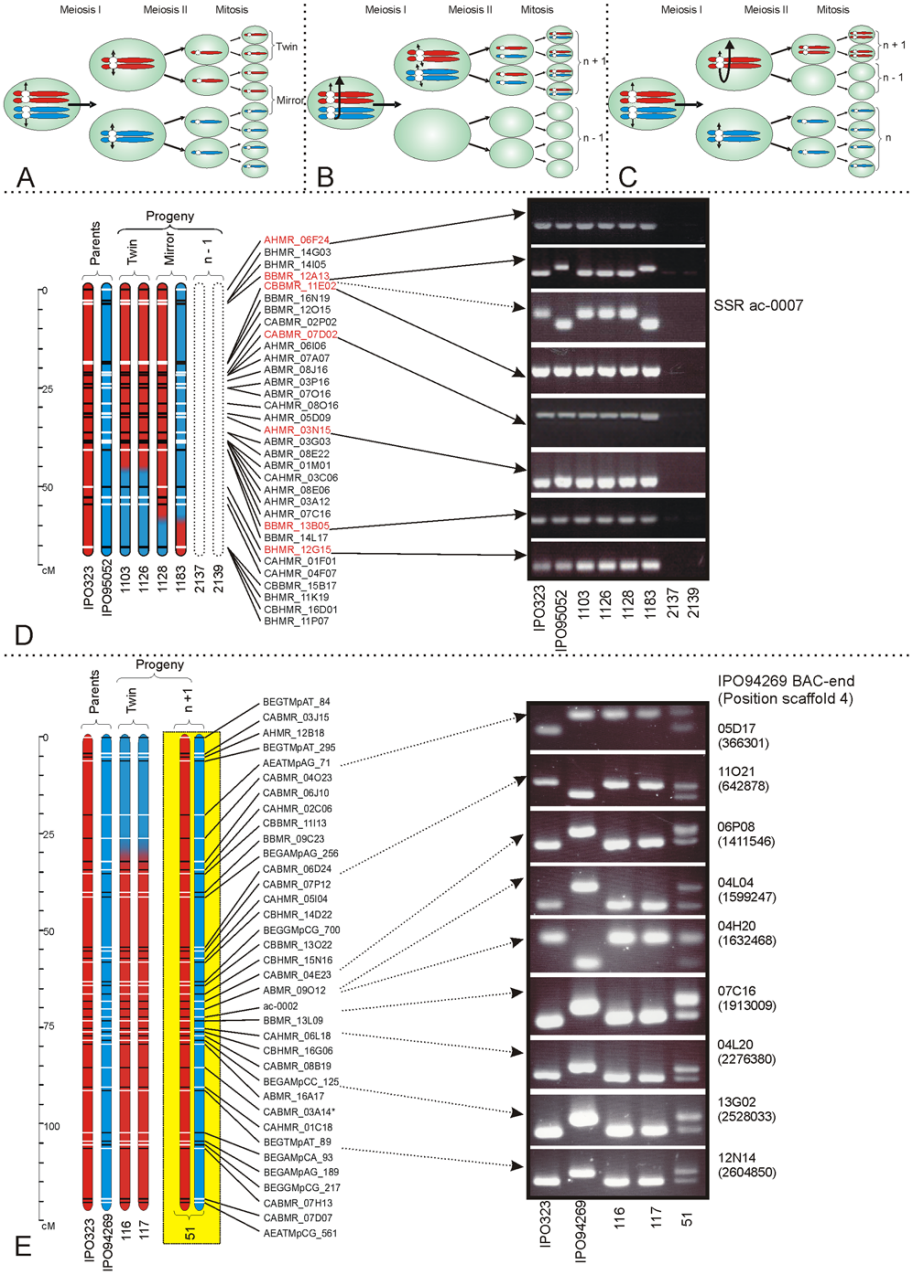
Nondisjunction during meiosis in the haploid fungus Mycosphaerella graminicola results in chromosome number polymorphisms due to the loss or gain of specific chromosomes. A. Meiosis starts with the merging of nuclei from two different strains, leading to a transitory diploid cell. Karyogamy is followed immediately by meiosis I and II, resulting in four haploid cells. These four cells are duplicated during a subsequent mitotic step, leading to eight ascospores per ascus. Each ascospore is genetically identical to one other ascospore within the same ascus. Such pairs of identical ascospores are called twins. We identified several twins in progenies of M. graminicola. When a strain of a descendant lacked one or more chromosomes, the twins originating from the first mitotic cell division after meiosis always appeared to lack the same chromosomes. This indicates that chromosomes are stable during mitosis but can be lost during meiosis. B. Chromosome loss during meiosis can be a result of failure of separation of homologous chromosomes during meiosis I, or C. of the failure of separation of sister chromatids during meiosis II. D. Graphical genotyping of LG 8. The chromosomal segments descending from IPO323 are rendered in red, and the segments from IPO95052 in blue. Markers are scored as present (black) or absent (white). As the marker scores on all linkage groups were identical for these two isolates, we concluded that the descendants 2137 and 2139 are twins. However, both isolates lack all markers located on LG 8. This is a clear indication of absence of this linkage group in these isolates. Strikingly, this linkage group is present in both parents. For further verification, seven DArT markers spanning the length of LG 8 were converted into simple PCR markers. In addition, one SSR marker was used. All markers appeared to be absent in the twin isolates 2137 and 2139. This confirms the absence of LG 8 in these twins and indicates nondisjunction during meiosis as the cause. E. Nondisjunction not only results in loss of a chromosome in one twin but also to disomy for that chromosome in another twin from the same ascus. The graphical genotyping of isolate #51 illustrates heterozygous disomy for LG 1, which was confirmed by a PCR screen for deletion markers that unequivocally showed the presence of two copies of this chromosome in this haploid fungus.

Graphical genotyping allows the tracing of the genetic make up of progeny isolates. Among the progeny of the *M. graminicola* IPO323×IPO95052 cross, LGs that were regularly absent either individually or in combination included LGs 8, 12, 13, 15, 21, A, B and C. LGs 21 and C are absent in IPO94269, and frequently were missing in the *M. graminicola* IPO323×IPO94269 progeny along with LGs 8, 12, 13 and A that were also often missing in this progeny (Table S4). In these cases LGs present in both parents were absent in one or more progeny isolates (Figure 2). We also observed a progeny isolate (#40) from the *M. graminicola* IPO323×IPO94269 cross that contained all markers from both parents on LG 13, indicating that this isolate was disomic for this relatively small chromosome (577 kb). In the same progeny set we identified another isolate (#51) that was disomic for LG 1, which represents one of the largest chromosomes (3.26 Mb) in the genome of *M. graminicola* isolate IPO323. If nondisjunction occurs during meiosis I, two paired chromosomes are pulled to one cell leading to loss of that chromosome in the other cell (Figure 2B). In this case, one haploid *M. graminicola* isolate would become heterozygous disomic for that chromosome. If nondisjunction occurs during meiosis II, two sister chromatids are not divided between the two cells but are both pulled to the same cell (Figure 2C). This evidently leads to two identical copies of the chromosome in that cell, and hence to homozygous disomy in one cell and to absence in the other cell. Unfortunately, homozygous disomy could not be detected with the techniques used for our analysis.

#### Twins do not show CNPs

The large number of markers permitted easy identification of identical progeny and allowed determination of the stage at which CNPs were generated. In total, we detected 31 twins in the *M. graminicola* progenies, whose identity was visualized by graphical genotyping (Table S12). In four cases we could demonstrate that LGs that were present in both parents were absent in both isolates of a twin pair (Table S15). This is illustrated for twin pair 2137–2139 in Figure 2D.

#### PCR confirmation

The observed aberrations and graphical genotyping analyses were confirmed by PCR assays (Figure 2D, E). Additional SSR and PCR assays confirmed the graphical genotyping results for six out of eight LGs. The absence of two LGs was confirmed by scoring of co-dominant SSR markers that are located on LG 8 (*ac-0007*) and LG 12 (*gga-0001*). In all progeny isolates that lacked these LGs, none of the parental alleles was amplified (Figure S3A). The absence of LGs 8, 12, 13, 15, A and C was further confirmed by diagnostic PCR analysis (Table S5) for the mapped DArT markers. Indeed, none of these markers was amplified in the progeny isolates that, according to the graphical genotyping, lacked these particular LGs (Figure S3B and S3C). However, amplicons of the expected size were always generated from the relevant checks, i.e., parental isolates, two progeny isolates that inherited the LG normally and a PCR amplification control.

To confirm the disomy for LG 1 in progeny isolate #51, we performed PCR assays based on deletion polymorphisms (Figure 2E, Table S6) identified by comparative analyses of IPO94269 BAC-end sequences with the draft genome sequence (v.2.5) of IPO323. These PCRs confirmed the graphical genotyping results indicating that a series of progeny isolates lost one or more complete chromosomes, while other isolates received an extra copy of a particular chromosome.

In summary, the high-density mapping enabled the detection of meiotically driven and frequently occurring CNPs and CLPs in sexual progenies of the haploid plant pathogen *M. graminicola*. We identified 42 isolates that showed loss of a linkage group that was present in both parents compared to only two disomic isolates. Progenies showed 15 and 20% CNPs compared to the parents in the IPO323×IPO94269 and IPO323×IPO95052 crosses, respectively. Interestingly, the chromosomes lost were the same in both populations (Table S4). We performed 17 additional backcrosses and F_2_ crosses between progeny isolates that showed substantial CNPs. All crosses except one were successful and resulted in viable progeny (Table S15).

## Discussion

The genome of *M. graminicola* is highly plastic, based on the detailed analyses provided by the high-density genetic linkage maps. Eight chromosomes were missing in one or more progeny and can be considered dispensable, while other chromosomes occasionally were disomic. As many as three chromosomes were missing from individual progeny isolates, with no apparent effect on fitness. As expected, much of the genome plasticity is generated during meiosis and this could help to explain the high adaptability observed in field populations of this pathogen.

Dispensable chromosomes have been found in other fungi but they usually occur at a low frequency and typically represent single or a few chromosomes. For example the plant-pathogenic fungi *Alternaria alternata, Cochliobolus heterostrophus, Leptosphaeria maculans*, *Magnaporthe grisea* and *Nectria haematococca* as well as the insect pathogen *Metarhizium amisopliae* each had only a single chromosome that was dispensable [25]–[30]. Dispensable chromosomes in these species usually contain genes involved in pathogenicity or virulence [27], [28], [30], [31], whereas in others they don't [32]. In *M. graminicola*, genes involved in host plant perception did not map to any of the eight identified dispensable chromosomes [33]. Hence, the function of genes on dispensable chromosomes in *M. graminicola* is yet unknown.

Genome instability is a major cause of disorders, and a range of genes has been identified that have a role in maintaining genome integrity [34]. In addition, polyploidy and aneuploidy are considered evolutionary pathways to reproductive isolation and speciation [35], [36]. The mitotic and meiotic pairing and transmission of homologous chromosomes with length polymorphisms has been studied intensively in models such as the fungi *Saccharomyces cerevisiae*, *N. crassa* and *Coprinus cinereus* [15], [37], [38]. These model systems have substantially increased our knowledge of meiotic processes [39], but they mostly involved cytogenetic studies and mutant strains [40], [41]. A high-density genetic linkage map provides a strong genome-wide alternative for precise analyses of meiosis. However, the number of high-density genetic maps for fungi is limited due to difficulties and costs of high-quality marker generation and scoring required for their generation [42]. Here, we report the meiotic processing and generation of genomic plasticity using a high-density genetic linkage map for *M. graminicola*. This unusual approach enabled the detection of Mendelian and non-Mendelian inheritance patterns and elucidated the underlying meiotic principles that frequently resulted in progeny with CNPs.

It is very clear that meiosis not only maintains but also drives novel CNPs in *M. graminicola*, which most likely result from nondisjunction during the second meiotic division. We noticed that 15–20% of progeny isolates were missing one or more chromosomes that were present in the two parents. Interestingly, the same chromosomes were dispensable in both crosses. PCR analyses confirmed most of the CNPs, including the disomic chromosomes. Despite graphical genotyping indications for the absence of LGs 21 and B in the *M. graminicola* IPO323×IPO95052 progeny, PCR amplifications with several primer combinations derived from the mapped DArT markers on these LGs were inconclusive, although BLAST analyses to the genome of IPO323 revealed that they are single copy. The cause is unknown but may be due to the high repetitive content of these LGs (not shown).

The high number of markers on the current linkage map enabled accurate identification of twin isolates. These originate from the mitotic division after meiosis and provided a unique opportunity to test the meiotic origin of CNPs. If CNPs resulted from aberrations during mitosis, twin isolates would show differences in chromosome number and could not have been identified. In *M. graminicola*, we repeatedly observed the loss of the same chromosome in both twin isolates, which demonstrates it was lost during meiosis and that CNPs are mitotically stable. We cannot exclude the possibility of occasional mitotic instability between isolates that otherwise would have been identified as twins, but if it occurs it appears to be very rare. Hence, we conclude that CNPs in *M. graminicola* are driven by meiosis. Nondisjunction during either meiotic division results in progeny with CNPs due to gains or losses of entire chromosomes. However, the number of CNPs is twice as high after nondisjunction during meiosis I compared to meiosis II. Moreover, besides chromosome loss, meiosis I results in heterozygous and meiosis II in homozygous disomy. Crossovers may result in heterozygozity for part of the chromosome only, but the dispensable chromosomes are small so crossovers occur less frequently. Our data revealed frequent loss of chromosomes, but we only rarely observed heterozygozity. This indicates that nondisjunction occurred preferentially during meiosis II. Unfortunately, our marker technology did not enable the quantitative determination of copy numbers to confirm homozygous disomy.

Meiotic processing of CNPs in other fungi varies. For the related ascomycete *Leptosphaeria maculans*, twin genotypes were also always identical in respect to the presence or absence of a dispensable chromosome [25]. This indicates that, similar to *M. graminicola*, the dispensable chromosome in *L. maculans* is mitotically stable. However, in the evolutionarily more distantly related ascomycete *Magnaporthe oryzae* [26], presence of a dispensable chromosome varied in twin isolates, indicating that mitotic transmission of dispensable chromosomes may be unstable in some ascomycetes.

Apart from these differences and the fact that *M. graminicola* has up to eight dispensable chromosomes, a most striking aspect is that the widespread CNPs - involving multiple chromosomes - in *M. graminicola* do not hamper sexual reproduction. Interestingly, one of the factors inhibiting female fertility in *M. grisea* is present on a dispensable chromosome [32]. We do not have such evidence for *M. graminicola*. Recent karyotyping experiments showed that isolate IPO323 has at least two additional chromosomes compared to IPO94269 [4]. Nevertheless, we were successful in crossing these two isolates and made 17 additional crosses between *M. graminicola* isolates that showed substantial CNPs. Chromosomes without a homologous partner cannot pair, will have zero recombination and might be expected to be lost during meiosis. However, our data indicate that in *M. graminicola* they are normally transmitted to progeny without distortion of the segregation ratio. For example, in the progeny of the IPO323×IPO94269 cross, 34 and 35 out of 60 isolates contained the dispensable LGs 21 and C, respectively. This shows that the CNPs present between the parents are maintained during meiosis and are transmitted to approximately 50% of the progeny. Neither LG showed evidence of recombination as indicated by zero genetic distance between markers. The segregation of the unique IPO323 markers on these LGs confirmed the results of previous karyotyping experiments, that individual dispensable chromosomes are transmitted intact through meiosis [4]. This may well be among the first examples of distributive disjunction in fungi, a process that involves separation and distribution of non-recombining or non-homologous chromosomes during meiosis that is commonly observed in *Drosophila*. In fungi distributive disjunction was shown in *S. cerevisiae* by crossing strains that were monosomic for non-homologous chromosome I and III [43]. In *M. graminicola* monosomic strains do not occur as the fungus is haploid, but the dispensable chromosomes were shown to segregate regularly. In *S. cerevisiae* distributive disjunction is considered to be extremely rare as monosomy does not frequently occur [43]. In *M graminicola*, it might be essential as this study shows that CNPs occur frequently and are generated during meiosis. It is unknown whether distributive disjunction in *M. graminicola* also complies with the physical interactions between non-homologous chromosomes as was observed in *S. cerevisiae* [44].

In contrast, all LGs in the entire progeny set of the IPO323×IPO95052 cross contain markers from both parents, indicating that all parental chromosomes have homologous partners. Hence, in this respect the differences between the two Dutch bread wheat isolates (IPO323 and IPO94269) seem to be larger than were those between IPO323 and the Algerian durum wheat isolate IPO95052, underscoring the extraordinarily large genetic differences within local populations of *M. graminicola* [12], [45].

CLPs have been observed in at least 37 fungal species and hence seem to be a common feature of fungal genomes [15]. Clearly, recombination between homologous chromosomes of unequal length can result in new chromosome size variants. Moreover, the pairing of repeated sequences, for instance resulting from transposons, on different chromosomes during meiosis may lead to translocations that may be an important cause of CLPs as opposed to CNPs [38]. Subtelomeric variable regions such as those in *M. grisea* are also a potential source of meiotically driven CLPs [46]. The observed translocations in this study, as well as those in previous analyses [8], [9], [47], [48], most likely are responsible for the widespread CLPs in the genome of *M. graminicola* [4], [8], [9].

Compared to CLPs, CNPs in other fungi are observed less frequently, have not been analyzed through a map-based approach, and are generally highly unstable. For instance, a minichromosome in *M. grisea* showed non-Mendelian inheritance, which was also observed in *L. maculans* whenever one parent missed such a chromosome [25], [32]. Crosses between *L. maculans* isolates that both carried this minichromosome resulted in CLPs [25]. Duplication of large chromosomal fragments in *S. cerevisiae* occasionally results in the formation of supernumerary chromosomes that are highly unstable during mitosis [36], [37]. In the usually haploid human pathogen *Cryptococcus neoformans*, CNPs occur frequently in diploid AD serotypes as a potential mechanism to overcome slow filamentous growth [49] and, more recently, CNPs were discovered resulting from the generation and subsequent breakage of a dicentric chromosome [50]. CNPs in haploid filamentous fungi such as *N. crassa* are generally either lethal or seriously impair the sexual phase [38]. Diploid and disomic isolates of *N. crassa*, originating from nondisjunction at meiosis I, are highly unstable and do not differ in rates and mechanisms of haploidization and mitotic crossing over [51]. Similarly, disomic strains in *A. nidulans* that resulted from nondisjunction in meiotic metaphase I also were vegetatively unstable [52], [53].

In contrast to other species, CNPs in *M. graminicola* are vegetatively stable. We hypothesize that the extraordinarily high chromosome number of the *M. graminicola* genome [4] may influence the frequency and fate of CNPs. The genome of *M. graminicola* (39.8 Mb) is in the same size range as those of *Magnaporthe oryzae* (41.6 Mb), *Fusarium graminearum* (36.5 Mb), *A. nidulans* (30.0 Mb) and *N. crassa* (39.2 Mb). However, the number of chromosomes in these fungi (N = 8, 4, 7 and 7, for *A. nidulans*, *F. graminearum*, *M. oryzae*, and *N. crassa*, respectively) is much lower than in *M. graminicola* (N = 21). Hence, loss of entire chromosomes in these organisms may be lethal due to the presence of essential genes. *M. graminicola* has the highest chromosome number and the smallest autosomes in filamentous ascomycetes [4]. The present study has revealed that *M. graminicola* also has the highest number of dispensable chromosomes that vary from 0.39 to 0.77 Mb, representing up to 38% of the chromosomal complement and approximately 12% of its genome size. The frequent loss of chromosomes in *M. graminicola* without noticeable effect on fitness may be due to their small size. Dispensable chromosomes in many other fungi carry functional genes that play an important role in host-pathogen interactions [27]–[29], [31], [54], [55]. In *M. graminicola*, loci controlling host-pathogen interactions were not mapped on dispensable chromosomes and substantial CNPs in progeny isolates - up to three chromosomes per isolate covering as much as 1.59 Mb - neither reduced pathogenicity nor sexual compatibility [14], [33]. Therefore, pathogenicity in *M. graminicola* does not appear to be influenced by dispensable chromosomes.

In summary, our map-based approach is unique in analyses of genomic plasticity and demonstrates that CNPs in *M. graminicola* are meiotically generated and occur at much higher frequencies than reported previously for any ascomycete. These aberrations were observed in two crosses between field strains [10]. Since the sexual cycle occurs continuously under field conditions it is likely that meiotically driven CNPs play an important role in the high level of genetic diversity [11], [12], [56] observed among isolates of *M. graminicola*. The total genome content of *M. graminicola* isolates varies between 32–40 Mb and each field isolate represents a unique karyotype [4], [9]. In this study we showed that in addition to CLPs resulting from translocations, CNPs originate from aberrations during meiosis, mostly by nondisjunction during meiosis II. We hypothesize that the plasticity of the *M. graminicola* genome, as characterized by its large and flexible set of dispensable chromosomes, plays an important role in yet unknown processes of adaptation. This is currently being addressed in a *M. graminicola* crossing program aiming at individuals with a minimal genome size that is devoid of any dispensable chromosome. Backcrosses of such individuals with parental isolates will enable the selection of progeny with individual dispensable chromosome additions. Such a set will contribute significantly to understanding the role of dispensable chromosomes in the life strategy of *M. graminicola*.

## Supporting Information

Text S1 Supplementary text
(0.06 MB DOC)

Figure S1 Co-linearity of genetic linkage maps for Mycosphaerella graminicola crosses IPO323×IPO95052 (left) and IPO323×IPO94269 (right) with a bridge map (middle) generated with markers that segregated in both crosses. Common markers are shown in bold and start with the prefix C, SSR markers are shown in blue and markers that are translocated in red. DArT markers were named according to phase of the marker (A = IPO323, B = IPO95052 or IPO94269), complexity reduction method used (BMR or HMR), and location in the spotting plate (e.g. BBMR_15L11). LG and AFLP nomenclature is according to Kema et al., 2002. Segregation distortion of the markers is indicated with * (P<0.05), ** (P<0.01), *** (P<0.005) or **** (P<0.001).
(0.21 MB PDF)

Figure S2 Alignment of linkage group 21 between the IPO323×IPO95052 cross (left) and the IPO323×IPO94269 cross (right) shows recombination in the former but not in the latter. This indicates absence of this linkage group in isolate IPO94269. For IPO323×IPO94269, only markers from IPO323 could be mapped on this linkage group, and no markers from IPO94269, confirming that IPO94269 lacks this linkage group. Lines are drawn between markers that segregated in both populations. Stars next to the markers for the IPO323×IPO94269 cross indicate segregation distortion of the markers; * (P<0.05), ** (P<0.01), *** (P<0.005) or **** (P<0.001).
(0.03 MB PDF)

Figure S3 Confirmation of chromosome loss by PCR amplification. A. Confirmation of loss of LG 8 and LG 12 by SSR amplification. Loci ac-0007 (LG 8) and gga-0001 (LG 12) confirm that these linkage groups are absent in the underlined progeny isolates from the crosses IPO323×IPO94269 and IPO323×IPO95052 as neither of the parental alleles are amplified. Isolates 1158 and 1179 are positive controls and SSR ag-0003 (LG 2) is a positive PCR control in all duplex reactions. B. Confirmation of loss of LGs 13, 15, A and C by PCR with primers developed from DArT marker sequence data in the underlined progeny isolates derived from crosses between M. graminicola IPO323×IPO94269 and IPO323×IPO95052. Isolates 1158 and 1179 are positive control isolates, except in LGs C and 13 that have isolates 1158/2026 and 2032/2033, respectively, as positive checks. For LG 15* the CABMR_07D07 DArT fragment (129 bp) was used as a positive PCR control, while for the other linkage groups DArT fragment AHMR_08O09 (728 bp) was used. C. Confirmation of loss of LG 8 by PCR with primers developed from DArT marker sequence data in underlined progeny isolates derived from crosses between M. graminicola IPO323×IPO94269 and IPO323×IPO95052. This figure is composed of eight panels that are individually divided by a central marker lane. The left part of each panel represents the three parental isolates of the mapping populations (IPO323, IPO94269 and IPO95052), two positive control isolates (1158/1179), and seven progeny isolates that lack LG 8. The right part of each panel links to Fig. 2D and represents the two parental isolates (IPO323 and IPO95052), two twin isolates (1103/1126), two mirror isolates (1128/1183) and two twin isolates that lack LG 8 (2137/2139). In all panels DArT fragment AHMR_08O09 is the positive control (top band in each panel, 728 bp, located on LG 15).
(1.60 MB PDF)

Table S1 Mycosphaerella graminicola progeny isolates (n = 76) from the IPO323×IPO94269 in planta cross, that was made on the susceptible bread wheat cultivar Obelisk, that were used for hybridization to the DArT arrays.
(0.05 MB DOC)

Table S2 Mycosphaerella graminicola progeny isolates (n = 164) from the IPO323×IPO95052 in planta crosses that were made on the bread wheat cultivar Obelisk and the durum wheat cultivar Inbar. Sixteen isolates (gray-shaded) were not used, leaving a total of 148 that were used in the construction of the genetic linkage map. The first two numbers indicate the year of isolation and the next three numbers the order of isolation.
(0.08 MB DOC)

Table S3 The adapter and primer oligonucleotide sequences used for generation of the genomic representation (cloning) from Mycosphaerella graminicola isolates IPO323 and IPO95052 and for hybridization to the micro-arrays (genotyping) of parental and progeny isolates.
(0.04 MB DOC)

Table S4 Overview of Mycosphaerella graminicola F1 isolates that lack one or more linkage groups compared to the parental isolates IPO323, IPO94269 and IPO95052.
(0.03 MB DOC)

Table S5 Primer sequences used to verify the absence of several linkage groups in some progeny isolates of the two crosses. The primers were developed using the sequences of the DArT markers located on these linkage groups.
(0.07 MB DOC)

Table S6 Primer sequences used to verify the disomy for linkage group 1, isolate #51. The primers were developed around InDels obtained by comparison of BAC-end sequences from parental isolate IPO94269 with the genome sequence of isolate IPO323.
(0.04 MB DOC)

Table S7 Overview of type and number of molecular markers that were scored in the progeny of the cross between Mycosphaerella graminicola isolates IPO323 and IPO94269 before and after grouping.
(0.03 MB DOC)

Table S8 Overview of type and number of molecular markers that were scored in the progeny of the cross between Mycosphaerella graminicola isolates IPO323 and IPO95052 before and after grouping.
(0.04 MB DOC)

Table S9 Identified twin isolates in the two progenies derived from crosses between either Mycosphaerella graminicola isolates IPO323 and IPO94269 or IPO323 and IPO95052.
(0.04 MB DOC)

Table S10 Scoring tables
(3.33 MB XLS)

Table S11 Overview of the number of markers for both crosses. Mapping was performed using the software package JoinMap 3.0.
(0.03 MB DOC)

Table S12 Graphical genotyping
(5.15 MB XLS)

Table S13 Alignment of the identified linkage groups in the Mycosphaerella graminicola IPO323×IPO94269 and IPO323×IPO95052 mapping populations with the identified chromosomes in the Mycosphaerella graminicola genome sequence.
(0.05 MB DOC)

Table S14 DArT and SSR markers that showed translocations between two genetic linkage maps derived from crosses between either Mycosphaerella graminicola isolates IPO323 and IPO94269 or IPO95052.
(0.03 MB DOC)

Table S15 Back crosses and intercrosses of M. graminicola IPO323×IPO94269 progeny isolates with isolates that either lost or gained specific chromosomes.
(0.05 MB DOC)

## References

[1] Davis RH, Perkins DD (2002). *Neurospora*: a model of model microbes.. Nat Rev Genet.

[2] Geiser DM, Timberlake WE, Arnold ML (1996). Loss of meiosis in Aspergillus.. Mol Biol Evol.

[3] Raju NB (1980). Meiosis and ascospore genesis in *Neurospora*.. Eur J Cell Biol.

[4] Mehrabi R, Taga M, Kema GHJ (2007). Electrophoretic and cytological karyotyping of the wheat pathogen *Mycosphaerella graminicola*.. Mycologia.

[5] Cuomo CA, Güldener U, Xu J-R, Trail F, Turgeon BG (2007). The *fusarium graminearum* genome reveals a link between localized polymorphism and pathogen specialization.. Science.

[6] Dean RA, Talbot NJ, Ebbole DJ, Farman ML, Mitchell TK (2005). The genome sequence of the rice blast fungus *Magnaporthe grisea*.. Nature.

[7] Hane JK, Lowe RG, Solomon PS, Tan KC, Schoch CL (2007). Dothideomycete Plant Interactions Illuminated by Genome Sequencing and EST Analysis of the Wheat Pathogen *Stagonospora nodorum*.. Plant Cell.

[8] Kema GHJ, Verstappen ECP, Waalwijk C, Bonants PJM, de Koning JRA, Lucas JA, Bowyer P, Anderson HM (1999). Genetics of biological and molecular markers in *Mycosphaerella graminicola*, the cause of Septoria tritici leaf blotch of wheat.. Septoria on Cereals: a Study of Pathosystems.

[9] McDonald BA, Martinez JP (1991). Chromosome length polymorphisms in a *Septoria tritici* population.. Curr Genet.

[10] Kema GHJ, Verstappen ECP, Todorova M, Waalwijk C (1996). Successful crosses and molecular tetrad and progeny analyses demonstrate heterothallism in *Mycosphaerella graminicola*.. Curr Genet.

[11] Kema GHJ, Annone JG, Sayoud R, van Silfhout CH, van Ginkel M (1996). Genetic variation for virulence and resistance in the wheat-*Mycosphaerella graminicola* pathosystem. I. Interactions between pathogen isolates and host cultivars.. Phytopathology.

[12] Zhan J, Pettway RE, McDonald BA (2003). The global genetic structure of the wheat pathogen *Mycosphaerella graminicola* is characterized by high nuclear diversity, low mitochondrial diversity, regular recombination, and gene flow.. Fungal Genet Biol.

[13] Goodwin SB, Waalwijk C, Kema GHJ, Arora DK, Khachatourians GG (2004). Genetics and Genomics of *Mycosphaerella graminicola*, a Model for the Dothideales. Applied Mycology & Biotechnology.

[14] Kema GHJ, Goodwin SB, Hamza S, Verstappen ECP, Cavaletto JR (2002). A combined amplified fragment length polymorphism and randomly amplified polymorphism DNA genetic kinkage map of *Mycosphaerella graminicola*, the septoria tritici leaf blotch pathogen of wheat.. Genetics.

[15] Zolan ME (1995). Chromosome-length polymorphism in fungi.. Microbiol rev.

[16] Wittenberg AHJ, van der Lee T, Cayla C, Kilian A, Visser RGF (2005). Validation of the high-throughput marker technology DArT using the model plant *Arabidopsis thaliana*.. Mol Gen Genomics.

[17] Wenzl P, Carling J, Kudrna D, Jaccoud D, Huttner E (2004). Diversity Arrays Technology (DArT) for whole-genome profiling of barley.. Proc Natl Acad Sci USA.

[18] Jaccoud D, Peng K, Feinstein D, Kilian A (2001). Diversity arrays: a solid state technology for sequence information independent genotyping.. Nucleic Acids Res.

[19] Semagn K, Bjornstad A, Skinnes H, Maroy AG, Tarkegne Y (2006). Distribution of DArT, AFLP, and SSR markers in a genetic linkage map of a doubled-haploid hexaploid wheat population.. Genome.

[20] Akbari M, Wenzl P, Caig V, Carling J, Xia L (2006). Diversity arrays technology (DArT) for high-throughput profiling of the hexaploid wheat genome.. Theor Appl Genet.

[21] Goodwin SB, van der Lee TA, Cavaletto JR, Te Lintel Hekkert B, Crane CF (2007). Identification and genetic mapping of highly polymorphic microsatellite loci from an EST database of the septoria tritici blotch pathogen *Mycosphaerella graminicola*.. Fungal Genet Biol.

[22] Owen PG, Pei M, Karp A, Royle DJ, Edwards KJ (1998). Isolation and characterization of microsatellite loci in the wheat pathogen *Mycosphaerella graminicola*.. Mol ecol.

[23] Stam P (1993). Construction of integrated genetic linkage maps by means of a new computer package: JoinMap.. Plant J.

[24] Voorrips RE (2002). MapChart: software for the graphical presentation of linkage maps and QTLs.. J Hered.

[25] Leclair S, Ansan-Melayah D, Rouxel T, Balesdent M (1996). Meiotic behaviour of the minichromosome in the phytopathogenic ascomycete *Leptosphaeria maculans*.. Curr Genet.

[26] Chuma I, Tosa Y, Taga M, Nakayashiki H, Mayama S (2003). Meiotic behavior of a supernumerary chromosome in *Magnaporthe oryzae*.. Curr genet.

[27] Han Y, Liu X, Benny U, Kistler HC, VanEtten HD (2001). Genes determining pathogenicity to pea are clustered on a supernumerary chromosome in the fungal plant pathogen *Nectria haematococca*.. The Plant Journal.

[28] Hatta R, Ito K, Hosaki Y, Tanaka T, Tanaka A (2002). A conditionally dispensable chromosome controls host-specific pathogenicity in the fungal plant pathogen *Alternaria alternata*.. Genetics.

[29] Tzeng TH, Lyngholm LK, Ford CF, Bronson CR (1992). A restriction fragment length polymorphism map and electrophoretic karyotype of the fungal maize pathogen *Cochliobolus heterostrophus*.. Genetics.

[30] Wang C, Skrobek A, Butt TM (2003). Concurrence of losing a chromosome and the ability to produce destruxins in a mutant of *Metarhizium anisopliae*.. FEMS Microbiology Letters.

[31] Garmaroodi HS, Taga M (2007). Duplication of a conditionally dispensable chromosome carrying pea pathogenicity (PEP) gene clusters in Nectria haematococca.. Mol Plant Microbe Interact.

[32] Orbach MJ, Chumley FG, Valent B (1996). Electrophoretic karyotypes of *Magnaporthe grisea* pathogens of diverse grasses.. Mol Plant Microbe Interact.

[33] Ware SB (2006). Aspects of sexual reproduction in *Mycosphaerella* species on wheat and barley: genetic studies on specificity, mapping, and fungicide resistance.. Aspects of sexual reproduction in *Mycosphaerella* species on wheat and barley: genetic studies on specificity, mapping, and fungicide resistance.

[34] Aguilera A, Gomez-Gonzalez B (2008). Genome instability: a mechanistic view of its causes and consequences.. Nat Rev Genet.

[35] Kohn LM (2005). Mechanisms of Fungal Speciation.. Annual Review of Phytopathology.

[36] Koszul R, Caburet S, Dujon B, Fischer G (2004). Eucaryotic genome evolution through the spontaneous duplication of large chromosomal segments.. Embo J.

[37] Koszul R, Dujon B, Fischer G (2006). Stability of large segmental duplications in the yeast genome.. Genetics.

[38] Perkins DD (1997). Chromosome rearrangements in Neurospora and other filamentous fungi.. Adv Genet.

[39] Perkins DD, Davis RH (2000). Neurospora at the millennium.. Fungal Genet Biol.

[40] Cutter VM (1951). The cytology of the fungi.. Annu Rev Microbiol.

[41] Hardham AR, Mitchell HJ (1998). Use of molecular cytology to study the structure and biology of phytopathogenic and mycorrhizal fungi.. Fungal Genet Biol.

[42] Hackett CA, Broadfoot LB (2003). Effects of genotyping errors, missing values and segregation distortion in molecular marker data on the construction of linkage maps.. Heredity.

[43] Guacci V, Kaback DB (1991). Distributive disjunction of authentic chromosomes in *Saccharomyces cerevisiae*.. Genetics.

[44] Loidl J, Scherthan H, Kaback DB (1994). Physical association between nonhomologous chromosomes precedes distributive disjunction in yeast.. Proc Natl Acad Sci U S A.

[45] Stukenbrock EH, Banke S, Javan-Nikkhah M, McDonald BA (2007). Origin and domestication of the fungal wheat pathogen *Mycosphaerella graminicola* via sympatric speciation.. Mol Biol Evol.

[46] Farman ML, Leong SA (1995). Genetic and Physical Mapping of Telomeres in the Rice Blast Fungus, *Magnaporthe grisea*.. Genetics.

[47] Goodwin SB, Cavaletto JR, Waalwijk C, Kema GHJ (2001). DNA fingerprint probe from *Mycosphaerella graminicola* identifies an active transposable element.. Phytopathology.

[48] Chen RS, McDonald BA (1996). Sexual reproduction plays a major role in the genetic structure of populations of the fungus *Mycosphaerella graminicola*.. Genetics.

[49] Lengeler KB, Cox GM, Heitman J (2001). Serotype AD strains of Cryptococcus neoformans are diploid or aneuploid and are heterozygous at the mating-type locus.. Infect Immun.

[50] Fraser JA, Huang JC, Pukkila-Worley R, Alspaugh JA, Mitchell TG (2005). Chromosomal translocation and segmental duplication in Cryptococcus neoformans.. Eukaryot Cell.

[51] Smith DA (1975). A mutant affecting meiosis in Neurospora.. Genetics.

[52] Bainbridge BW, Roper JA (1966). Observations on the effects of a chromosome duplication in *Aspergillus nidulans*.. J Gen Microbiol.

[53] Swart K, van Heemst D, Slakhorst M, Debets F, Heyting C (2001). Isolation and characterization of sexual sporulation mutants of *Aspergillus nidulans*.. Fungal Genet Biol.

[54] VanEtten H, Funnell-Baerg D, Wasmann C, McCluskey K (1994). Location of pathogenicity genes on dispensable chromosomes in *Nectria haematococca* MPVI.. Antonie Van Leeuwenhoek.

[55] Miao VP, Covert SF, VanEtten HD (1991). A fungal gene for antibiotic resistance on a dispensable (“B”) chromosome.. Science.

[56] Kema GHJ, Sayoud R, Annone JG, van Silfhout CH (1996). Genetic variation for virulence and resistance in the wheat-*Mycosphaerella graminicola* pathosystem. II. Analysis of interactions between pathogen isolates and host cultivars.. Phytopathology.

