# Supplementary material for: Meiosis Drives Extraordinary Genome Plasticity in the Haploid Fungal Plant Pathogen Mycosphaerella graminicola
Source: PLoS One. 2009 Jun 10;4(6):e5863. doi: 10.1371/journal.pone.0005863 (PMC2689623; doi:10.1371/journal.pone.0005863)
Supplement: Text S1 — Supplementary text (0.06 MB DOC) [file pone.0005863.s001.doc]

**Text S1**

**Construction and comparison of the linkage maps**

Segregation ratios of all markers were analyzed with JoinMap, version 3.0 [1]. Markers with segregation ratios significantly different from 1:1 (*P* < 0.01) were initially set aside, and linkage analysis using the Kosambi mapping function was performed on the remaining markers. Initial assignment to linkage groups (LGs) was based on the logarithm of the odds (LOD) ratio for each possible marker pair. We used LOD values in the range of 3–8; the final assembly of LGs was completed using a LOD value of 4 or higher. We used linkages with a recombination rate (REC) < 0.4, a map LOD value of 0.05 and a χ^2^-jump threshold of 5 for inclusion into the map and for the calculation of the linear order of the markers within a LG. Finally, we tested whether the markers with distorted segregation ratios contributed to the map using an iterative addition process from markers with little to substantial segregation distortions. After each JoinMap run, the map was inspected for changes in marker order and distance. When these were not disturbed and when synteny between the two parallel crosses (IPO323 x IPO94269 and IPO323 x IPO95052) was observed, the addition of markers with distorted segregation ratios to the map was accepted. The individual maps were inspected either in Excel or with the graphical genotyping software package GGT [2,3], which allowed detection of singletons and visualized the recombination events in all progeny.

Marker data were grouped and merged using a Perl script. For the IPO323 x IPO94269 cross, merging of the marker data from the twins resulted in a scoring table with 60 individuals and 1341 markers. Grouping of the markers resulted in 473 unique segregation patterns containing 297 DArT, 165 AFLP and 11 SSR markers. The global call rate (percentage of scored individuals) we obtained for this dataset was 99.1%.

For the IPO323 x IPO95052 cross, merging of the marker data from the twins resulted in a scoring table with 125 individuals. One isolate had a genotype call rate less than 95% and was therefore omitted from further analysis. This resulted in a scoring table of 124 individuals and 1162 markers. Grouping resulted in 496 unique segregation patterns containing 491 DArT markers, four SSR markers, and the marker that co-segregates with the (a)virulence locus *MgAvrStb6*. The global call rate for this dataset was 98.8%.

For the bridge map the scores for markers segregating in both populations were merged. This resulted in a dataset of 184 individuals with 389 markers yielding 263 unique segregation patterns. The global call rate for this dataset was 99.03 %.

Individual maps were constructed using Joinmap 3.0. In the IPO323 x IPO94269 cross, 29 of the 473 (6.1%) segregation patterns exhibited a significant distortion (P < 0.01; based on χ^2^) from the expected (1:1) Mendelian ratio. These markers were therefore set aside during the first phase of the map construction. Using a LOD threshold of 4.0, 443 out of the 444 (99.8%) markers could be grouped into 24 LGs. One marker (AHMR_07I02) was not grouped while two AFLP markers (BEGGMpAT_439 and AEGAMpAC_114) exceeded the threshold for the χ^2^ jump and therefore were not positioned. The order of the markers in all 24 LGs was determined in a single round of JoinMap, and different settings of the thresholds did not alter the marker order or distance. Subsequently, we tried to incorporate the 29 markers that showed segregation distortion, and 10 of them were positioned based on the criteria listed above.

In the IPO323 x IPO95052 cross, 38 of the 496 (7.7 %) segregation patterns exhibited a significant distortion (P < 0.01; based on χ^2^) from the expected 1:1 Mendelian ratio and were initially omitted from the mapping. Using LOD thresholds of 4.0 (1 group), 5.0 (2 groups) and 5.5 (20 groups) all markers were grouped into 23 LGs. The order of the markers on the 23 LGs was determined in a single round of JoinMap, and again different settings of the thresholds did not alter the order or distance of the markers. Subsequently, we tried to incorporate the 38 markers that showed segregation distortion, and 28 of them could be positioned.

For the construction of the bridge map, loci with strong segregation distortion (*P* < 0.005) were removed and linkage analysis was performed on the remaining markers. Groups were selected at LOD ≥ 5.5 and the order of the markers within the LGs was determined using the same settings as for the construction of the individual maps.

**DArT analysis**

DNA of *M. graminicola* isolates IPO323 and IPO95052 was used to construct two genomic representations for each isolate essentially as described previously [4]. Genomic representations were generated by digesting 100 ng of genomic DNA with 2 units of either *Hin*dIII or *Bam*HI in combination with the 4-base cutters *Mse*I and *Rsa*I (New England Biolabs; NEB, USA). Cloning adapters (Table S9) were simultaneously ligated to the complementary overhangs with T4 DNA ligase (NEB). A 1-μl aliquot of the ligation product was used as a template in a 50-μl amplification reaction using primers complementary to the adapter sequences and cycling conditions as described [5].

A 3072-clone library for IPO323 and IPO95052 was prepared for each of the *Hind*III-*Mse*I-*Rsa*I and the *Bam*HI-*Mse*I-*Rsa*I complexity-reduction methods [6] with the modifications described by Wenzl et al. [5]. PCR products were dried, washed once with 70 % ETOH, and re-suspended in 25 µl of spotting buffer. The amplification products were spotted in duplicate on polylysine coated slides (Erie Scientific, Portsmouth, NH, USA) using a MicroGrid II arrayer (Biorobotics, Cambridge, UK). After printing, the slides were processed by incubation in hot water (95°C) for 2 min., dipped in a 100 mM EDTA and 100 mM DTT solution and dried by centrifugation.

The genomic representations of individual progeny isolates to be hybridized as targets on the array were generated by applying the complexity-reduction methods [5,6], with the exception that genotyping adapters rather than cloning adapters were used (Table S9). The products of the 50-µl PCR reactions were concentrated 10 fold by precipitation with 1 volume of isopropanol and denatured at 95°C for 3 min. Each reaction was labeled with 0.1 µl of Cy3-dUTP using 1 µl of 500 µM random decamers (Amersham Biosciences, Castle Hill, NSW, Australia) and the exo^-^ Klenow fragment of *Escherichia coli* DNA polymerase I (NEB). In experiments for which DNA was isolated twice, a replicate target labeled with Cy5-dUTP was co-hybridized with the Cy3-dUTP labeled target to the same array. The polylinker fragment of the plasmid was used as a reference [6] and labeled with 6-FAM. Labeled representations, called targets, were denaturated, hybridized to microarrays overnight at 65°C, and slides were washed according to Wenzl et al. [5].

Typically, each experiment was comprised of 96 bar-coded slides that were scanned using a Tecan LS 300 (Grödig, Austria) confocal laser scanner. Each image pair (cy3-FAM or cy5-FAM) was stored directly in the database and analyzed subsequently with DArTSoft (version 7.4.1), a software package developed at DArT P/L. DArTSoft was used to both identify and score the markers that were polymorphic within each experiment as described previously [5,7-12]. The program computes several quality parameters for each clone [7] and markers were selected by simultaneously applying thresholds for four of those: P-value, call rate, reproducibility and polymorphism information content (PIC). Clones with P values ≥ 77%, call rate ≥ 80%, reproducibility ≥ 95% and PIC ≥ 0.3 were selected for both libraries.

1. Stam P (1993) Construction of integrated genetic linkage maps by means of a new computer package: JoinMap. Plant J 3: 739-744.

2. Berloo RV (1999) GGT: Software for the display of graphical genotypes. Journal of Heredity 90: 328-329.

3. Berloo RV (2008) GGT 2.0: versaltile software for the visualization and analysis of genetic data. J Hered 99: 232-236.

4. Wittenberg AHJ, van der Lee T, Cayla C, Kilian A, Visser RGF, et al. (2005) Validation of the high-throughput marker technology DArT using the model plant *Arabidopsis thaliana*. Mol Gen Genomics 274: 30-39.

5. Wenzl P, Carling J, Kudrna D, Jaccoud D, Huttner E, et al. (2004) Diversity Arrays Technology (DArT) for whole-genome profiling of barley. Proc Natl Acad Sci USA 101: 9915-9920.

6. Jaccoud D, Peng K, Feinstein D, Kilian A (2001) Diversity arrays: a solid state technology for sequence information independent genotyping. Nucleic Acids Res 29: E25.

7. Akbari M, Wenzl P, Caig V, Carling J, Xia L, et al. (2006) Diversity arrays technology (DArT) for high-throughput profiling of the hexaploid wheat genome. Theor Appl Genet 113: 1409-1420.

8. Wenzl P, Li H, Carling J, Zhou M, Raman H, et al. (2006) A high-density consensus map of barley linking DArT markers to SSR, RFLP and STS loci and agricultural traits. BMC Genomics 7: 206.

9. Yang S, Pang W, Ash G, Harper J, Carling J, et al. (2006) Low level of genetic diversity in cultivated Pigeonpea compared to its wild relatives is revealed by diversity arrays technology. Theor Appl Genet 113: 585-595.

10. Wenzl P, Raman H, Wang J, Zhou M, Huttner E, et al. (2007) A DArT platform for quantitative bulked segregant analysis. BMC Genomics 8: 196.

11. James KE, Schneider H, Ansell SW, Evers M, Robba L, et al. (2008) Diversity Arrays Technology (DArT) for Pan-Genomic Evolutionary Studies of Non-Model Organisms. PLoS ONE 3: e1682.

12. Mace ES, Xia L, Jordan DR, Halloran K, Parh DK, et al. (2008) DArT markers: diversity analyses and mapping in Sorghum bicolor. BMC Genomics 9: 26.
