# Supplementary material for: Meiosis Drives Extraordinary Genome Plasticity in the Haploid Fungal Plant Pathogen Mycosphaerella graminicola
Source: PLoS One. 2009 Jun 10;4(6):e5863. doi: 10.1371/journal.pone.0005863 (PMC2689623; doi:10.1371/journal.pone.0005863)
Supplement: Figure S1 — Co-linearity of genetic linkage maps for Mycosphaerella graminicola crosses IPO323×IPO95052 (left) and IPO323×IPO94269 (right) with a bridge map (middle) generated with markers that segregated in both crosses. Common markers are shown in bold and start with the prefix C, SSR markers are shown in blue and markers that are translocated in red. DArT markers were named according to phase of the marker (A = IPO323, B = IPO95052 or IPO94269), complexity reduction method used (BMR or HMR), and location in the spotting plate (e.g. BBMR_15L11). LG and AFLP nomenclature is according to Kema et al., 2002. Segregation distortion of the markers is indicated with * (P<0.05), ** (P<0.01), *** (P<0.005) or **** (P<0.001). (0.21 MB PDF) [file pone.0005863.s002.pdf]

Genetic linkage maps of *Mycosphaerella graminicola*

Left: Linkage groups from segregation of markers in progeny of the bread wheat-derived isolate IPO323 and the durum wheat-derived isolate IPO95052.

Middle: Linkage groups in bridge map generated with shared segregating markers from both crosses; integration made possible by the common shared parental isolate IPO323.

Right: Linkage groups from segregation of markers in progeny of the bread wheat-derived isolates IPO323 and IPO94269.

Figure 5

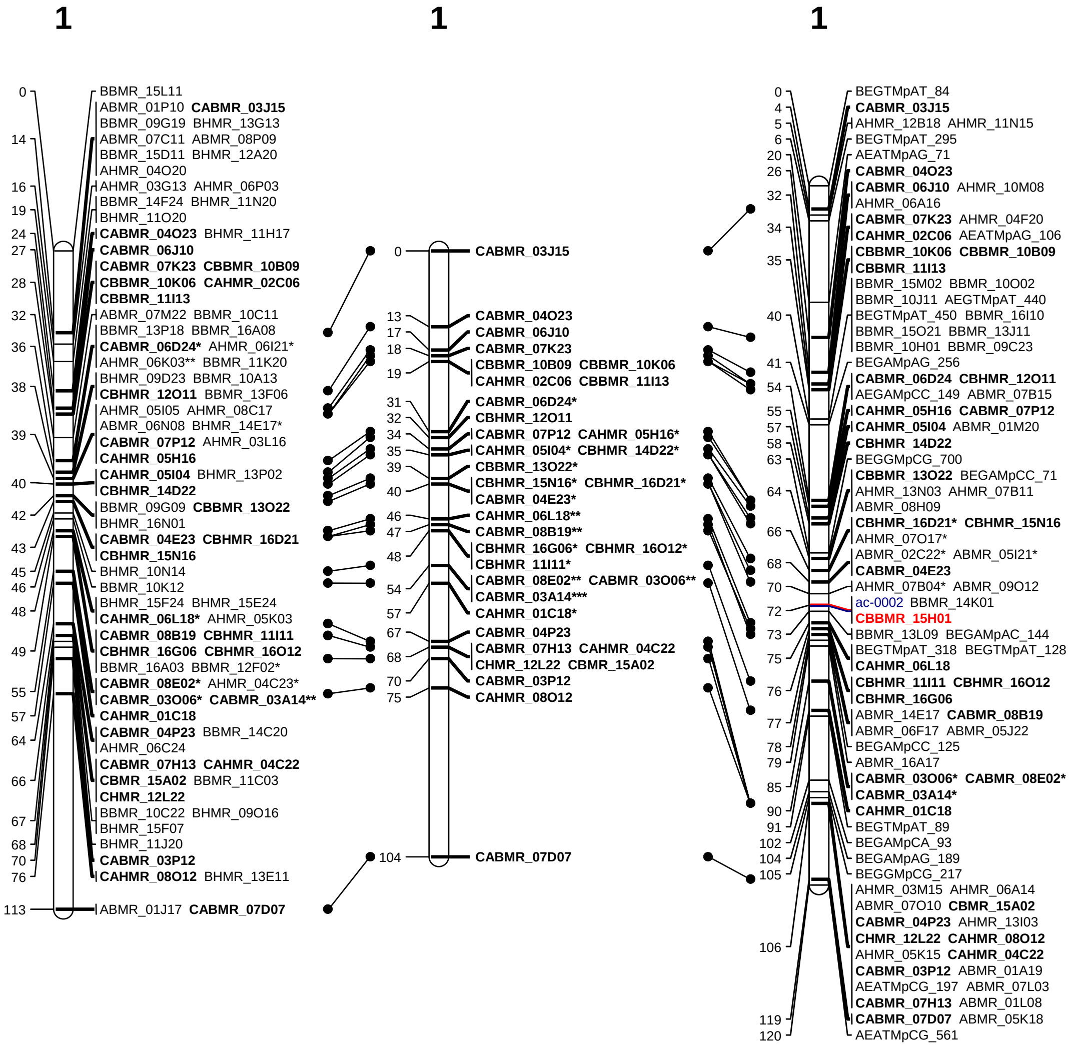
1
1
1
BBMR_15L11
ABMR_01P10 CABMR_03J15
BBMR_09G19 BHMR_13G13
ABMR_07C11 ABMR_08P09
BBMR_15D11 BHMR_12A20
AHMR_04O20
AHMR_03G13 AHMR_06P03
BBMR_14F24 BHMR_11N20
BHMR_11O20
CABMR_04O23 BHMR_11H17
CABMR_06J10
CABMR_07K23 CBBMR_10B09
CBBMR_10K06 CAHMR_02C06
CBBMR_11I13
ABMR_07M22 BBMR_10C11
BBMR_13P18 BBMR_16A08
CABMR_06D24* AHMR_06I21*
AHMR_06K03** BBMR_11K20
BHMR_09D23 BBMR_10A13
CBHMR_12O11 BBMR_13F06
AHMR_05I05 AHMR_08C17
ABMR_06N08 BHMR_14E17*
CABMR_07P12 AHMR_03L16
CAHMR_05H16
CAHMR_05I04 BHMR_13P02
CBHMR_14D22
BBMR_09G09 CBBMR_13O22
BHMR_16N01
CABMR_04E23 CBHMR_16D21
CBHMR_15N16
BHMR_10N14
BBMR_10K12
BHMR_15F24 BHMR_15E24
CAHMR_06L18* AHMR_05K03
CABMR_08B19 CBHMR_11I11
CBHMR_16G06 CBHMR_16O12
BBMR_16A03 BBMR_12F02*
CABMR_08E02* AHMR_04C23*
CABMR_03O06* CABMR_03A14**
CAHMR_01C18
CABMR_04P23 BBMR_14C20
AHMR_06C24
CABMR_07H13 CAHMR_04C22
CBMR_15A02 BBMR_11C03
CHMR_12L22
BBMR_10C22 BHMR_09O16
BHMR_15F07
BHMR_11J20
CABMR_03P12
CAHMR_08O12 BHMR_13E11
ABMR_01J17 CABMR_07D07
CABMR_03J15
CABMR_04O23
CABMR_06J10
CABMR_07K23
CBBMR_10B09 CBBMR_10K06
CAHMR_02C06 CBBMR_11I13
CABMR_06D24*
CBHMR_12O11
CABMR_07P12 CAHMR_05H16*
CAHMR_05I04* CBHMR_14D22*
CBBMR_13O22*
CBHMR_15N16* CBHMR_16D21*
CABMR_04E23*
CAHMR_06L18**
CABMR_08B19**
CBHMR_16G06* CBHMR_16O12*
CBHMR_11I11*
CABMR_08E02** CABMR_03O06**
CABMR_03A14***
CAHMR_01C18*
CABMR_04P23
CABMR_07H13 CAHMR_04C22
CHMR_12L22 CBMR_15A02
CABMR_03P12
CAHMR_08O12
CABMR_07D07
BEGTMpAT_84
CABMR_03J15
AHMR_12B18 AHMR_11N15
BEGTMpAT_295
AEATMpAG_71
CABMR_04O23
CABMR_06J10 AHMR_10M08
AHMR_06A16
CABMR_07K23 AHMR_04F20
CAHMR_02C06 AEATMpAG_106
CBBMR_10K06 CBBMR_10B09
CBBMR_11I13
BBMR_15M02 BBMR_10O02
BBMR_10J11 AEGTMpAT_440
BEGTMpAT_450 BBMR_16I10
BBMR_15O21 BBMR_13J11
BBMR_10H01 BBMR_09C23
BEGAMpAG_256
CABMR_06D24 CBHMR_12O11
AEGAMpCC_149 ABMR_07B15
CAHMR_05H16 CABMR_07P12
CAHMR_05I04 ABMR_01M20
CBHMR_14D22
BEGGMpCG_700
CBBMR_13O22 BEGAMpCC_71
AHMR_13N03 AHMR_07B11
ABMR_08H09
CBHMR_16D21* CBHMR_15N16
AHMR_07O17*
ABMR_02C22* ABMR_05I21*
CABMR_04E23
AHMR_07B04* ABMR_09O12
ac-0002 BBMR_14K01
CBBMR_15H01
BBMR_13L09 BEGAMpAC_144
BEGTMpAT_318 BEGTMpAT_128
CAHMR_06L18
CBHMR_11I11 CBHMR_16O12
CBHMR_16G06
ABMR_14E17 CABMR_08B19
ABMR_06F17 ABMR_05J22
BEGAMpCC_125
ABMR_16A17
CABMR_03O06* CABMR_08E02*
CABMR_03A14*
CAHMR_01C18
BEGTMpAT_89
BEGAMpCA_93
BEGAMpAG_189
BEGGMpCG_217
AHMR_03M15 AHMR_06A14
ABMR_07O10 CBMR_15A02
CABMR_04P23 AHMR_13I03
CHMR_12L22 CAHMR_08O12
AHMR_05K15 CAHMR_04C22
CABMR_03P12 ABMR_01A19
AEATMpCG_197 ABMR_07L03
CABMR_07H13 ABMR_01L08
CABMR_07D07 ABMR_05K18
AEATMpCG_561

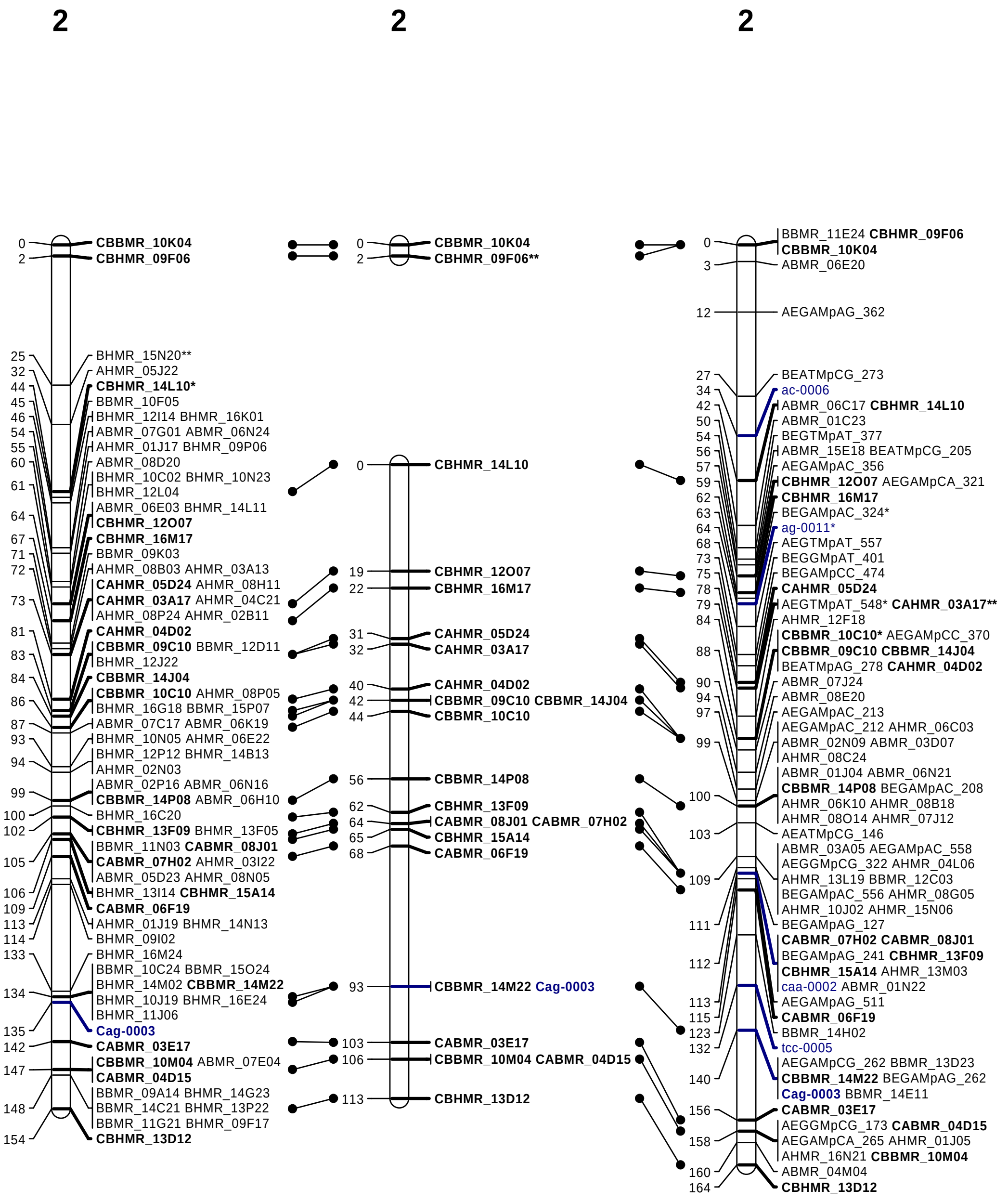
2
2
2
CBBMR_10K04
CBHMR_09F06
BHMR_15N20**
AHMR_05J22
CBHMR_14L10*
CBHMR_12O07
CBHMR_16M17
CAHMR_05D24
CAHMR_03A17
CAHMR_04D02
CBBMR_09C10
CBBMR_14J04
CBBMR_10C10
CBBMR_14P08
CBHMR_13F09
CABMR_08J01
CABMR_07H02
CBHMR_15A14
CABMR_06F19
CBBMR_14M22
Cag-0003
CABMR_03E17
CBBMR_10M04
CABMR_04D15
CBHMR_13D12
CBHMR_09F06**
ac-0006
ag-0011*
caa-0002
tcc-0005

Figure 5

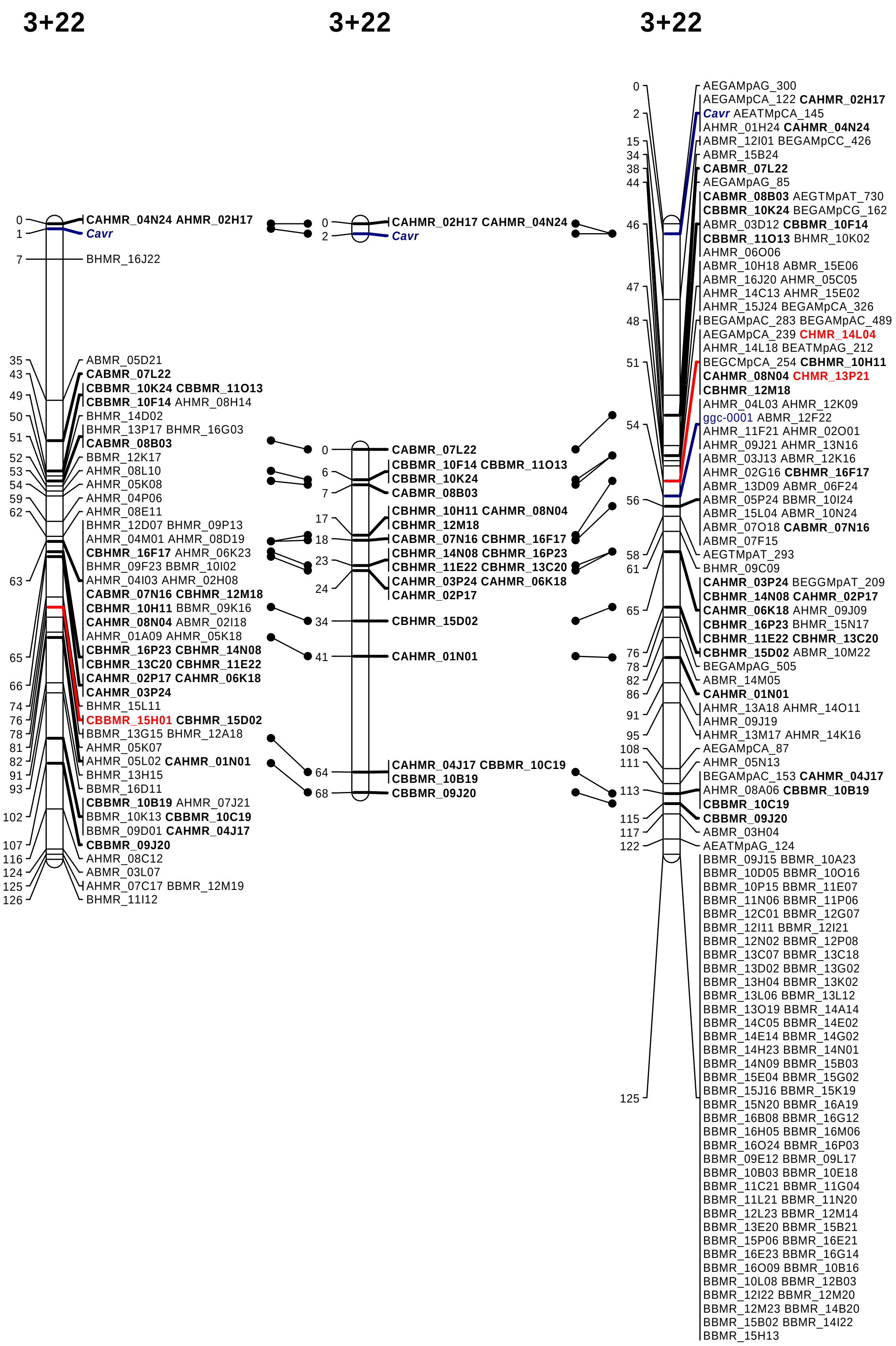

3+22
3+22
3+22
CAHMR_04N24 AHMR_02H17
Cavr
BHMR_16J22
ABMR_05D21
CABMR_07L22
CBBMR_10K24 CBBMR_11O13
CBBMR_10F14 AHMR_08H14
BHMR_14D02
BHMR_13P17 BHMR_16G03
CABMR_08B03
BBMR_12K17
AHMR_08L10
AHMR_05K08
AHMR_04P06
AHMR_08E11
BHMR_12D07 BHMR_09P13
AHMR_04M01 AHMR_08D19
CBHMR_16F17 AHMR_06K23
BHMR_09F23 BBMR_10I02
AHMR_04I03 AHMR_02H08
CABMR_07N16 CBHMR_12M18
CBHMR_10H11 BBMR_09K16
CAHMR_08N04 ABMR_02I18
AHMR_01A09 AHMR_05K18
CBHMR_16P23 CBHMR_14N08
CBHMR_13C20 CBHMR_11E22
CAHMR_02P17 CAHMR_06K18
CAHMR_03P24
BHMR_15L11
CBBMR_15H01 CBHMR_15D02
BBMR_13G15 BHMR_12A18
AHMR_05K07
AHMR_05L02 CAHMR_01N01
BHMR_13H15
BBMR_16D11
CBBMR_10B19 AHMR_07J21
BBMR_10K13 CBBMR_10C19
BBMR_09D01 CAHMR_04J17
CBBMR_09J20
AHMR_08C12
ABMR_03L07
AHMR_07C17 BBMR_12M19
BHMR_11I12
CAHMR_02H17 CAHMR_04N24
Cavr
CABMR_07L22
CBBMR_10F14 CBBMR_11O13
CBBMR_10K24
CABMR_08B03
CBHMR_10H11 CAHMR_08N04
CBHMR_12M18
CABMR_07N16 CBHMR_16F17
CBHMR_14N08 CBBMR_16P23
CBHMR_11E22 CBHMR_13C20
CAHMR_03P24 CAHMR_06K18
CAHMR_02P17
CBHMR_15D02
CAHMR_01N01
CAHMR_04J17 CBBMR_10C19
CBBMR_10B19
CBBMR_09J20
AEGAMpAG_300
AEGAMpCA_122 CAHMR_02H17
Cavr AEATMpCA_145
AHMR_01H24 CAHMR_04N24
ABMR_12I01 BEGAMpCC_426
ABMR_15B24
CABMR_07L22
AEGAMpAG_85
CABMR_08B03 AEGTMpAT_730
CBBMR_10K24 BEGAMpCG_162
ABMR_03D12 CBBMR_10F14
CBBMR_11O13 BHMR_10K02
AHMR_06O06
ABMR_10H18 ABMR_15E06
ABMR_16J20 AHMR_05C05
AHMR_14C13 AHMR_15E02
AHMR_15J24 BEGAMpCA_326
BEGAMpAC_283 BEGAMpAC_489
AEGAMpCA_239 CHMR_14L04
AHMR_14L18 BEATMpAG_212
BEGCMpCA_254 CBHMR_10H11
CAHMR_08N04 CHMR_13P21
CBHMR_12M18
AHMR_04L03 AHMR_12K09
ggc-0001 ABMR_12F22
AHMR_11F21 AHMR_02O01
AHMR_09J21 AHMR_13N16
ABMR_03J13 ABMR_12K16
AHMR_02G16 CBHMR_16F17
ABMR_13D09 ABMR_06F24
ABMR_05P24 BBMR_10I24
ABMR_15L04 ABMR_10N24
ABMR_07O18 CABMR_07N16
ABMR_07F15
AEGTMpAT_293
BHMR_09C09
CAHMR_03P24 BEGGMpAT_209
CBHMR_14N08 CAHMR_02P17
CAHMR_06K18 AHMR_09J09
CBHMR_16P23 BHMR_15N17
CBHMR_11E22 CBHMR_13C20
CBHMR_15D02 ABMR_10M22
BEGAMpAG_505
ABMR_14M05
CAHMR_01N01
AHMR_13A18 AHMR_14O11
AHMR_09J19
AHMR_13M17 AHMR_14K16
AEGAMpCA_87
AHMR_05N13
BEGAMpAC_153 CAHMR_04J17
AHMR_08A06 CBBMR_10B19
CBBMR_10C19
CBBMR_09J20
ABMR_03H04
AEATMpAG_124
BBMR_09J15 BBMR_10A23
BBMR_10D05 BBMR_10O16
BBMR_10P15 BBMR_11E07
BBMR_11N06 BBMR_11P06
BBMR_12C01 BBMR_12G07
BBMR_12I11 BBMR_12I21
BBMR_12N02 BBMR_12P08
BBMR_13C07 BBMR_13C18
BBMR_13D02 BBMR_13G02
BBMR_13H04 BBMR_13K02
BBMR_13L06 BBMR_13L12
BBMR_13O19 BBMR_14A14
BBMR_14C05 BBMR_14E02
BBMR_14E14 BBMR_14G02
BBMR_14H23 BBMR_14N01
BBMR_14N09 BBMR_15B03
BBMR_15E04 BBMR_15G02
BBMR_15J16 BBMR_15K19
BBMR_15N20 BBMR_16A19
BBMR_16B08 BBMR_16G12
BBMR_16H05 BBMR_16M06
BBMR_16O24 BBMR_16P03
BBMR_09E12 BBMR_09L17
BBMR_10B03 BBMR_10E18
BBMR_11C21 BBMR_11G04
BBMR_11L21 BBMR_11N20
BBMR_12L23 BBMR_12M14
BBMR_13E20 BBMR_15B21
BBMR_15P06 BBMR_16E21
BBMR_16E23 BBMR_16G14
BBMR_16O09 BBMR_10B16
BBMR_10L08 BBMR_12B03
BBMR_12I22 BBMR_12M20
BBMR_12M23 BBMR_14B20
BBMR_15B02 BBMR_14I22
BBMR_15H13

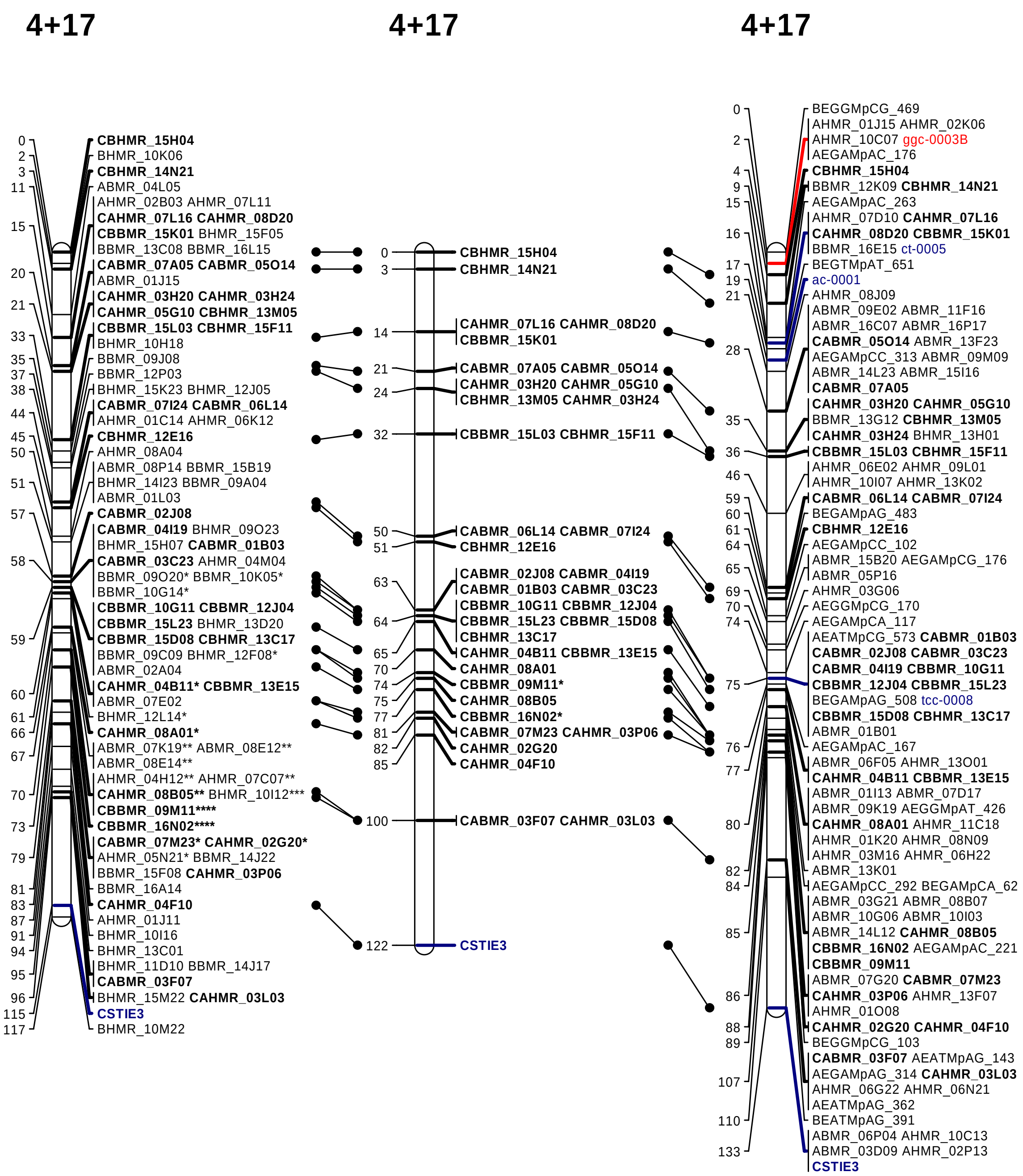
4+17
4+17
4+17
CBHMR_15H04
BHMR_10K06
CBHMR_14N21
ABMR_04L05
AHMR_02B03 AHMR_07L11
CAHMR_07L16 CAHMR_08D20
CBBMR_15K01 BHMR_15F05
BBMR_13C08 BBMR_16L15
CABMR_07A05 CABMR_05O14
ABMR_01J15
CAHMR_03H20 CAHMR_03H24
CAHMR_05G10 CBBMR_13M05
CBBMR_15L03 CBHMR_15F11
BHMR_10H18
BBMR_09J08
BBMR_12P03
BHMR_15K23 BHMR_12J05
CABMR_07I24 CABMR_06L14
AHMR_01C14 AHMR_06K12
CBHMR_12E16
AHMR_08A04
ABMR_08P14 BBMR_15B19
BHMR_14I23 BBMR_09A04
ABMR_01L03
CABMR_02J08
CABMR_04I19 BHMR_09O23
BHMR_15H07 CABMR_01B03
CABMR_03C23 AHMR_04M04
BBMR_09O20* BBMR_10K05*
BBMR_10G14*
CBBMR_10G11 CBBMR_12J04
CBBMR_15L23 BHMR_13D20
CBBMR_15D08 CBHMR_13C17
BBMR_09C09 BHMR_12F08*
ABMR_02A04
CAHMR_04B11* CBBMR_13E15
ABMR_07E02
BHMR_12L14*
CAHMR_08A01*
ABMR_07K19** ABMR_08E12**
ABMR_08E14**
AHMR_04H12** AHMR_07C07**
CAHMR_08B05** BHMR_10I12***
CBBMR_09M11****
CBBMR_16N02****
CABMR_07M23* CAHMR_02G20*
AHMR_05N21* BBMR_14J22
BBMR_15F08 CAHMR_03P06
BBMR_16A14
CAHMR_04F10
AHMR_01J11
BHMR_10I16
BHMR_13C01
BHMR_11D10 BBMR_14J17
CABMR_03F07
BHMR_15M22 CAHMR_03L03
CSTIE3
BHMR_10M22
CBHMR_15H04
CBHMR_14N21
CAHMR_07L16 CAHMR_08D20
CBBMR_15K01
CABMR_07A05 CABMR_05O14
CAHMR_03H20 CAHMR_05G10
CBBMR_13M05 CAHMR_03H24
CBBMR_15L03 CBHMR_15F11
CABMR_06L14 CABMR_07I24
CBHMR_12E16
CABMR_02J08 CABMR_04I19
CABMR_01B03 CABMR_03C23
CBBMR_10G11 CBBMR_12J04
CBBMR_15L23 CBBMR_15D08
CBHMR_13C17
CAHMR_04B11 CBBMR_13E15
CAHMR_08A01
CBBMR_09M11*
CAHMR_08B05
CBBMR_16N02*
CABMR_07M23 CAHMR_03P06
CAHMR_02G20
CAHMR_04F10
CABMR_03F07 CAHMR_03L03
CSTIE3
BEGGMpCG_469
AHMR_01J15 AHMR_02K06
AHMR_10C07 ggc-0003B
AEGAMpAC_176
CBHMR_15H04
BBMR_12K09 CBHMR_14N21
AEGAMpAC_263
AHMR_07D10 CAHMR_07L16
CAHMR_08D20 CBBMR_15K01
BBMR_16E15 ct-0005
BEGTMpAT_651
ac-0001
AHMR_08J09
ABMR_09E02 ABMR_11F16
ABMR_16C07 ABMR_16P17
CABMR_05O14 ABMR_13F23
AEGAMpCC_313 ABMR_09M09
ABMR_14L23 ABMR_15I16
CABMR_07A05
CAHMR_03H20 CAHMR_05G10
BBMR_13G12 CBBMR_13M05
CAHMR_03H24 BHMR_13H01
CBBMR_15L03 CBHMR_15F11
AHMR_06E02 AHMR_09L01
AHMR_10I07 AHMR_13K02
CABMR_06L14 CABMR_07I24
BEGAMpAG_483
CBHMR_12E16
AEGAMpCC_102
ABMR_15B20 AEGAMpCG_176
ABMR_05P16
AHMR_03G06
AEGGMpCG_170
AEGAMpCA_117
AEATMpCG_573 CABMR_01B03
CABMR_02J08 CABMR_03C23
CABMR_04I19 CBBMR_10G11
CBBMR_12J04 CBBMR_15L23
BEGAMpAG_508 tcc-0008
CBBMR_15D08 CBHMR_13C17
ABMR_01B01
AEGAMpAC_167
ABMR_06F05 AHMR_13O01
CAHMR_04B11 CBBMR_13E15
ABMR_01I13 ABMR_07D17
ABMR_09K19 AEGGMpAT_426
CAHMR_08A01 AHMR_11C18
AHMR_01K20 AHMR_08N09
AHMR_03M16 AHMR_06H22
ABMR_13K01
AEGAMpCC_292 BEGAMpCA_62
ABMR_03G21 ABMR_08B07
ABMR_10G06 ABMR_10I03
ABMR_14L12 CAHMR_08B05
CBBMR_16N02 AEGAMpAC_221
CBBMR_09M11
ABMR_07G20 CABMR_07M23
CAHMR_03P06 AHMR_13F07
AHMR_01O08
CAHMR_02G20 CAHMR_04F10
BEGGMpCG_103
CABMR_03F07 AEATMpAG_143
AEGAMpAG_314 CAHMR_03L03
AHMR_06G22 AHMR_06N21
AEATMpAG_362
BEATMpAG_391
ABMR_06P04 AHMR_10C13
ABMR_03D09 AHMR_02P13
CSTIE3

Figure 5

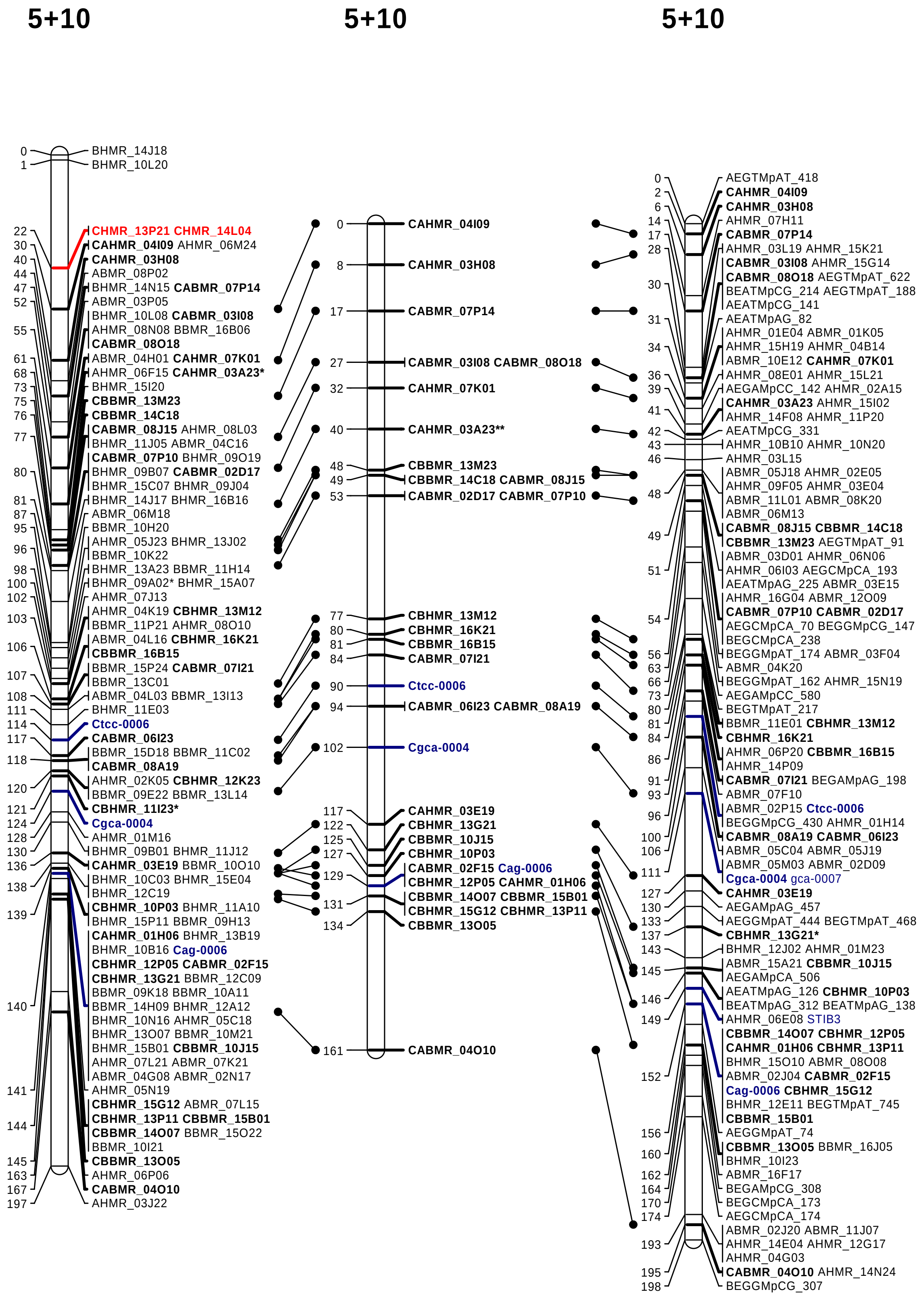
5+10
0 1 22 30 40 44 47 52 55 61 68 73 75 76 77 80 81 87 95 96 98 100 102 103 106 107 108 111 114 117 118 120 121 124 128 130 136 138 139 140 141 144 145 163 167 197
BHMR_14J18
BHMR_10L20
CHMR_13P21 CHMR_14L04
CAHMR_04I09 AHMR_06M24
CAHMR_03H08
ABMR_08P02
BHMR_14N15 CABMR_07P14
ABMR_03P05
BHMR_10L08 CABMR_03I08
AHMR_08N08 BBMR_16B06
CABMR_08O18
ABMR_04H01 CAHMR_07K01
AHMR_06F15 CAHMR_03A23*
BHMR_15I20
CBBMR_13M23
CBBMR_14C18
CABMR_08J15 AHMR_08L03
BHMR_11J05 ABMR_04C16
CABMR_07P10 BHMR_09O19
BHMR_09B07 CABMR_02D17
BHMR_15C07 BHMR_09J04
BHMR_14J17 BHMR_16B16
ABMR_06M18
BBMR_10H20
AHMR_05J23 BHMR_13J02
BBMR_10K22
BHMR_13A23 BBMR_11H14
BHMR_09A02* BHMR_15A07
AHMR_07J13
AHMR_04K19 CBHMR_13M12
BBMR_11P21 AHMR_08O10
ABMR_04L16 CBHMR_16K21
CBBMR_16B15
BBMR_15P24 CABMR_07I21
BBMR_13C01
ABMR_04L03 BBMR_13I13
BHMR_11E03
Ctcc-0006
CABMR_06I23
BBMR_15D18 BBMR_11C02
CABMR_08A19
AHMR_02K05 CBHMR_12K23
BBMR_09E22 BBMR_13L14
CBHMR_11I23*
Cgca-0004
AHMR_01M16
BHMR_09B01 BHMR_11J12
CAHMR_03E19 BBMR_10O10
BHMR_10C03 BHMR_15E04
BHMR_12C19
CBHMR_10P03 BHMR_11A10
BHMR_15P11 BBMR_09H13
CAHMR_01H06 BHMR_13B19
BHMR_10B16 Cag-0006
CBHMR_12P05 CABMR_02F15
CBHMR_13G21 BBMR_12C09
BBMR_09K18 BBMR_10A11
BBMR_14H09 BHMR_12A12
BHMR_10N16 AHMR_05C18
BHMR_13O07 BBMR_10M21
BHMR_15B01 CBBMR_10J15
AHMR_07L21 ABMR_07K21
ABMR_04G08 ABMR_02N17
AHMR_05N19
CBHMR_15G12 ABMR_07L15
CBHMR_13P11 CBBMR_15B01
CBBMR_14O07 BBMR_15O22
BBMR_10I21
CBBMR_13O05
AHMR_06P06
CABMR_04O10
AHMR_03J22
5+10
0 CAHMR_04I09
8 CAHMR_03H08
17 CABMR_07P14
27 CABMR_03I08 CABMR_08O18
32 CAHMR_07K01
40 CAHMR_03A23**
48 CBBMR_13M23
49 CBBMR_14C18 CABMR_08J15
53 CABMR_02D17 CABMR_07P10
77 CBHMR_13M12
80 CBHMR_16K21
81 CBBMR_16B15
84 CABMR_07I21
90 Ctcc-0006
94 CABMR_06I23 CABMR_08A19
102 Cgca-0004
117 CAHMR_03E19
122 CBHMR_13G21
125 CBBMR_10J15
127 CBHMR_10P03
CABMR_02F15 Cag-0006
129 CBHMR_12P05 CAHMR_01H06
CBBMR_14O07 CBBMR_15B01
131 CBHMR_15G12 CBHMR_13P11
134 CBBMR_13O05
161 CABMR_04O10
5+10
0 2 6 14 17 28 30 31 34 36 39 41 42 43 46 48 49 51 54 56 63 66 73 80 81 84 86 91 93 96 100 106 111 127 130 133 137 143 145 146 149 152 156 160 162 164 170 174 193 195 198
AEGTMpAT_418
CAHMR_04I09
CAHMR_03H08
AHMR_07H11
CABMR_07P14
AHMR_03L19 AHMR_15K21
CABMR_03I08 AHMR_15G14
CABMR_08O18 AEGTMpAT_622
BEATMpCG_214 AEGTMpAT_188
AEATMpCG_141
AEATMpAG_82
AHMR_01E04 ABMR_01K05
AHMR_15H19 AHMR_04B14
ABMR_10E12 CAHMR_07K01
AHMR_08E01 AHMR_15L21
AEGAMpCC_142 AHMR_02A15
CAHMR_03A23 AHMR_15I02
AHMR_14F08 AHMR_11P20
AEATMpCG_331
AHMR_10B10 AHMR_10N20
AHMR_03L15
ABMR_05J18 AHMR_02E05
AHMR_09F05 AHMR_03E04
ABMR_11L01 ABMR_08K20
ABMR_06M13
CABMR_08J15 CBBMR_14C18
CBBMR_13M23 AEGTMpAT_91
ABMR_03D01 AHMR_06N06
AHMR_06I03 AEGCMpCA_193
AEATMpAG_225 ABMR_03E15
AHMR_16G04 ABMR_12O09
CABMR_07P10 CABMR_02D17
AEGCMpCA_70 BEGGMpCG_147
BEGCMpCA_238
BEGGMpAT_174 ABMR_03F04
ABMR_04K20
BEGGMpAT_162 AHMR_15N19
AEGAMpCC_580
BEGTMpAT_217
BBMR_11E01 CBHMR_13M12
CBHMR_16K21
AHMR_06P20 CBBMR_16B15
AHMR_14P09
CABMR_07I21 BEGAMpAG_198
ABMR_07F10
ABMR_02P15 Ctcc-0006
BEGGMpCG_430 AHMR_01H14
CABMR_08A19 CABMR_06I23
ABMR_05C04 ABMR_05J19
ABMR_05M03 ABMR_02D09
Cgca-0004 gca-0007
CAHMR_03E19
AEGAMpAG_457
AEGGMpAT_444 BEGTMpAT_468
CBHMR_13G21*
BHMR_12J02 AHMR_01M23
ABMR_15A21 CBBMR_10J15
AEGAMpCA_506
AEATMpAG_126 CBHMR_10P03
BEATMpAG_312 BEATMpAG_138
AHMR_06E08 STIB3
CBBMR_14O07 CBHMR_12P05
CAHMR_01H06 CBHMR_13P11
BHMR_15O10 ABMR_08O08
ABMR_02J04 CABMR_02F15
Cag-0006 CBHMR_15G12
BHMR_12E11 BEGTMpAT_745
CBBMR_15B01
AEGGMpAT_74
CBBMR_13O05 BBMR_16J05
BHMR_10I23
ABMR_16F17
BEGAMpCG_308
BEGCMpCA_173
AEGCMpCA_174
ABMR_02J20 ABMR_11J07
AHMR_14E04 AHMR_12G17
AHMR_04G03
CABMR_04O10 AHMR_14N24
BEGGMpCG_307

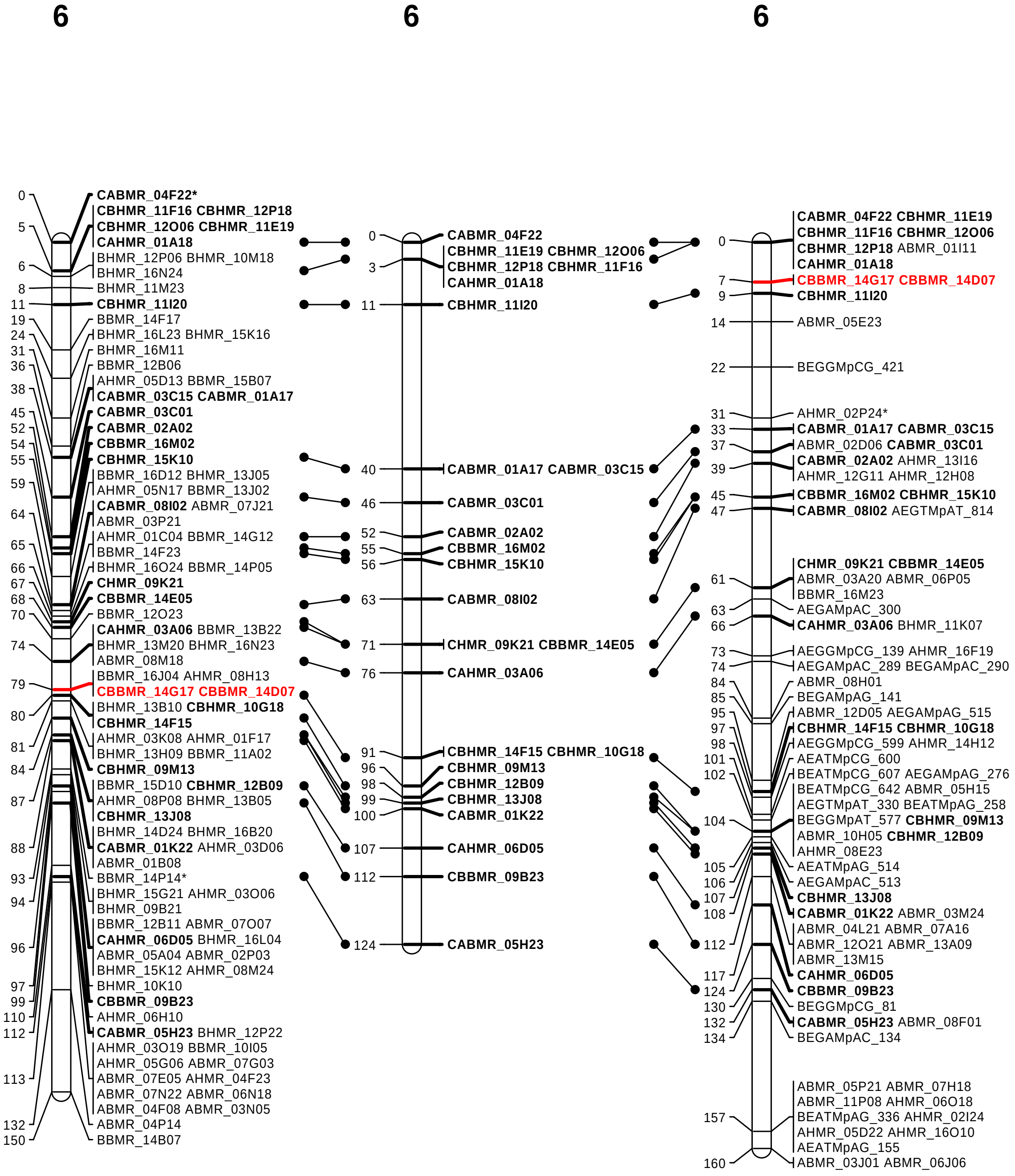
6
6
6
CABMR_04F22*
CBHMR_11F16 CBHMR_12P18
CBHMR_12O06 CBHMR_11E19
CAHMR_01A18
BHMR_12P06 BHMR_10M18
BHMR_16N24
BHMR_11M23
CBHMR_11I20
BBMR_14F17
BHMR_16L23 BHMR_15K16
BHMR_16M11
BBMR_12B06
AHMR_05D13 BBMR_15B07
CABMR_03C15 CABMR_01A17
CABMR_03C01
CABMR_02A02
CBBMR_16M02
CBHMR_15K10
BBMR_16D12 BHMR_13J05
AHMR_05N17 BBMR_13J02
CABMR_08I02 ABMR_07J21
ABMR_03P21
AHMR_01C04 BBMR_14G12
BBMR_14F23
BHMR_16O24 BBMR_14P05
CHMR_09K21
CBBMR_14E05
BBMR_12O23
CAHMR_03A06 BBMR_13B22
BHMR_13M20 BHMR_16N23
ABMR_08M18
BBMR_16J04 AHMR_08H13
CBBMR_14G17 CBBMR_14D07
BHMR_13B10 CBHMR_10G18
CBHMR_14F15
AHMR_03K08 AHMR_01F17
BHMR_13H09 BBMR_11A02
CBHMR_09M13
BBMR_15D10 CBHMR_12B09
AHMR_08P08 BHMR_13B05
CBHMR_13J08
BHMR_14D24 BHMR_16B20
CABMR_01K22 AHMR_03D06
ABMR_01B08
BBMR_14P14*
BHMR_15G21 AHMR_03O06
BHMR_09B21
BBMR_12B11 ABMR_07O07
CAHMR_06D05 BHMR_16L04
ABMR_05A04 ABMR_02P03
BHMR_15K12 AHMR_08M24
BHMR_10K10
CBBMR_09B23
AHMR_06H10
CABMR_05H23 BHMR_12P22
AHMR_03O19 BBMR_10I05
AHMR_05G06 ABMR_07G03
ABMR_07E05 AHMR_04F23
ABMR_07N22 ABMR_06N18
ABMR_04F08 ABMR_03N05
ABMR_04P14
BBMR_14B07
0 5 6 8 11 19 24 31 36 38 45 52 54 55 59 64 65 66 67 68 70 74 79 80 81 84 87 88 93 94 96 97 99 110 112 113 132 150
CABMR_04F22
CBHMR_11E19 CBHMR_12O06
CBHMR_12P18 CBHMR_11F16
CAHMR_01A18
CBHMR_11I20
CABMR_01A17 CABMR_03C15
CABMR_03C01
CABMR_02A02
CBBMR_16M02
CBHMR_15K10
CABMR_08I02
CHMR_09K21 CBBMR_14E05
CAHMR_03A06
CBHMR_14F15 CBHMR_10G18
CBHMR_09M13
CBHMR_12B09
CBHMR_13J08
CABMR_01K22
CAHMR_06D05
CBBMR_09B23
CABMR_05H23
0 3 11 40 46 52 55 56 63 71 76 91 96 98 99 100 107 112 124
CABMR_04F22 CBHMR_11E19
CBHMR_11F16 CBHMR_12O06
CBHMR_12P18 ABMR_01I11
CAHMR_01A18
CBBMR_14G17 CBBMR_14D07
CBHMR_11I20
ABMR_05E23
BEGGMpCG_421
AHMR_02P24*
CABMR_01A17 CABMR_03C15
ABMR_02D06 CABMR_03C01
CABMR_02A02 AHMR_13I16
AHMR_12G11 AHMR_12H08
CBBMR_16M02 CBHMR_15K10
CABMR_08I02 AEGTMpAT_814
CHMR_09K21 CBBMR_14E05
ABMR_03A20 ABMR_06P05
BBMR_16M23
AEGAMpAC_300
CAHMR_03A06 BHMR_11K07
AEGGMpCG_139 AHMR_16F19
AEGAMpAC_289 BEGAMpAC_290
ABMR_08H01
BEGAMpAG_141
ABMR_12D05 AEGAMpAG_515
CBHMR_14F15 CBHMR_10G18
AEGGMpCG_599 AHMR_14H12
AEATMpCG_600
BEATMpCG_607 AEGAMpAG_276
BEATMpCG_642 ABMR_05H15
AEGTMpAT_330 BEATMpAG_258
BEGGMpAT_577 CBHMR_09M13
ABMR_10H05 CBHMR_12B09
AHMR_08E23
AEATMpAG_514
AEGAMpAC_513
CBHMR_13J08
CABMR_01K22 ABMR_03M24
ABMR_04L21 ABMR_07A16
ABMR_12O21 ABMR_13A09
ABMR_13M15
CAHMR_06D05
CBBMR_09B23
BEGGMpCG_81
CABMR_05H23 ABMR_08F01
BEGAMpAC_134
ABMR_05P21 ABMR_07H18
ABMR_11P08 AHMR_06O18
BEATMpAG_336 AHMR_02I24
AHMR_05D22 AHMR_16O10
AEATMpAG_155
ABMR_03J01 ABMR_06J06
0 7 9 14 22 31 33 37 39 45 47 61 63 66 73 74 84 85 95 97 98 101 102 104 105 106 107 108 112 117 124 130 132 134 157 160

Figure 5

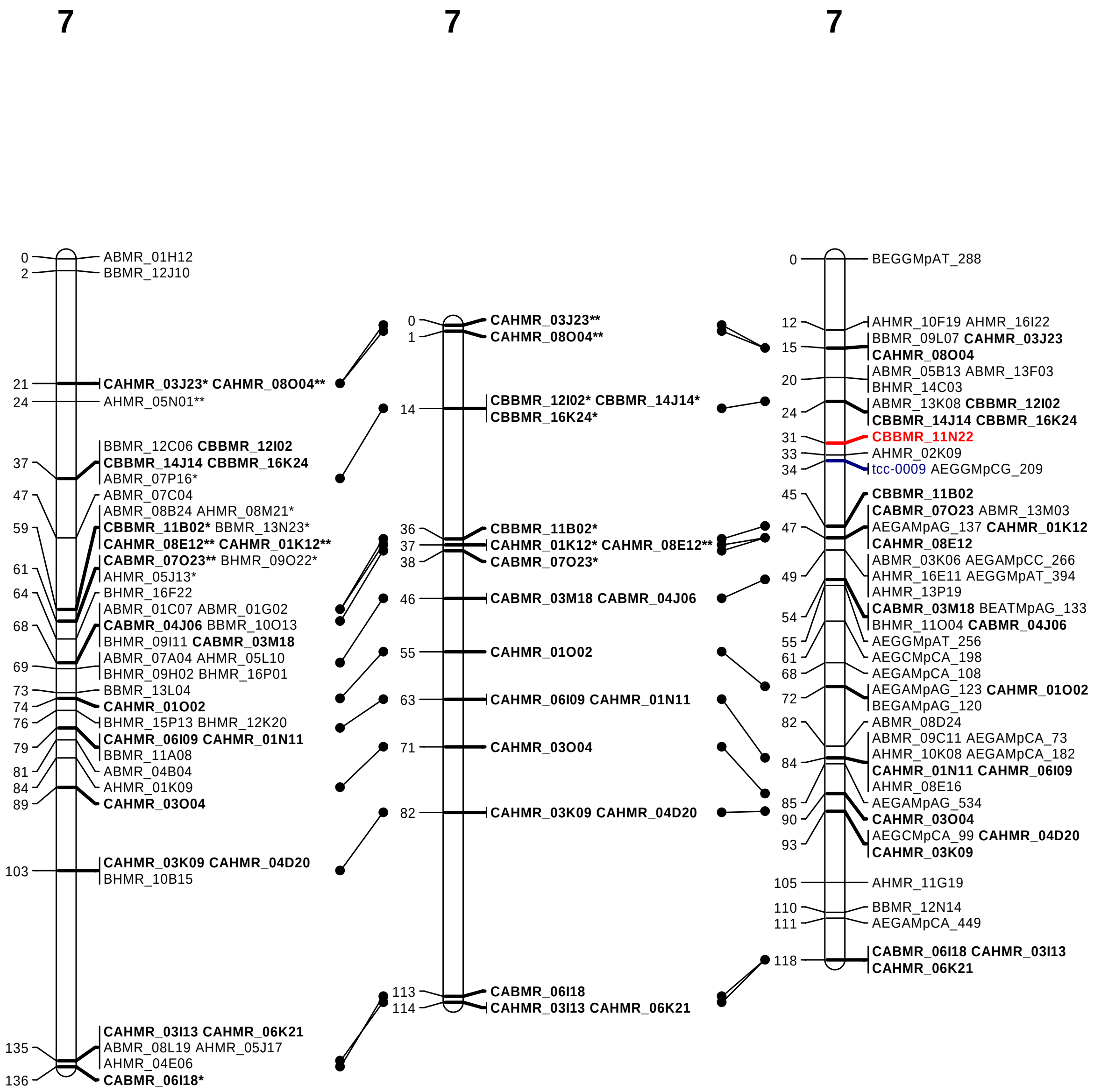

7
7
7
ABMR_01H12
BBMR_12J10
CAHMR_03J23* CAHMR_08O04**
AHMR_05N01**
BBMR_12C06 CBBMR_12I02
CBBMR_14J14 CBBMR_16K24
ABMR_07P16*
ABMR_07C04
ABMR_08B24 AHMR_08M21*
CBBMR_11B02* BBMR_13N23*
CAHMR_08E12** CAHMR_01K12**
CABMR_07O23** BHMR_09O22*
AHMR_05J13*
BHMR_16F22
ABMR_01C07 ABMR_01G02
CABMR_04J06 BBMR_10O13
BHMR_09I11 CABMR_03M18
ABMR_07A04 AHMR_05L10
BHMR_09H02 BHMR_16P01
BBMR_13L04
CAHMR_01O02
BHMR_15P13 BHMR_12K20
CAHMR_06I09 CAHMR_01N11
BBMR_11A08
ABMR_04B04
AHMR_01K09
CAHMR_03O04
CAHMR_03K09 CAHMR_04D20
BHMR_10B15
CAHMR_03I13 CAHMR_06K21
ABMR_08L19 AHMR_05J17
AHMR_04E06
CABMR_06I18*
CAHMR_03J23**
CAHMR_08O04**
CBBMR_12I02* CBBMR_14J14*
CBBMR_16K24*
CBBMR_11B02*
CAHMR_01K12* CAHMR_08E12**
CABMR_07O23*
CABMR_03M18 CABMR_04J06
CAHMR_01O02
CAHMR_06I09 CAHMR_01N11
CAHMR_03O04
CAHMR_03K09 CAHMR_04D20
CABMR_06I18
CAHMR_03I13 CAHMR_06K21
BEGGMpAT_288
AHMR_10F19 AHMR_16I22
BBMR_09L07 CAHMR_03J23
CAHMR_08O04
ABMR_05B13 ABMR_13F03
BHMR_14C03
ABMR_13K08 CBBMR_12I02
CBBMR_14J14 CBBMR_16K24
CBBMR_11N22
AHMR_02K09
tcc-0009 AEGGMpCG_209
CBBMR_11B02
CABMR_07O23 ABMR_13M03
AEGAMpAG_137 CAHMR_01K12
CAHMR_08E12
ABMR_03K06 AEGAMpCC_266
AHMR_16E11 AEGGMpAT_394
AHMR_13P19
CABMR_03M18 BEATMpAG_133
BHMR_11O04 CABMR_04J06
AEGGMpAT_256
AEGCMpCA_198
AEGAMpCA_108
AEGAMpAG_123 CAHMR_01O02
BEGAMpAG_120
ABMR_08D24
ABMR_09C11 AEGAMpCA_73
AHMR_10K08 AEGAMpCA_182
CAHMR_01N11 CAHMR_06I09
AHMR_08E16
AEGAMpAG_534
CAHMR_03O04
AEGCMpCA_99 CAHMR_04D20
CAHMR_03K09
AHMR_11G19
BBMR_12N14
AEGAMpCA_449
CABMR_06I18 CAHMR_03I13
CAHMR_06K21

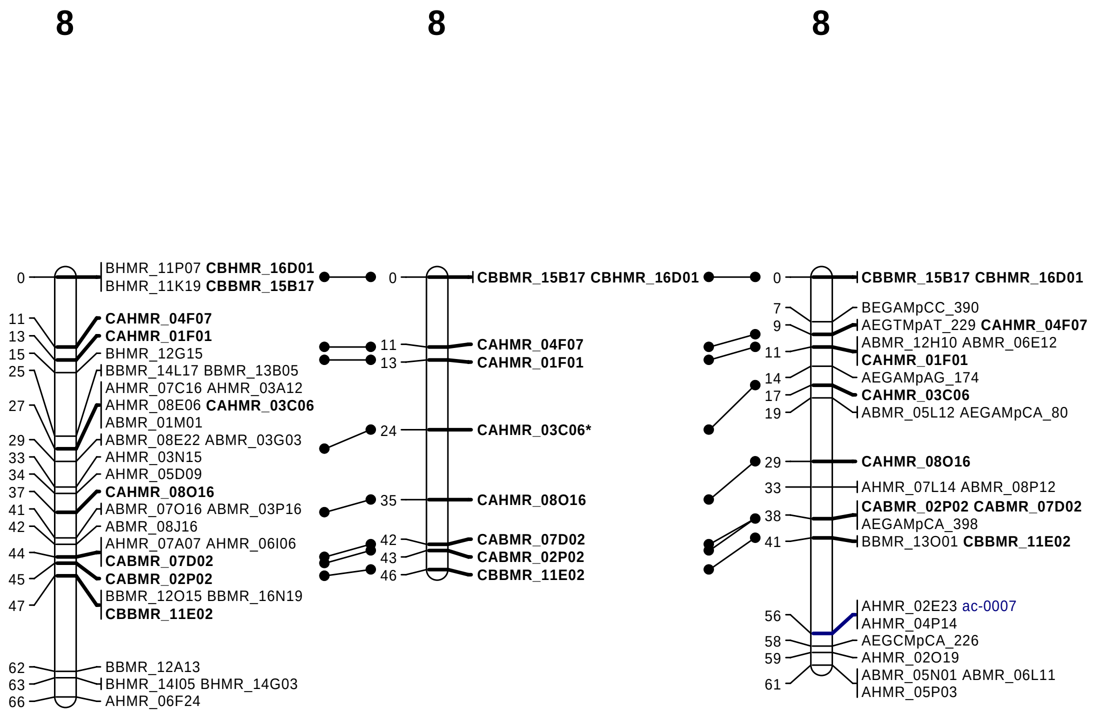
8
8
8
0 BHMR_11P07 CBHMR_16D01
BHMR_11K19 CBBMR_15B17
11 CAHMR_04F07
13 CAHMR_01F01
15 BHMR_12G15
25 BBMR_14L17 BBMR_13B05
27 AHMR_07C16 AHMR_03A12
AHMR_08E06 CAHMR_03C06
ABMR_01M01
29 ABMR_08E22 ABMR_03G03
33 AHMR_03N15
34 AHMR_05D09
37 CAHMR_08O16
41 ABMR_07O16 ABMR_03P16
42 ABMR_08J16
44 AHMR_07A07 AHMR_06I06
CABMR_07D02
45 CABMR_02P02
47 BBMR_12O15 BBMR_16N19
CBBMR_11E02
62 BBMR_12A13
63 BHMR_14I05 BHMR_14G03
66 AHMR_06F24
0 CBBMR_15B17 CBHMR_16D01
11 CAHMR_04F07
13 CAHMR_01F01
24 CAHMR_03C06*
35 CAHMR_08O16
42 CABMR_07D02
43 CABMR_02P02
46 CBBMR_11E02
0 CBBMR_15B17 CBHMR_16D01
7 BEGAMpCC_390
9 AEGTMpAT_229 CAHMR_04F07
11 ABMR_12H10 ABMR_06E12
CAHMR_01F01
14 AEGAMpAG_174
17 CAHMR_03C06
19 ABMR_05L12 AEGAMpCA_80
29 CAHMR_08O16
33 AHMR_07L14 ABMR_08P12
38 CABMR_02P02 CABMR_07D02
AEGAMpCA_398
41 BBMR_13O01 CBBMR_11E02
56 AHMR_02E23 ac-0007
AHMR_04P14
58 AEGCMpCA_226
59 AHMR_02O19
61 ABMR_05N01 ABMR_06L11
AHMR_05P03

Figure 5

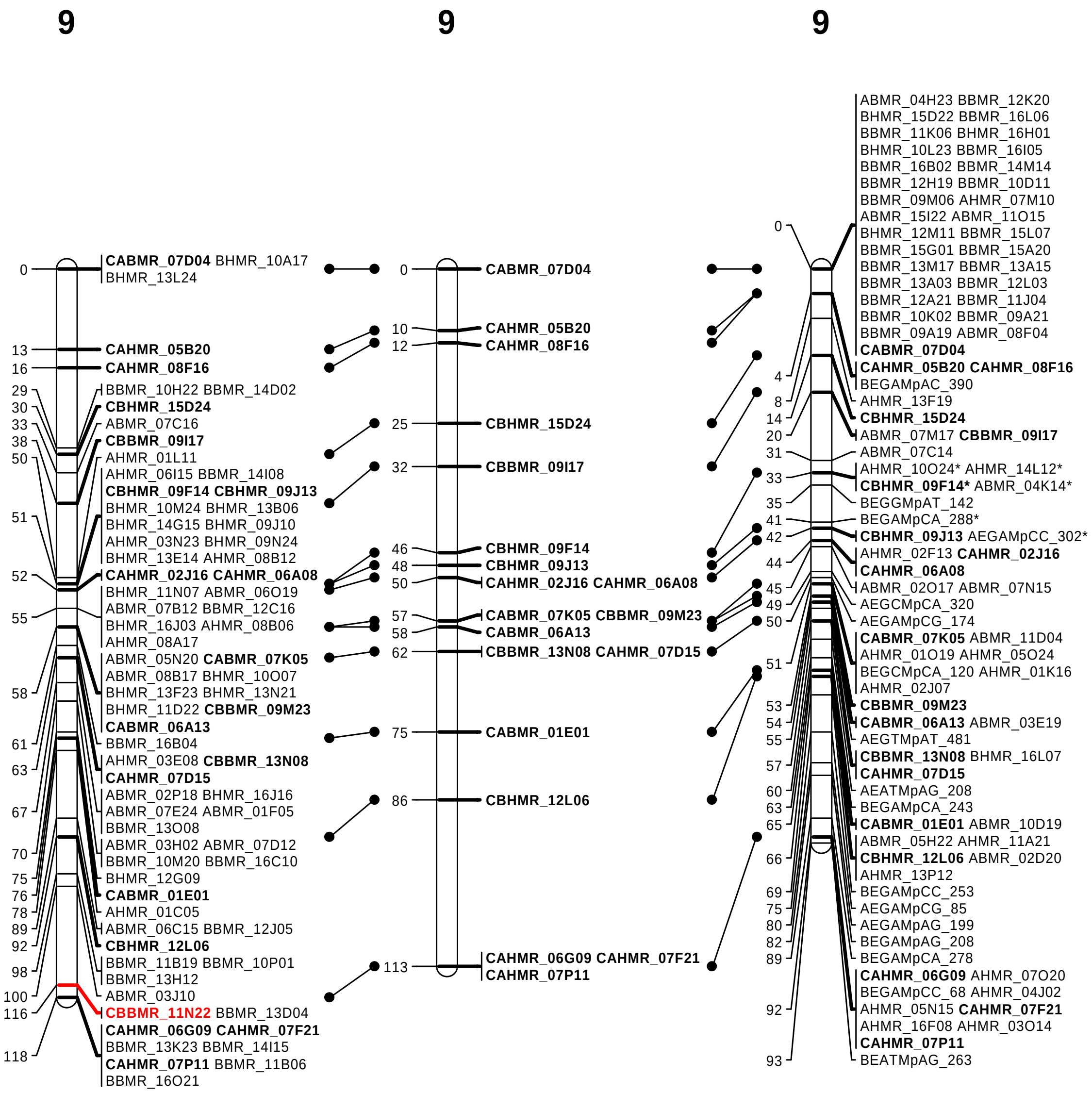
9
9
9
CABMR_07D04 BHMR_10A17
BHMR_13L24
CAHMR_05B20
CAHMR_08F16
CBHMR_15D24
CBBMR_09I17
CBHMR_09F14 CBHMR_09J13
CAHMR_02J16 CAHMR_06A08
CABMR_07K05
CBBMR_09M23
CABMR_06A13
CBBMR_13N08
CAHMR_07D15
CABMR_01E01
CBHMR_12L06
CBBMR_11N22 BBMR_13D04
CAHMR_06G09 CAHMR_07F21
CAHMR_07P11 BBMR_11B06
CABMR_07D04
CAHMR_05B20
CAHMR_08F16
CBHMR_15D24
CBBMR_09I17
CBHMR_09F14
CBHMR_09J13
CAHMR_02J16 CAHMR_06A08
CAHMR_07K05 CBBMR_09M23
CABMR_06A13
CBBMR_13N08 CAHMR_07D15
CABMR_01E01
CBHMR_12L06
CAHMR_06G09 CAHMR_07F21
CAHMR_07P11

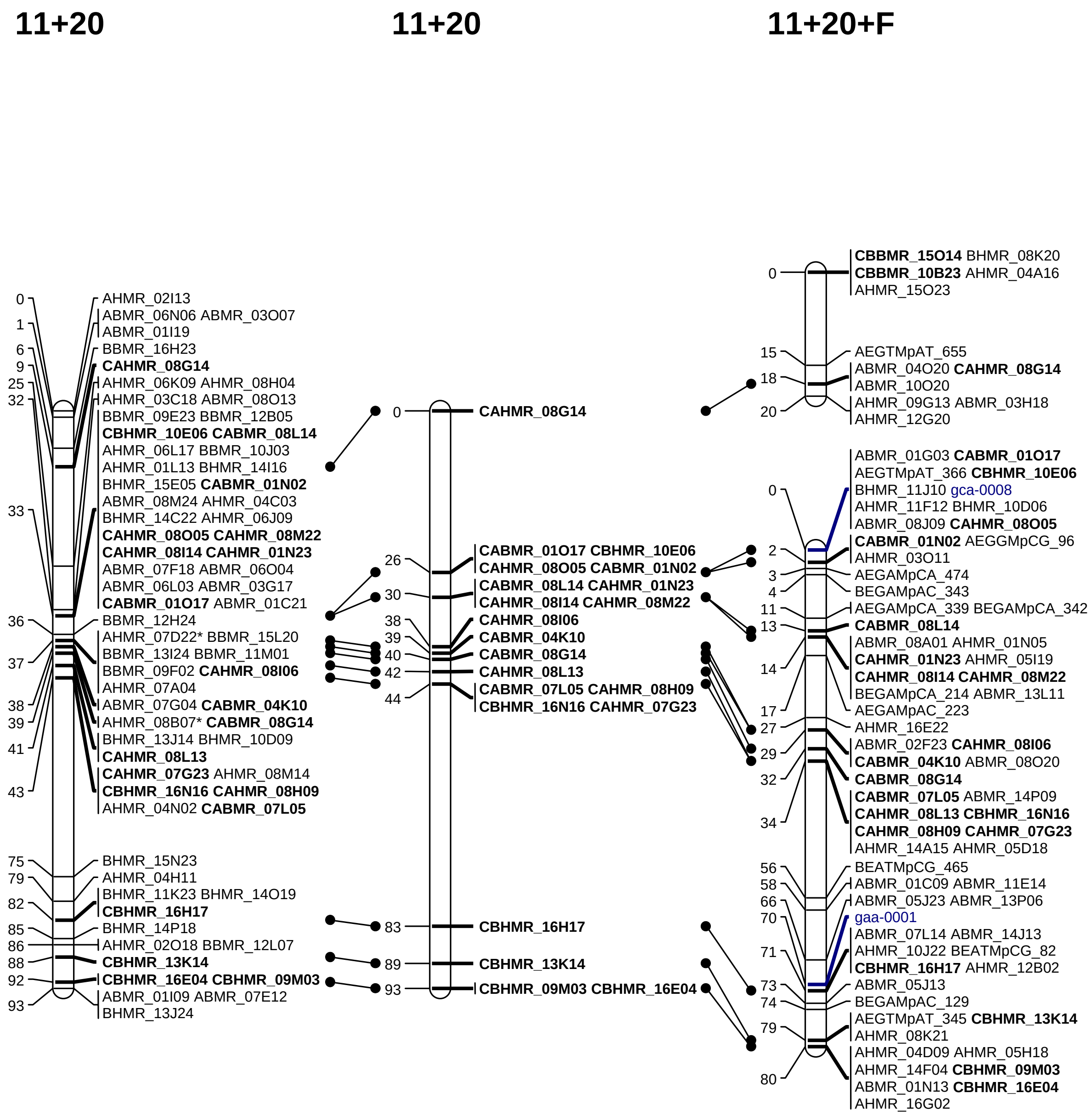
11+20
11+20
11+20+F
AHMR_02I13
ABMR_06N06 ABMR_03O07
ABMR_01I19
BBMR_16H23
CAHMR_08G14
AHMR_06K09 AHMR_08H04
AHMR_03C18 ABMR_08O13
BBMR_09E23 BBMR_12B05
CBHMR_10E06 CABMR_08L14
AHMR_06L17 BBMR_10J03
AHMR_01L13 BHMR_14I16
BHMR_15E05 CABMR_01N02
ABMR_08M24 AHMR_04C03
BHMR_14C22 AHMR_06J09
CAHMR_08O05 CAHMR_08M22
CAHMR_08I14 CAHMR_01N23
ABMR_07F18 ABMR_06O04
ABMR_06L03 ABMR_03G17
CABMR_01O17 ABMR_01C21
BBMR_12H24
AHMR_07D22* BBMR_15L20
BBMR_13I24 BBMR_11M01
BBMR_09F02 CAHMR_08I06
AHMR_07A04
ABMR_07G04 CABMR_04K10
AHMR_08B07* CABMR_08G14
BHMR_13J14 BHMR_10D09
CAHMR_08L13
CAHMR_07G23 AHMR_08M14
CBHMR_16N16 CAHMR_08H09
AHMR_04N02 CABMR_07L05
BHMR_15N23
AHMR_04H11
BHMR_11K23 BHMR_14O19
CBHMR_16H17
BHMR_14P18
AHMR_02O18 BBMR_12L07
CBHMR_13K14
CBHMR_16E04 CBHMR_09M03
ABMR_01I09 ABMR_07E12
BHMR_13J24
CAHMR_08G14
CABMR_01O17 CBHMR_10E06
CAHMR_08O05 CABMR_01N02
CABMR_08L14 CAHMR_01N23
CAHMR_08I14 CAHMR_08M22
CAHMR_08I06
CABMR_04K10
CABMR_08G14
CAHMR_08L13
CABMR_07L05 CAHMR_08H09
CBHMR_16N16 CAHMR_07G23
CBHMR_16H17
CBHMR_13K14
CBHMR_09M03 CBHMR_16E04
CBBMR_15O14 BHMR_08K20
CBBMR_10B23 AHMR_04A16
AHMR_15O23
AEGTMpAT_655
ABMR_04O20 CAHMR_08G14
ABMR_10O20
AHMR_09G13 ABMR_03H18
AHMR_12G20
ABMR_01G03 CABMR_01O17
AEGTMpAT_366 CBHMR_10E06
BHMR_11J10 gca-0008
AHMR_11F12 BHMR_10D06
ABMR_08J09 CAHMR_08O05
CABMR_01N02 AEGGMpCG_96
AHMR_03O11
AEGAMpCA_474
BEGAMpAC_343
AEGAMpCA_339 BEGAMpCA_342
CABMR_08L14
ABMR_08A01 AHMR_01N05
CAHMR_01N23 AHMR_05I19
CAHMR_08I14 CAHMR_08M22
BEGAMpCA_214 ABMR_13L11
AEGAMpAC_223
AHMR_16E22
ABMR_02F23 CAHMR_08I06
CABMR_04K10 ABMR_08O20
CABMR_08G14
CABMR_07L05 ABMR_14P09
CAHMR_08L13 CBHMR_16N16
CAHMR_08H09 CAHMR_07G23
AHMR_14A15 AHMR_05D18
BEATMpCG_465
ABMR_01C09 ABMR_11E14
ABMR_05J23 ABMR_13P06
gaa-0001
ABMR_07L14 ABMR_14J13
AHMR_10J22 BEATMpCG_82
CBHMR_16H17 AHMR_12B02
ABMR_05J13
BEGAMpAC_129
AEGTMpAT_345 CBHMR_13K14
AHMR_08K21
AHMR_04D09 AHMR_05H18
AHMR_14F04 CBHMR_09M03
ABMR_01N13 CBHMR_16E04
AHMR_16G02

Figure 5

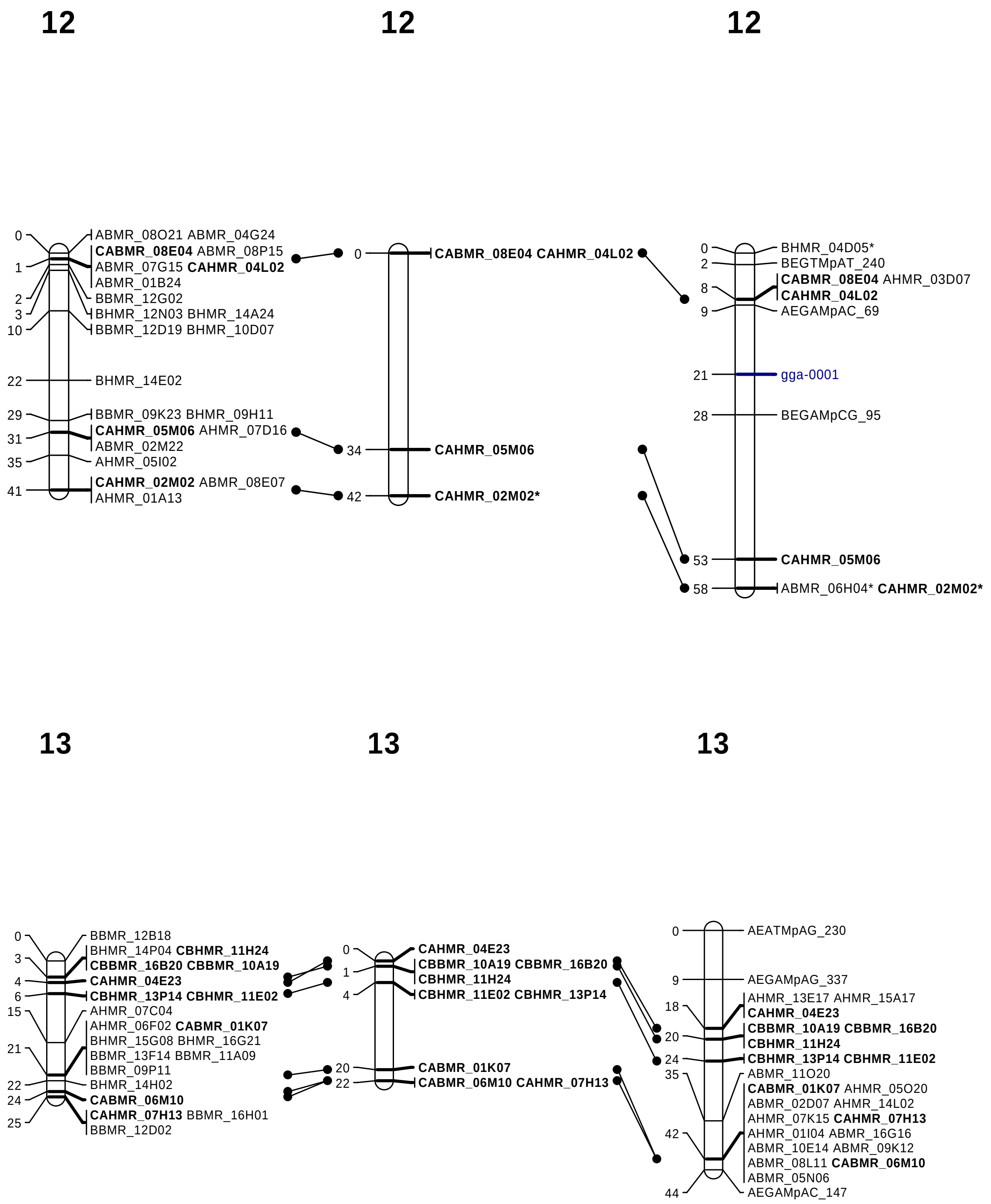

12
12
12
ABMR_08O21 ABMR_04G24
CABMR_08E04 ABMR_08P15
ABMR_07G15 CAHMR_04L02
ABMR_01B24
BBMR_12G02
BHMR_12N03 BHMR_14A24
BBMR_12D19 BHMR_10D07
BHMR_14E02
BBMR_09K23 BHMR_09H11
CAHMR_05M06 AHMR_07D16
ABMR_02M22
AHMR_05I02
CAHMR_02M02 ABMR_08E07
AHMR_01A13
CABMR_08E04 CAHMR_04L02
CAHMR_05M06
CAHMR_02M02*
BHMR_04D05*
BEGTMpAT_240
CABMR_08E04 AHMR_03D07
CAHMR_04L02
AEGAMpAC_69
gga-0001
BEGAMpCG_95
CAHMR_05M06
ABMR_06H04* CAHMR_02M02*
13
13
13
BBMR_12B18
BHMR_14P04 CBHMR_11H24
CBBMR_16B20 CBBMR_10A19
CAHMR_04E23
CBHMR_13P14 CBHMR_11E02
AHMR_07C04
AHMR_06F02 CABMR_01K07
BHMR_15G08 BHMR_16G21
BBMR_13F14 BBMR_11A09
BBMR_09P11
BHMR_14H02
CABMR_06M10
CAHMR_07H13 BBMR_16H01
BBMR_12D02
CAHMR_04E23
CBBMR_10A19 CBBMR_16B20
CBHMR_11H24
CBHMR_11E02 CBHMR_13P14
CABMR_01K07
CABMR_06M10 CAHMR_07H13
AEATMpAG_230
AEGAMpAG_337
AHMR_13E17 AHMR_15A17
CAHMR_04E23
CBBMR_10A19 CBBMR_16B20
CBHMR_11H24
CBHMR_13P14 CBHMR_11E02
ABMR_11O20
CABMR_01K07 AHMR_05O20
ABMR_02D07 AHMR_14L02
AHMR_07K15 CAHMR_07H13
AHMR_01I04 ABMR_16G16
ABMR_10E14 ABMR_09K12
ABMR_08L11 CABMR_06M10
ABMR_05N06
AEGAMpAC_147

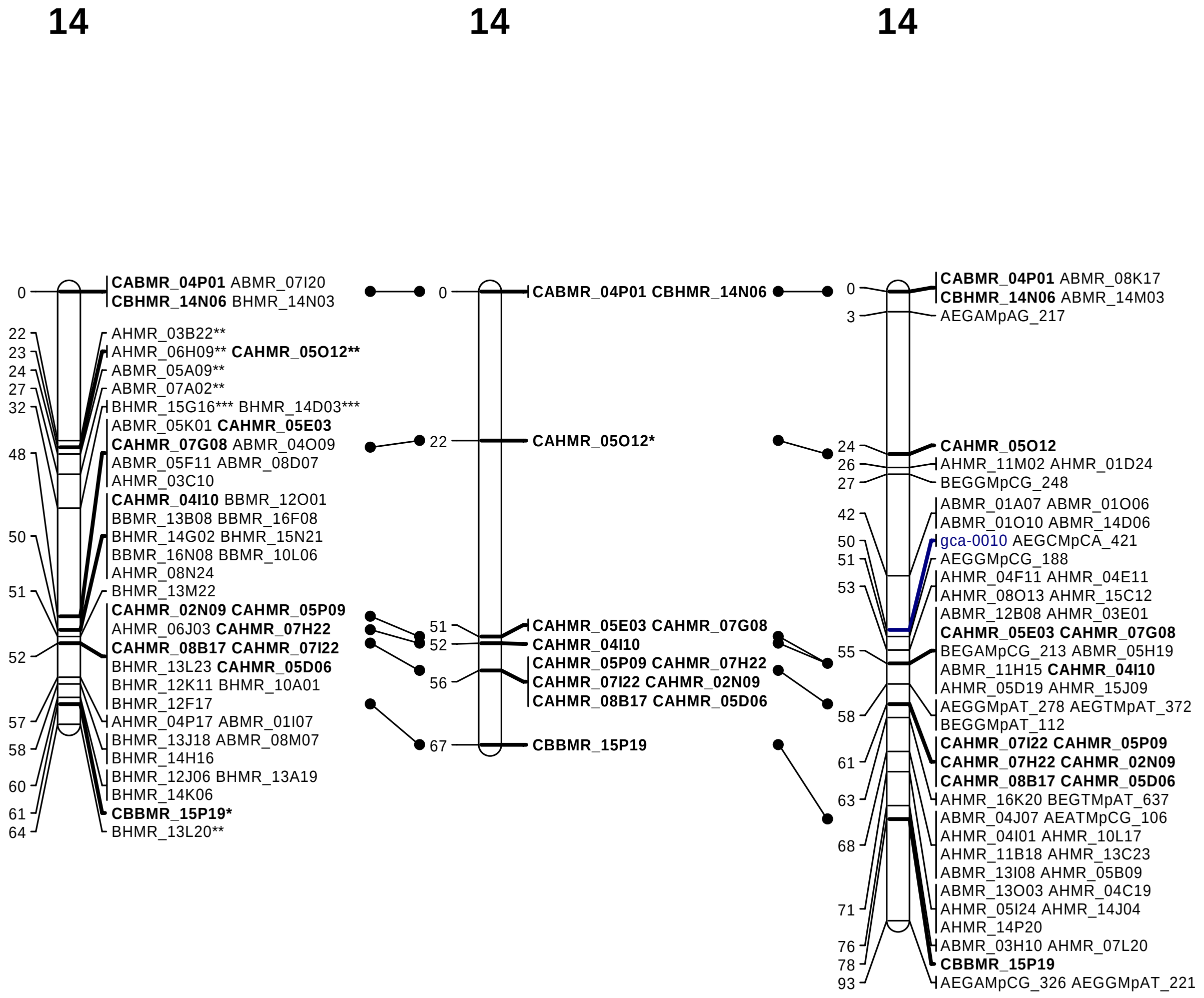
14
14
14
CABMR_04P01 ABMR_07I20
CBHMR_14N06 BHMR_14N03
AHMR_03B22**
AHMR_06H09** CAHMR_05O12**
ABMR_05A09**
ABMR_07A02**
BHMR_15G16*** BHMR_14D03***
ABMR_05K01 CAHMR_05E03
CAHMR_07G08 ABMR_04O09
ABMR_05F11 ABMR_08D07
AHMR_03C10
CAHMR_04I10 BBMR_12O01
BBMR_13B08 BBMR_16F08
BHMR_14G02 BHMR_15N21
BBMR_16N08 BBMR_10L06
AHMR_08N24
BHMR_13M22
CAHMR_02N09 CAHMR_05P09
AHMR_06J03 CAHMR_07H22
CAHMR_08B17 CAHMR_07I22
BHMR_13L23 CAHMR_05D06
BHMR_12K11 BHMR_10A01
BHMR_12F17
AHMR_04P17 ABMR_01I07
BHMR_13J18 ABMR_08M07
BHMR_14H16
BHMR_12J06 BHMR_13A19
BHMR_14K06
CBBMR_15P19*
BHMR_13L20**
CABMR_04P01 CBHMR_14N06
CAHMR_05O12*
CAHMR_05E03 CAHMR_07G08
CAHMR_04I10
CAHMR_05P09 CAHMR_07H22
CAHMR_07I22 CAHMR_02N09
CAHMR_08B17 CAHMR_05D06
CBBMR_15P19
CABMR_04P01 ABMR_08K17
CBHMR_14N06 ABMR_14M03
AEGAMpAG_217
CAHMR_05O12
AHMR_11M02 AHMR_01D24
BEGGMpCG_248
ABMR_01A07 ABMR_01O06
ABMR_01O10 ABMR_14D06
gca-0010 AEGCMpCA_421
AEGGMpCG_188
AHMR_04F11 AHMR_04E11
AHMR_08O13 AHMR_15C12
ABMR_12B08 AHMR_03E01
CAHMR_05E03 CAHMR_07G08
BEGAMpCG_213 ABMR_05H19
ABMR_11H15 CAHMR_04I10
AHMR_05D19 AHMR_15J09
AEGGMpAT_278 AEGTMpAT_372
BEGGMpAT_112
CAHMR_07I22 CAHMR_05P09
CAHMR_07H22 CAHMR_02N09
CAHMR_08B17 CAHMR_05D06
AHMR_16K20 BEGTMpAT_637
ABMR_04J07 AEATMpCG_106
AHMR_04I01 AHMR_10L17
AHMR_11B18 AHMR_13C23
ABMR_13I08 AHMR_05B09
ABMR_13O03 AHMR_04C19
AHMR_05I24 AHMR_14J04
AHMR_14P20
ABMR_03H10 AHMR_07L20
CBBMR_15P19
AEGAMpCG_326 AEGGMpAT_221

Figure 5

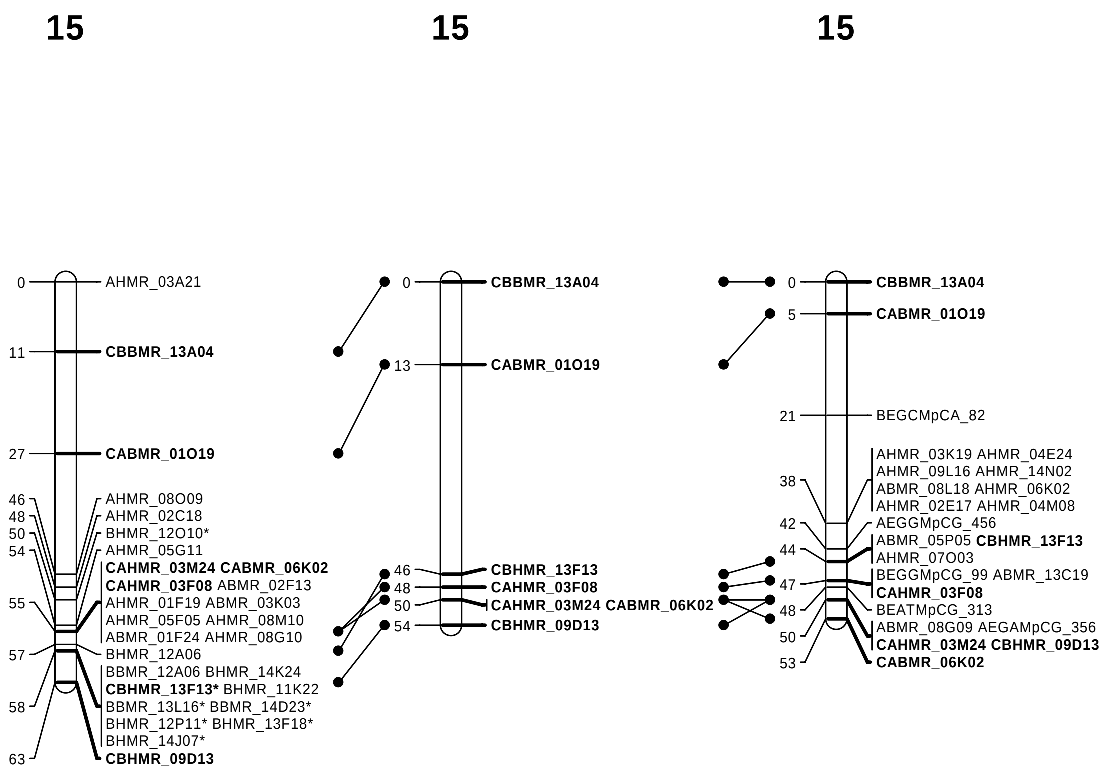
15
15
15
AHMR_03A21
CBBMR_13A04
CABMR_01O19
AHMR_08O09
AHMR_02C18
BHMR_12O10*
AHMR_05G11
CAHMR_03M24 CABMR_06K02
CAHMR_03F08 ABMR_02F13
AHMR_01F19 ABMR_03K03
AHMR_05F05 AHMR_08M10
ABMR_01F24 AHMR_08G10
BHMR_12A06
BBMR_12A06 BHMR_14K24
CBHMR_13F13* BHMR_11K22
BBMR_13L16* BBMR_14D23*
BHMR_12P11* BHMR_13F18*
BHMR_14J07*
CBHMR_09D13
CBBMR_13A04
CABMR_01O19
CBHMR_13F13
CAHMR_03F08
CAHMR_03M24 CABMR_06K02
CBHMR_09D13
CBBMR_13A04
CABMR_01O19
BEGCMpCA_82
AHMR_03K19 AHMR_04E24
AHMR_09L16 AHMR_14N02
ABMR_08L18 AHMR_06K02
AHMR_02E17 AHMR_04M08
AEGGMpCG_456
ABMR_05P05 CBHMR_13F13
AHMR_07O03
BEGGMpCG_99 ABMR_13C19
CAHMR_03F08
BEATMpCG_313
ABMR_08G09 AEGAMpCG_356
CAHMR_03M24 CBHMR_09D13
CABMR_06K02

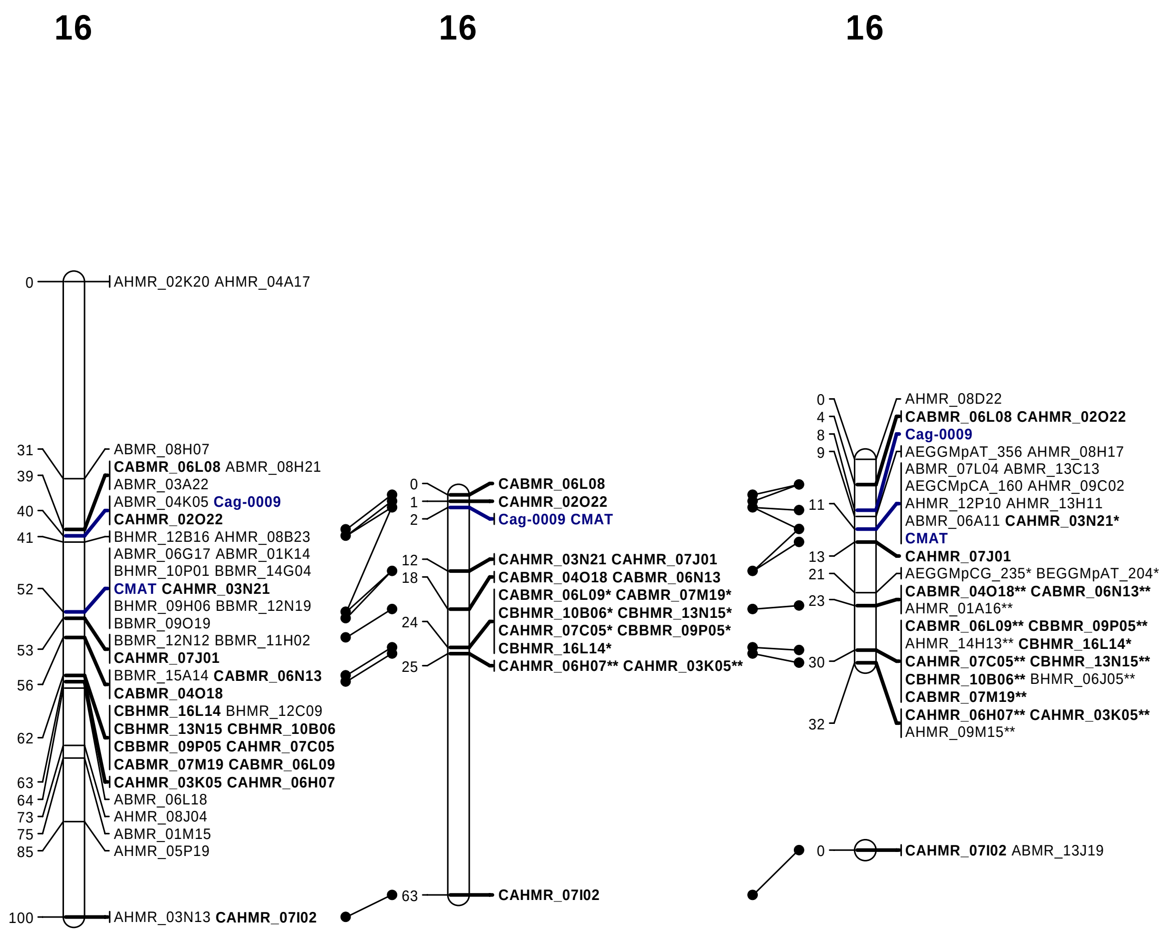
16
16
16
AHMR_02K20 AHMR_04A17
ABMR_08H07
CABMR_06L08 ABMR_08H21
ABMR_03A22
ABMR_04K05 Cag-0009
CAHMR_02O22
BHMR_12B16 AHMR_08B23
ABMR_06G17 ABMR_01K14
BHMR_10P01 BBMR_14G04
CMAT CAHMR_03N21
BHMR_09H06 BBMR_12N19
BBMR_09O19
BBMR_12N12 BBMR_11H02
CAHMR_07J01
BBMR_15A14 CABMR_06N13
CABMR_04O18
CBHMR_16L14 BHMR_12C09
CBHMR_13N15 CBHMR_10B06
CBBMR_09P05 CAHMR_07C05
CABMR_07M19 CABMR_06L09
CAHMR_03K05 CAHMR_06H07
ABMR_06L18
AHMR_08J04
ABMR_01M15
AHMR_05P19
AHMR_03N13 CAHMR_07I02
CABMR_06L08
CAHMR_02O22
Cag-0009 CMAT
CAHMR_03N21 CAHMR_07J01
CABMR_04O18 CABMR_06N13
CABMR_06L09* CABMR_07M19*
CBHMR_10B06* CBHMR_13N15*
CAHMR_07C05* CBBMR_09P05*
CBHMR_16L14*
CAHMR_06H07** CAHMR_03K05**
CAHMR_07I02
AHMR_08D22
CABMR_06L08 CAHMR_02O22
Cag-0009
AEGGMpAT_356 AHMR_08H17
ABMR_07L04 ABMR_13C13
AEGCMpCA_160 AHMR_09C02
AHMR_12P10 AHMR_13H11
ABMR_06A11 CAHMR_03N21*
CMAT
CAHMR_07J01
AEGGMpCG_235* BEGGMpAT_204*
CABMR_04O18** CABMR_06N13**
AHMR_01A16**
CABMR_06L09** CBBMR_09P05**
AHMR_14H13** CBHMR_16L14*
CAHMR_07C05** CBHMR_13N15**
CBHMR_10B06** BHMR_06J05**
CABMR_07M19**
CAHMR_06H07** CAHMR_03K05**
AHMR_09M15**
CAHMR_07I02 ABMR_13J19

Figure 5

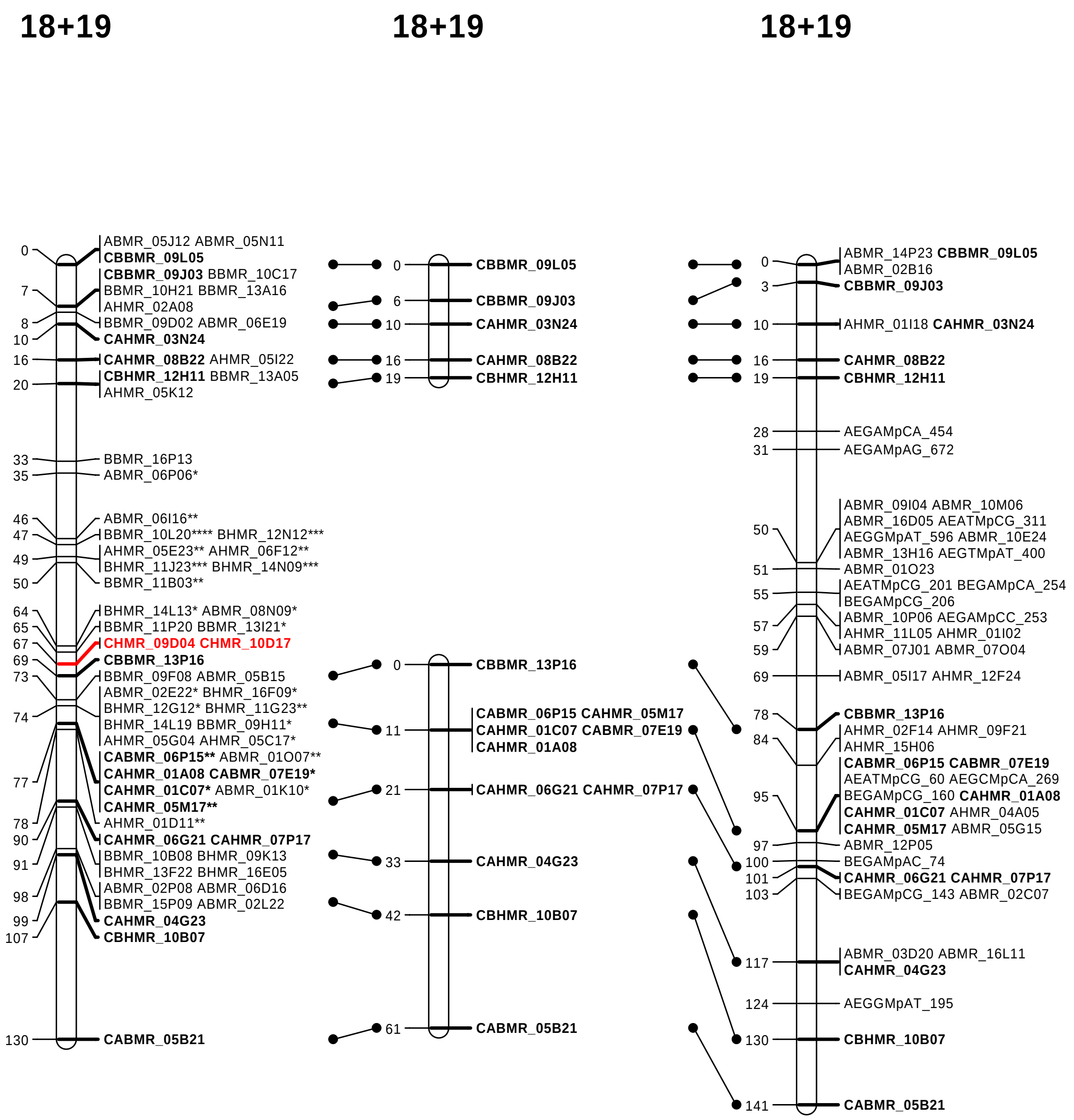
18+19
18+19
18+19
ABMR_05J12 ABMR_05N11
CBBMR_09L05
CBBMR_09J03 BBMR_10C17
BBMR_10H21 BBMR_13A16
AHMR_02A08
BBMR_09D02 ABMR_06E19
CAHMR_03N24
CAHMR_08B22 AHMR_05I22
CBHMR_12H11 BBMR_13A05
AHMR_05K12
BBMR_16P13
ABMR_06P06*
ABMR_06I16**
BBMR_10L20**** BHMR_12N12***
AHMR_05E23** AHMR_06F12**
BHMR_11J23*** BHMR_14N09***
BBMR_11B03**
BHMR_14L13* ABMR_08N09*
BBMR_11P20 BBMR_13I21*
CHMR_09D04 CHMR_10D17
CBBMR_13P16
BBMR_09F08 ABMR_05B15
ABMR_02E22* BHMR_16F09*
BHMR_12G12* BHMR_11G23**
BHMR_14L19 BBMR_09H11*
AHMR_05G04 AHMR_05C17*
CABMR_06P15** ABMR_01O07**
CAHMR_01A08 CABMR_07E19*
CAHMR_01C07* ABMR_01K10*
CAHMR_05M17**
AHMR_01D11**
CAHMR_06G21 CAHMR_07P17
BBMR_10B08 BHMR_09K13
BHMR_13F22 BHMR_16E05
ABMR_02P08 ABMR_06D16
BBMR_15P09 ABMR_02L22
CAHMR_04G23
CBHMR_10B07
CABMR_05B21
CBBMR_09L05
CBBMR_09J03
CAHMR_03N24
CAHMR_08B22
CBHMR_12H11
CBBMR_13P16
CABMR_06P15 CAHMR_05M17
CAHMR_01C07 CABMR_07E19
CAHMR_01A08
CAHMR_06G21 CAHMR_07P17
CAHMR_04G23
CBHMR_10B07
CABMR_05B21
ABMR_14P23 CBBMR_09L05
ABMR_02B16
CBBMR_09J03
AHMR_01I18 CAHMR_03N24
CAHMR_08B22
CBHMR_12H11
AEGAMpCA_454
AEGAMpAG_672
ABMR_09I04 ABMR_10M06
ABMR_16D05 AEATMpCG_311
AEGGMpAT_596 ABMR_10E24
ABMR_13H16 AEGTMpAT_400
ABMR_01O23
AEATMpCG_201 BEGAMpCA_254
BEGAMpCG_206
ABMR_10P06 AEGAMpCC_253
AHMR_11L05 AHMR_01I02
ABMR_07J01 ABMR_07O04
ABMR_05I17 AHMR_12F24
CBBMR_13P16
AHMR_02F14 AHMR_09F21
AHMR_15H06
CABMR_06P15 CABMR_07E19
AEATMpCG_60 AEGCMpCA_269
BEGAMpCG_160 CAHMR_01A08
CAHMR_01C07 AHMR_04A05
CAHMR_05M17 ABMR_05G15
ABMR_12P05
BEGAMpAC_74
CAHMR_06G21 CAHMR_07P17
BEGAMpCG_143 ABMR_02C07
ABMR_03D20 ABMR_16L11
CAHMR_04G23
AEGGMpAT_195
CBHMR_10B07
CABMR_05B21

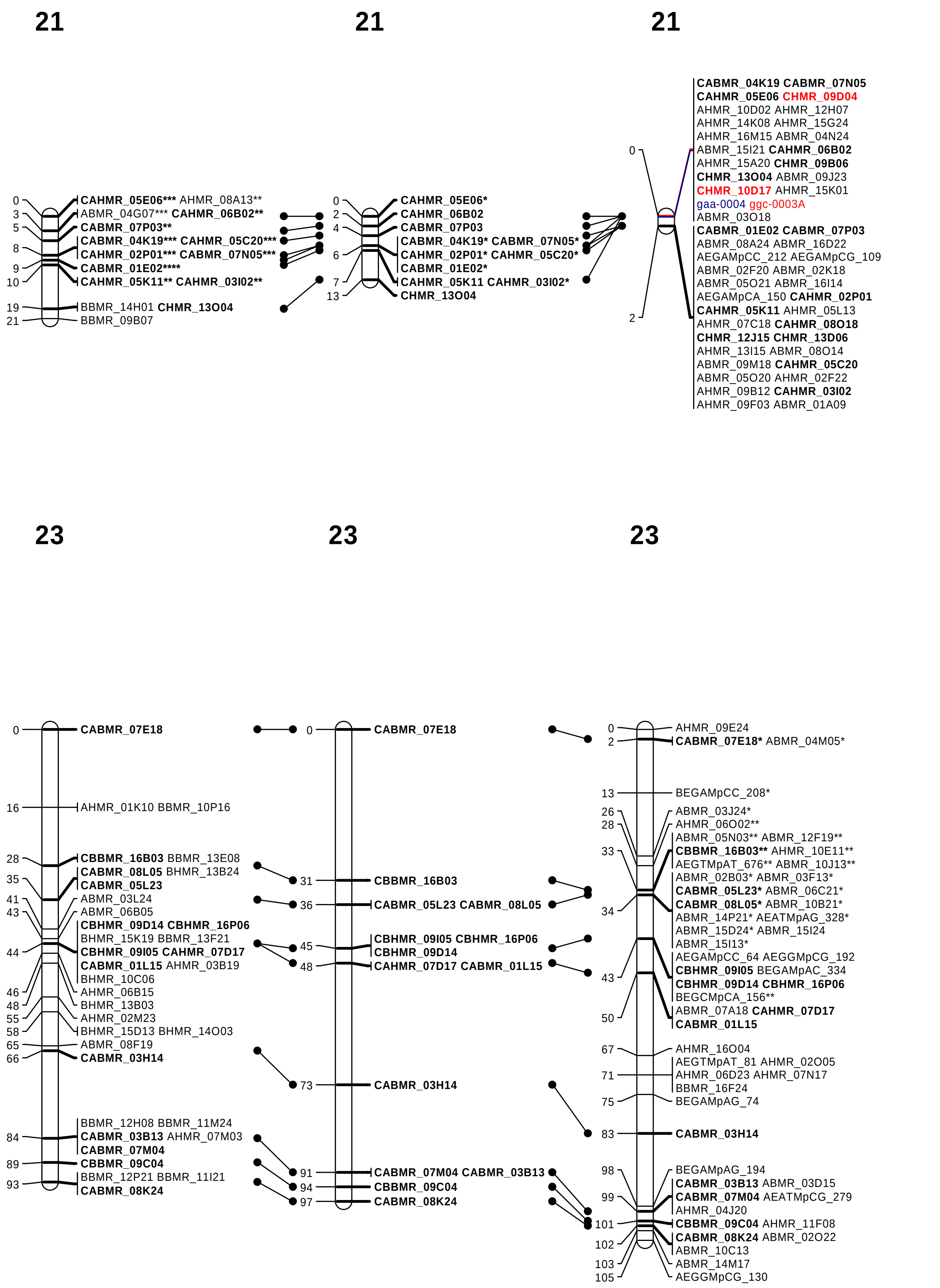
21
21
21
CAHMR_05E06*** AHMR_08A13**
ABMR_04G07*** CAHMR_06B02**
CAHMR_07P03**
CABMR_04K19*** CAHMR_05C20***
CAHMR_02P01*** CABMR_07N05***
CABMR_01E02****
CAHMR_05K11** CAHMR_03I02**
BBMR_14H01 CHMR_13O04
BBMR_09B07
CAHMR_05E06*
CAHMR_06B02
CABMR_07P03
CABMR_04K19* CABMR_07N05*
CAHMR_02P01* CAHMR_05C20*
CABMR_01E02*
CAHMR_05K11 CAHMR_03I02*
CHMR_13O04
CABMR_04K19 CABMR_07N05
CAHMR_05E06 CHMR_09D04
AHMR_10D02 AHMR_12H07
AHMR_14K08 AHMR_15G24
AHMR_16M15 ABMR_04N24
ABMR_15I21 CAHMR_06B02
AHMR_15A20 CHMR_09B06
CHMR_13O04 ABMR_09J23
CHMR_10D17 AHMR_15K01
gaa-0004 ggc-0003A
ABMR_03O18
CABMR_01E02 CABMR_07P03
ABMR_08A24 ABMR_16D22
AEGAMpCC_212 AEGAMpCG_109
ABMR_02F20 ABMR_02K18
ABMR_05O21 ABMR_16I14
AEGAMpCA_150 CAHMR_02P01
CAHMR_05K11 AHMR_05L13
AHMR_07C18 CAHMR_08O18
CHMR_12J15 CHMR_13D06
AHMR_13I15 ABMR_08O14
ABMR_09M18 CAHMR_05C20
ABMR_05O20 AHMR_02F22
AHMR_09B12 CAHMR_03I02
AHMR_09F03 ABMR_01A09
23
23
23
CABMR_07E18
AHMR_01K10 BBMR_10P16
CBBMR_16B03 BBMR_13E08
CABMR_08L05 BHMR_13B24
CABMR_05L23
ABMR_03L24
ABMR_06B05
CBHMR_09D14 CBHMR_16P06
BHMR_15K19 BBMR_13F21
CBHMR_09I05 CAHMR_07D17
CABMR_01L15 AHMR_03B19
BHMR_10C06
AHMR_06B15
BHMR_13B03
AHMR_02M23
BHMR_15D13 BHMR_14O03
ABMR_08F19
CABMR_03H14
BBMR_12H08 BBMR_11M24
CABMR_03B13 AHMR_07M03
CABMR_07M04
CBBMR_09C04
BBMR_12P21 BBMR_11I21
CABMR_08K24
CABMR_07E18
CBBMR_16B03
CABMR_05L23 CABMR_08L05
CBHMR_09I05 CBHMR_16P06
CBHMR_09D14
CAHMR_07D17 CABMR_01L15
CABMR_03H14
CABMR_07M04 CABMR_03B13
CBBMR_09C04
CABMR_08K24
AHMR_09E24
CABMR_07E18* ABMR_04M05*
BEGAMpCC_208*
ABMR_03J24*
AHMR_06O02**
ABMR_05N03** ABMR_12F19**
CBBMR_16B03** AHMR_10E11**
AEGTMpAT_676** ABMR_10J13**
ABMR_02B03* ABMR_03F13*
CABMR_05L23* ABMR_06C21*
CABMR_08L05* ABMR_10B21*
ABMR_14P21* AEATMpAG_328*
ABMR_15D24* ABMR_15I24
ABMR_15I13*
AEGAMpCC_64 AEGGMpCG_192
CBHMR_09I05 BEGAMpAC_334
CBHMR_09D14 CBHMR_16P06
BEGCMpCA_156**
ABMR_07A18 CAHMR_07D17
CABMR_01L15
AHMR_16O04
AEGTMpAT_81 AHMR_02O05
AHMR_06D23 AHMR_07N17
BBMR_16F24
BEGAMpAG_74
CABMR_03H14
BEGAMpAG_194
CABMR_03B13 ABMR_03D15
CABMR_07M04 AEATMpCG_279
AHMR_04J20
CBBMR_09C04 AHMR_11F08
CABMR_08K24 ABMR_02O22
ABMR_10C13
ABMR_14M17
AEGGMpCG_130

Figure 5

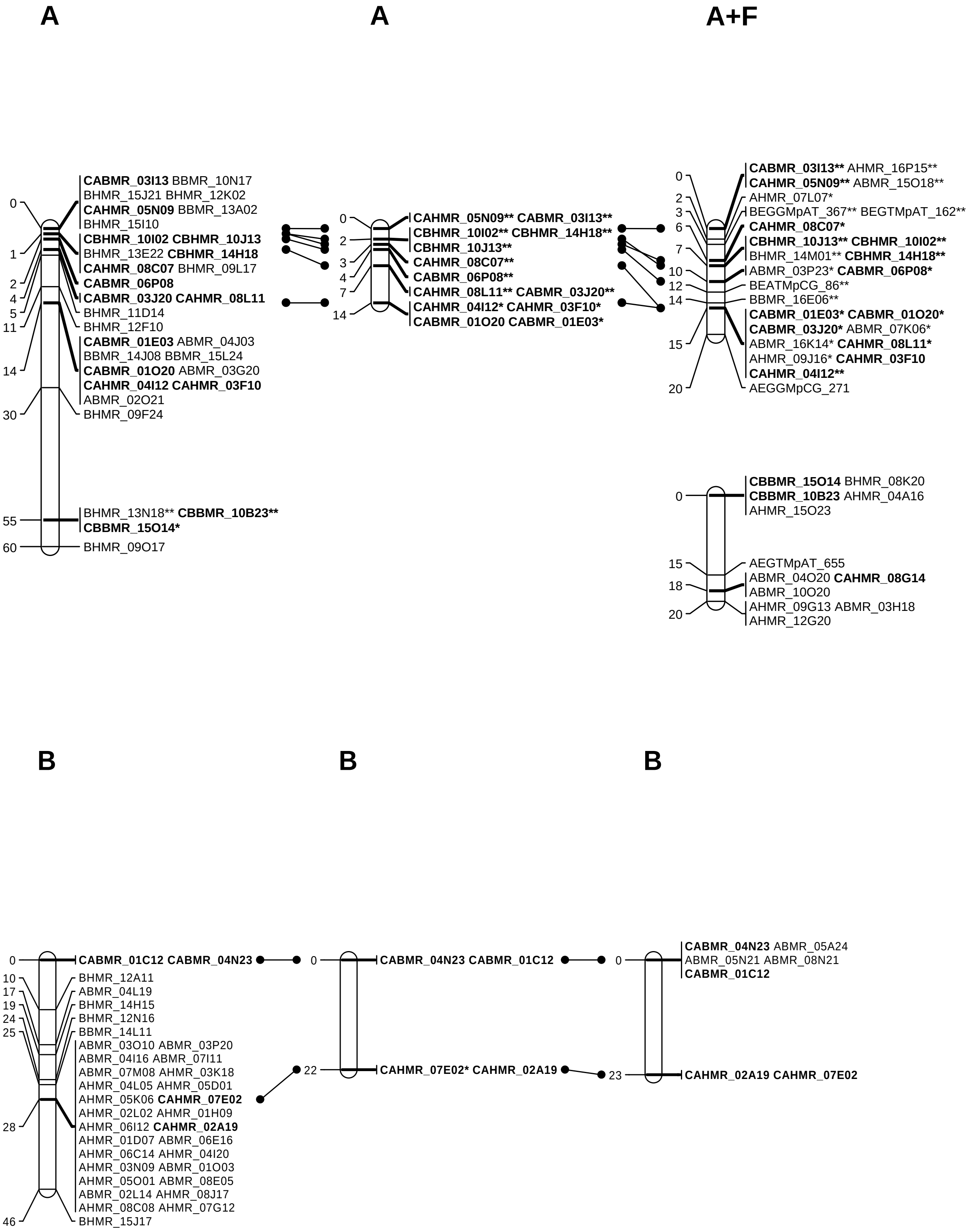
A
A
A+F
B
B
B
CABMR_03I13 BBMR_10N17
BHMR_15J21 BHMR_12K02
CAHMR_05N09 BBMR_13A02
BHMR_15I10
CBHMR_10I02 CBHMR_10J13
BHMR_13E22 CBHMR_14H18
CAHMR_08C07 BHMR_09L17
CABMR_06P08
CABMR_03J20 CAHMR_08L11
BHMR_11D14
BHMR_12F10
CABMR_01E03 ABMR_04J03
BBMR_14J08 BBMR_15L24
CABMR_01O20 ABMR_03G20
CAHMR_04I12 CAHMR_03F10
ABMR_02O21
BHMR_09F24
BHMR_13N18** CBBMR_10B23**
CBBMR_15O14*
BHMR_09O17
0 1 2 4 5 11 14 30 55 60
CAHMR_05N09** CABMR_03I13**
CBHMR_10I02** CBHMR_14H18**
CBHMR_10J13**
CAHMR_08C07**
CABMR_06P08**
CAHMR_08L11** CABMR_03J20**
CAHMR_04I12* CAHMR_03F10*
CABMR_01O20 CABMR_01E03*
0 2 3 4 7 14
CABMR_03I13** AHMR_16P15**
CAHMR_05N09** ABMR_15O18**
AHMR_07L07*
BEGGMpAT_367** BEGTMpAT_162**
CAHMR_08C07*
CBHMR_10J13** CBHMR_10I02**
BHMR_14M01** CBHMR_14H18**
ABMR_03P23* CABMR_06P08*
BEATMpCG_86**
BBMR_16E06**
CABMR_01E03* CABMR_01O20*
CABMR_03J20* ABMR_07K06*
ABMR_16K14* CAHMR_08L11*
AHMR_09J16* CAHMR_03F10
CAHMR_04I12**
AEGGMpCG_271
0 2 3 6 7 10 12 14 15 20
CBBMR_15O14 BHMR_08K20
CBBMR_10B23 AHMR_04A16
AHMR_15O23
AEGTMpAT_655
ABMR_04O20 CAHMR_08G14
ABMR_10O20
AHMR_09G13 ABMR_03H18
AHMR_12G20
0 15 18 20
CABMR_01C12 CABMR_04N23
BHMR_12A11
ABMR_04L19
BHMR_14H15
BHMR_12N16
BBMR_14L11
ABMR_03O10 ABMR_03P20
ABMR_04I16 ABMR_07I11
ABMR_07M08 AHMR_03K18
AHMR_04L05 AHMR_05D01
AHMR_05K06 CAHMR_07E02
AHMR_02L02 AHMR_01H09
AHMR_06I12 CAHMR_02A19
AHMR_01D07 ABMR_06E16
AHMR_06C14 AHMR_04I20
AHMR_03N09 ABMR_01O03
AHMR_05O01 ABMR_08E05
ABMR_02L14 AHMR_08J17
AHMR_08C08 AHMR_07G12
BHMR_15J17
0 10 17 19 24 25 28 46
CABMR_04N23 CABMR_01C12
CAHMR_07E02* CAHMR_02A19
0 22
CABMR_04N23 ABMR_05A24
ABMR_05N21 ABMR_08N21
CABMR_01C12
CABMR_02A19 CAHMR_07E02
0 23

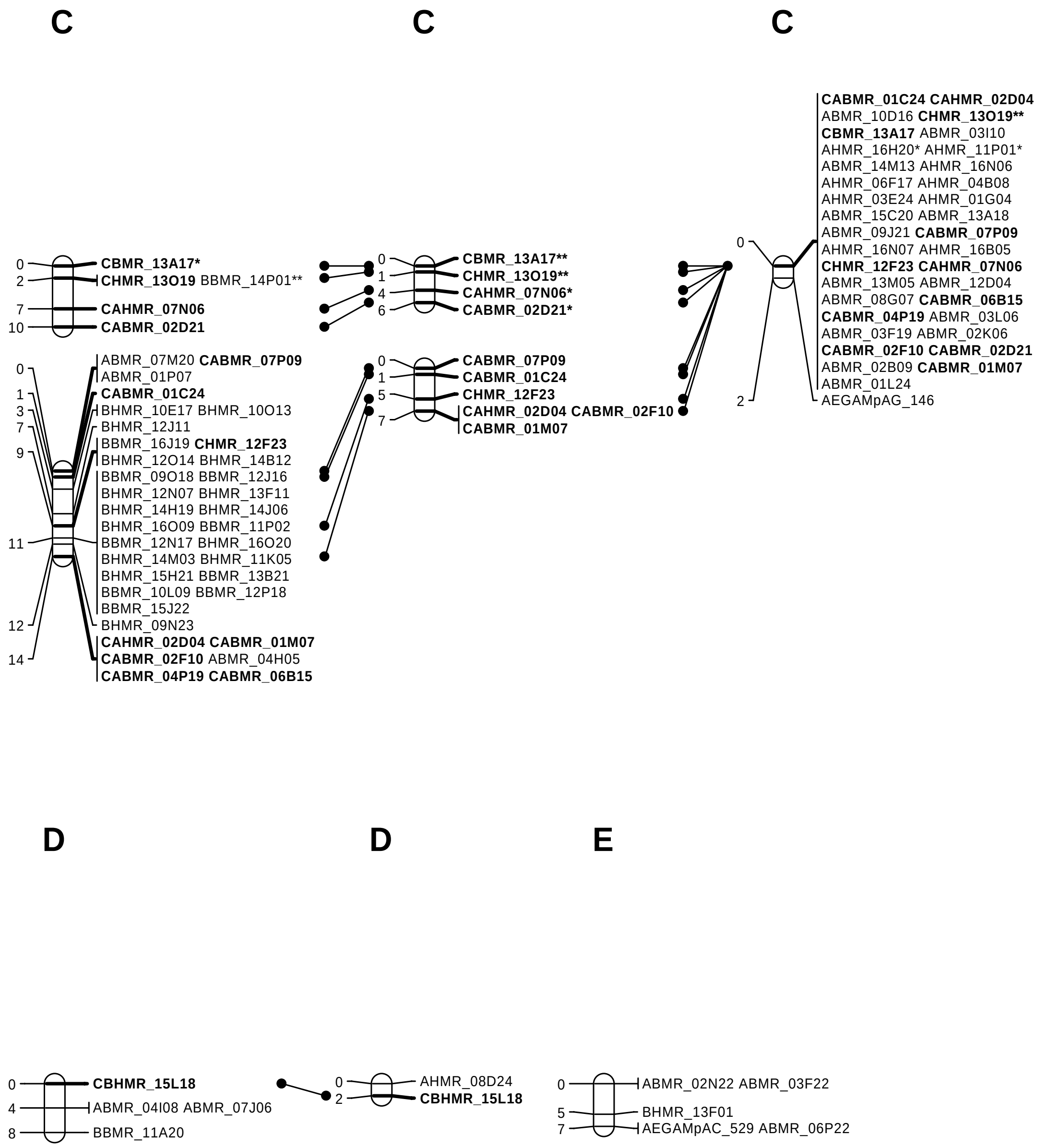
C
C
C
0 CBMR_13A17*
2 CHMR_13O19 BBMR_14P01**
7 CAHMR_07N06
10 CABMR_02D21
0 ABMR_07M20 CABMR_07P09
ABMR_01P07
1 CABMR_01C24
3 BHMR_10E17 BHMR_10O13
7 BHMR_12J11
9 BBMR_16J19 CHMR_12F23
BHMR_12O14 BHMR_14B12
BBMR_09O18 BBMR_12J16
BHMR_12N07 BHMR_13F11
BHMR_14H19 BHMR_14J06
BHMR_16O09 BBMR_11P02
11 BBMR_12N17 BHMR_16O20
BHMR_14M03 BHMR_11K05
BHMR_15H21 BBMR_13B21
BBMR_10L09 BBMR_12P18
BBMR_15J22
12 BHMR_09N23
14 CAHMR_02D04 CABMR_01M07
CABMR_02F10 ABMR_04H05
CABMR_04P19 CABMR_06B15
0 CBMR_13A17**
1 CHMR_13O19**
4 CAHMR_07N06*
6 CABMR_02D21*
0 CABMR_07P09
1 CABMR_01C24
5 CHMR_12F23
7 CAHMR_02D04 CABMR_02F10
CABMR_01M07
CABMR_01C24 CAHMR_02D04
ABMR_10D16 CHMR_13O19**
CBMR_13A17 ABMR_03I10
AHMR_16H20* AHMR_11P01*
ABMR_14M13 AHMR_16N06
AHMR_06F17 AHMR_04B08
AHMR_03E24 AHMR_01G04
ABMR_15C20 ABMR_13A18
0 ABMR_09J21 CABMR_07P09
AHMR_16N07 AHMR_16B05
CHMR_12F23 CAHMR_07N06
ABMR_13M05 ABMR_12D04
ABMR_08G07 CABMR_06B15
CABMR_04P19 ABMR_03L06
ABMR_03F19 ABMR_02K06
CABMR_02F10 CABMR_02D21
ABMR_02B09 CABMR_01M07
ABMR_01L24
2 AEGAMpAG_146
D
D
E
0 CBHMR_15L18
4 ABMR_04I08 ABMR_07J06
8 BBMR_11A20
0 AHMR_08D24
2 CBHMR_15L18
0 ABMR_02N22 ABMR_03F22
5 BHMR_13F01
7 AEGAMpAC_529 ABMR_06P22
