# Supplementary material for: Meiosis Drives Extraordinary Genome Plasticity in the Haploid Fungal Plant Pathogen Mycosphaerella graminicola
Source: PLoS One. 2009 Jun 10;4(6):e5863. doi: 10.1371/journal.pone.0005863 (PMC2689623; doi:10.1371/journal.pone.0005863)
Supplement: Figure S2 — Alignment of linkage group 21 between the IPO323×IPO95052 cross (left) and the IPO323×IPO94269 cross (right) shows recombination in the former but not in the latter. This indicates absence of this linkage group in isolate IPO94269. For IPO323×IPO94269, only markers from IPO323 could be mapped on this linkage group, and no markers from IPO94269, confirming that IPO94269 lacks this linkage group. Lines are drawn between markers that segregated in both populations. Stars next to the markers for the IPO323×IPO94269 cross indicate segregation distortion of the markers; * (P<0.05), ** (P<0.01), *** (P<0.005) or **** (P<0.001). (0.03 MB PDF) [file pone.0005863.s003.pdf]

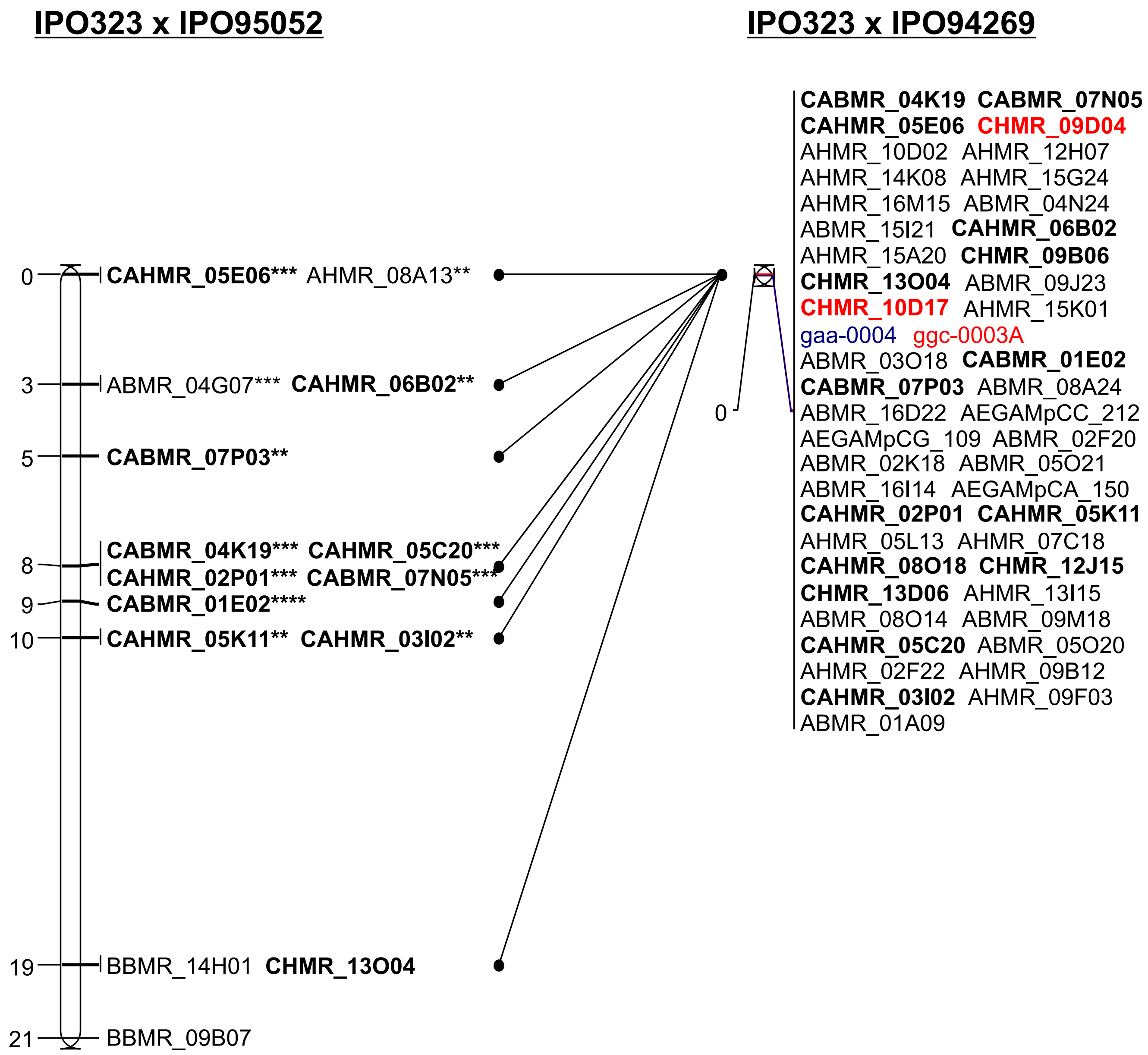
IPO323 x IPO95052
IPO323 x IPO94269
0
CAHMR_05E06*** AHMR_08A13**
3
ABMR_04G07*** CAHMR_06B02**
5
CABMR_07P03**
8
CABMR_04K19*** CAHMR_05C20***
CAHMR_02P01*** CABMR_07N05***
9
CABMR_01E02****
10
CAHMR_05K11** CAHMR_03I02**
19
BBMR_14H01 CHMR_13O04
21
BBMR_09B07
0
CABMR_04K19 CABMR_07N05
CAHMR_05E06 CHMR_09D04
AHMR_10D02 AHMR_12H07
AHMR_14K08 AHMR_15G24
AHMR_16M15 ABMR_04N24
ABMR_15I21 CAHMR_06B02
AHMR_15A20 CHMR_09B06
CHMR_13O04 ABMR_09J23
CHMR_10D17 AHMR_15K01
gaa-0004 ggc-0003A
ABMR_03O18 CABMR_01E02
CABMR_07P03 ABMR_08A24
ABMR_16D22 AEGAMpCC_212
AEGAMpCG_109 ABMR_02F20
ABMR_02K18 ABMR_05O21
ABMR_16I14 AEGAMpCA_150
CAHMR_02P01 CAHMR_05K11
AHMR_05L13 AHMR_07C18
CAHMR_08O18 CHMR_12J15
CHMR_13D06 AHMR_13I15
ABMR_08O14 ABMR_09M18
CAHMR_05C20 ABMR_05O20
AHMR_02F22 AHMR_09B12
CAHMR_03I02 AHMR_09F03
ABMR_01A09
