# Supplementary material for: Meiosis Drives Extraordinary Genome Plasticity in the Haploid Fungal Plant Pathogen Mycosphaerella graminicola
Source: PLoS One. 2009 Jun 10;4(6):e5863. doi: 10.1371/journal.pone.0005863 (PMC2689623; doi:10.1371/journal.pone.0005863)
Supplement: Figure S3 — Confirmation of chromosome loss by PCR amplification. A. Confirmation of loss of LG 8 and LG 12 by SSR amplification. Loci ac-0007 (LG 8) and gga-0001 (LG 12) confirm that these linkage groups are absent in the underlined progeny isolates from the crosses IPO323×IPO94269 and IPO323×IPO95052 as neither of the parental alleles are amplified. Isolates 1158 and 1179 are positive controls and SSR ag-0003 (LG 2) is a positive PCR control in all duplex reactions. B. Confirmation of loss of LGs 13, 15, A and C by PCR with primers developed from DArT marker sequence data in the underlined progeny isolates derived from crosses between M. graminicola IPO323×IPO94269 and IPO323×IPO95052. Isolates 1158 and 1179 are positive control isolates, except in LGs C and 13 that have isolates 1158/2026 and 2032/2033, respectively, as positive checks. For LG 15* the CABMR_07D07 DArT fragment (129 bp) was used as a positive PCR control, while for the other linkage groups DArT fragment AHMR_08O09 (728 bp) was used. C. Confirmation of loss of LG 8 by PCR with primers developed from DArT marker sequence data in underlined progeny isolates derived from crosses between M. graminicola IPO323×IPO94269 and IPO323×IPO95052. This figure is composed of eight panels that are individually divided by a central marker lane. The left part of each panel represents the three parental isolates of the mapping populations (IPO323, IPO94269 and IPO95052), two positive control isolates (1158/1179), and seven progeny isolates that lack LG 8. The right part of each panel links to Fig. 2D and represents the two parental isolates (IPO323 and IPO95052), two twin isolates (1103/1126), two mirror isolates (1128/1183) and two twin isolates that lack LG 8 (2137/2139). In all panels DArT fragment AHMR_08O09 is the positive control (top band in each panel, 728 bp, located on LG 15). (1.60 MB PDF) [file pone.0005863.s004.pdf]

A

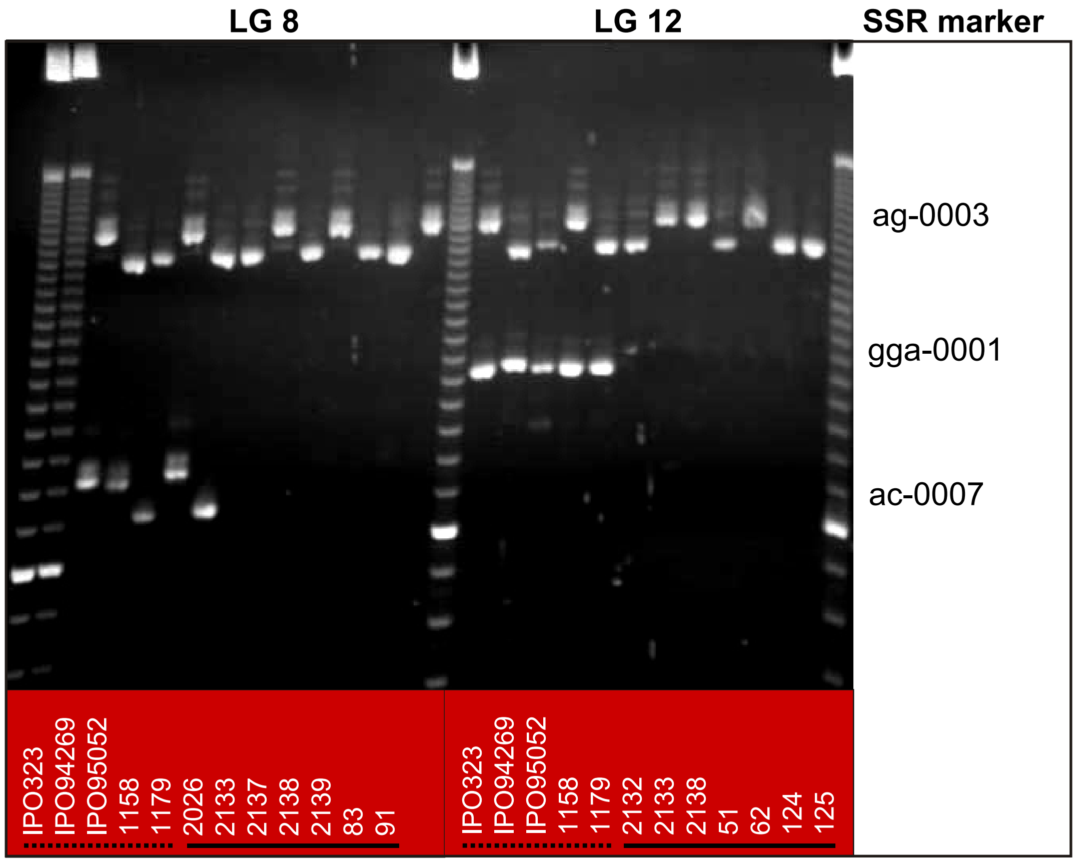
LG 8
LG 12
SSR marker
ag-0003
gga-0001
ac-0007
IPO323
IPO94269
IPO95052
1158
1179
2026
2133
2137
2138
2139
83
91
IPO323
IPO94269
IPO95052
1158
1179
2132
2133
2138
51
62
124
125

B

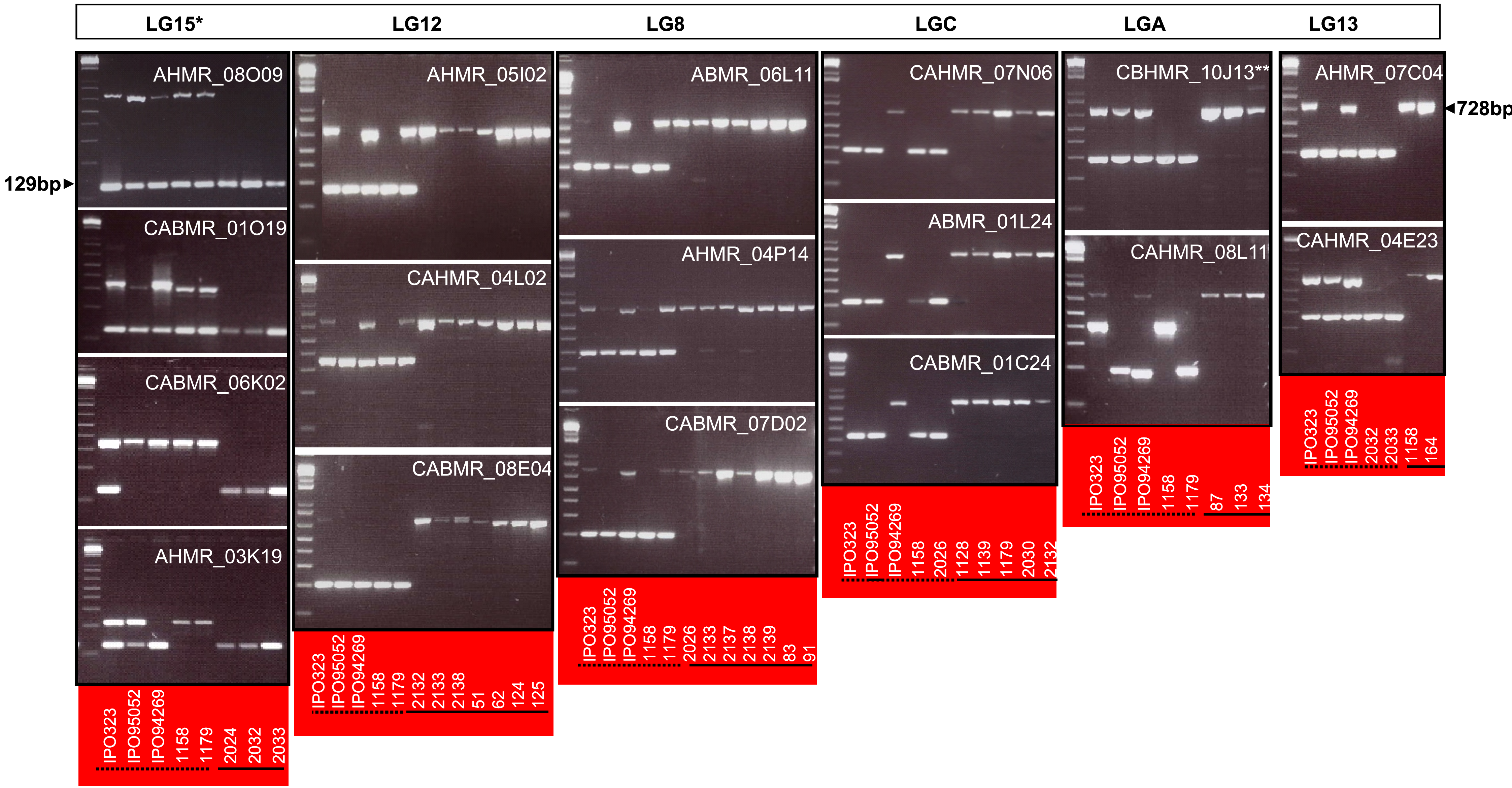
LG15*
LG12
LG8
LGC
LGA
LG13
129bp
728bp
AHMR_08O09
CABMR_01O19
CABMR_06K02
AHMR_03K19
AHMR_05I02
CAHMR_04L02
CABMR_08E04
ABMR_06L11
AHMR_04P14
CABMR_07D02
CAHMR_07N06
ABMR_01L24
CABMR_01C24
CBHMR_10J13**
CAHMR_08L11
AHMR_07C04
CAHMR_04E23
IPO323 IPO95052 IPO94269 1158 1179 2024 2032 2033
IPO323 IPO95052 IPO94269 1158 1179 2132 2133 2138 51 62 124 125
IPO323 IPO95052 IPO94269 1158 1179 2026 2133 2137 2138 2139 83 91
IPO323 IPO95052 IPO94269 1158 2026 1128 1139 1179 2030 2132
IPO323 IPO95052 IPO94269 1158 1179 87 133 134
IPO323 IPO95052 IPO94269 2032 2033 1158 164

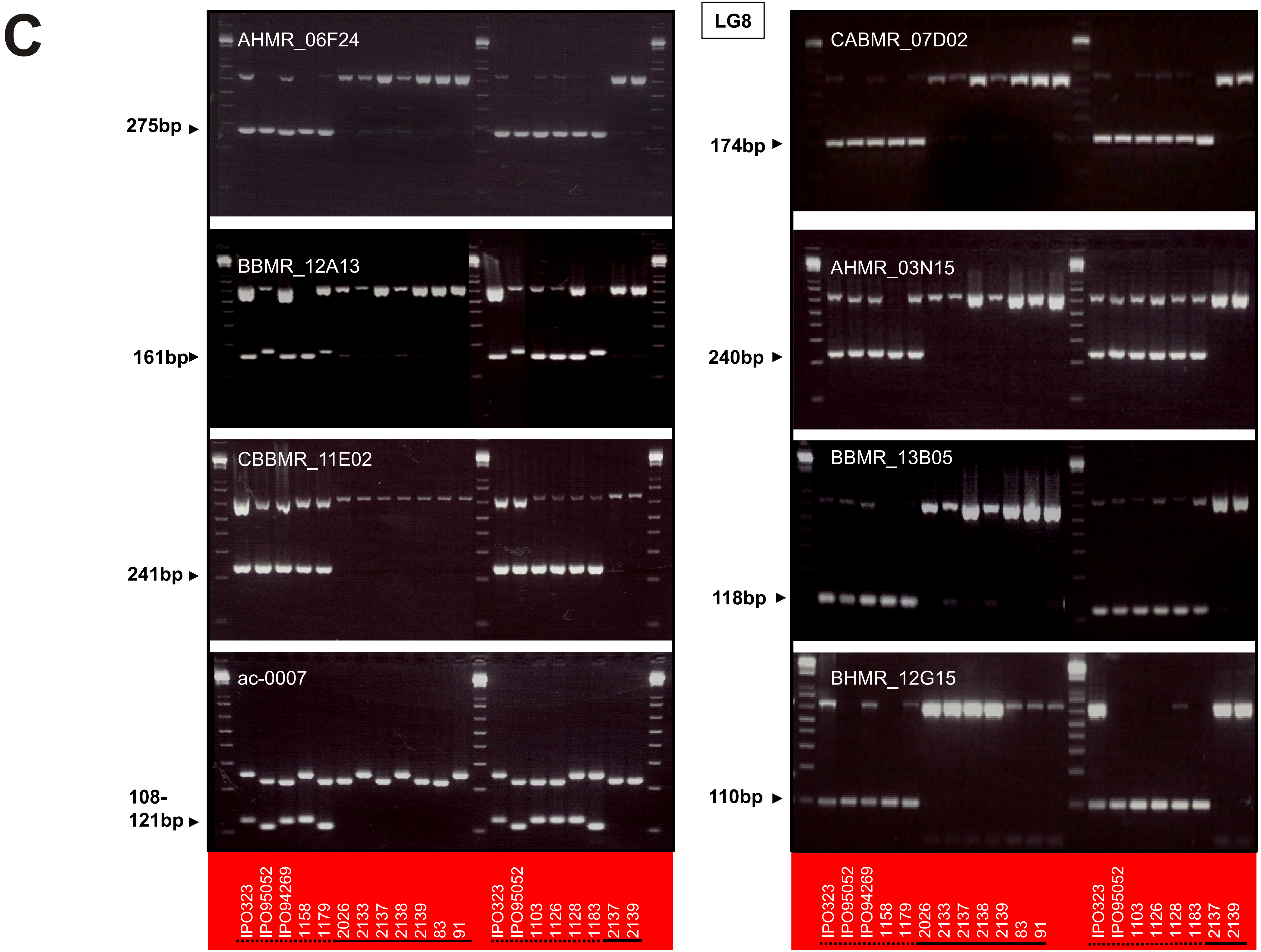
C
LG8
AHMR_06F24
275bp
BBMR_12A13
161bp
CBBMR_11E02
241bp
ac-0007
108-
121bp
CABMR_07D02
174bp
AHMR_03N15
240bp
BBMR_13B05
118bp
BHMR_12G15
110bp
IPO323 IPO95052 IPO94269 1158 1179 2026 2133 2137 2138 2139 83 91
IPO323 IPO95052 1103 1126 1128 1183 2137 2139
IPO323 IPO95052 IPO94269 1158 1179 2026 2133 2137 2138 2139 83 91
IPO323 IPO95052 1103 1126 1128 1183 2137 2139
