# Supplementary material for: Meiosis Drives Extraordinary Genome Plasticity in the Haploid Fungal Plant Pathogen Mycosphaerella graminicola
Source: PLoS One. 2009 Jun 10;4(6):e5863. doi: 10.1371/journal.pone.0005863 (PMC2689623; doi:10.1371/journal.pone.0005863)
Supplement: Table S1 — Mycosphaerella graminicola progeny isolates (n = 76) from the IPO323×IPO94269 in planta cross, that was made on the susceptible bread wheat cultivar Obelisk, that were used for hybridization to the DArT arrays. (0.05 MB DOC) [file pone.0005863.s005.doc]

**Table S1.** *Mycosphaerella graminicola* progeny isolates (n=76) from the IPO323 x IPO94269 *in planta* cross, that was made on the susceptible bread wheat cultivar Obelisk, that were used for hybridization to the DArT arrays.

| Isolate number | Used for construction of genetic linkage map | Isolate number | Used for construction of genetic linkage map |
| --- | --- | --- | --- |
| IPO323^1^ | - | 116 | - |
| IPO94269^2^ | - | 117 | Yes |
| 1 | Yes | 118 | Yes |
| 10 | Yes | 119 | Yes |
| 11 | - | 124 | Yes |
| 12 | Yes | 125 | Yes |
| 14 | Yes | 126 | Yes |
| 16 | - | 131 | Yes |
| 18 | - | 132 | Yes |
| 22 | - | 134 | Yes |
| 23 | Yes | 136 | Yes |
| 24 | Yes | 137 | Yes |
| 25 | - | 139 | - |
| 27 | Yes | 140 | - |
| 29 | Yes | 142 | Yes |
| 30 | - | 144 | Yes |
| 36 | - | 147 | Yes |
| 40 | Yes | 148 | - |
| 46 | - | 150 | - |
| 47 | Yes | 157 | Yes |
| 50 | Yes | 158 | Yes |
| 51 | Yes | 160 | Yes |
| 58 | - | 164 | Yes |
| 62 | Yes | 167 | Yes |
| 68 | Yes | 173 | Yes |
| 70 | - | 174 | Yes |
| 73 | Yes | 176 | Yes |
| 83 | Yes | 179 | Yes |
| 84 | Yes | 180 | Yes |
| 87 | Yes | 182 | Yes |
| 88 | Yes | 183 | Yes |
| 90 | Yes | 184 | Yes |
| 91 | Yes | 192 | Yes |
| 94 | Yes | 193 | Yes |
| 95 | Yes | 197 | Yes |
| 100 | Yes | 198 | Yes |
| 109 | Yes | 200 | Yes |
| 110 | - | 202 | Yes |
| 111 | Yes |  |  |
| 115 | Yes |  |  |

^1^ Parental isolate IPO323 was isolated from the bread wheat cultivar Arminda.

^2^ Parental isolate IPO94269 was isolated from the bread wheat cultivar Vivant.
