# Supplementary material for: Meiosis Drives Extraordinary Genome Plasticity in the Haploid Fungal Plant Pathogen Mycosphaerella graminicola
Source: PLoS One. 2009 Jun 10;4(6):e5863. doi: 10.1371/journal.pone.0005863 (PMC2689623; doi:10.1371/journal.pone.0005863)
Supplement: Table S2 — Mycosphaerella graminicola progeny isolates (n = 164) from the IPO323×IPO95052 in planta crosses that were made on the bread wheat cultivar Obelisk and the durum wheat cultivar Inbar. Sixteen isolates (gray-shaded) were not used, leaving a total of 148 that were used in the construction of the genetic linkage map. The first two numbers indicate the year of isolation and the next three numbers the order of isolation. (0.08 MB DOC) [file pone.0005863.s006.doc]

**Table S2.** Mycosphaerella graminicola progeny isolates (n=164) from the IPO323 x IPO95052 in planta crosses that were made on the bread wheat cultivar Obelisk and the durum wheat cultivar Inbar. Sixteen isolates (gray-shaded) were not used, leaving a total of 148 that were used in the construction of the genetic linkage map. The first two numbers indicate the year of isolation and the next three numbers the order of isolation.

| Isolated from bread wheat cultivar Obelisk | | | | | | Isolated from durum wheat cultivar Inbar | | | |
| --- | --- | --- | --- | --- | --- | --- | --- | --- | --- |
| IPO323^1^ | | 01 | 135 | 01 | 171 | IPO95052^2^ | | 02 | 043 |
| 01 | 101 | 01 | 136 | 01 | 172 | 01 | 426 | 02 | 044 |
| 01 | 102 | 01 | 137 | 01 | 173 | 01 | 427 | 02 | 045 |
| 01 | 103 | 01 | 138 | 01 | 174 | 01 | 428 | 02 | 046 |
| 01 | 104 | 01 | 139 | 01 | 175 | 01 | 429 | 02 | 047 |
| 01 | 105 | 01 | 140 | 01 | 176 | 01 | 430 | 02 | 121 |
| 01 | 106 | 01 | 141 | 01 | 177 | 01 | 431 | 02 | 122 |
| 01 | 107 | 01 | 142 | 01 | 178 | 01 | 432 | 02 | 123 |
| 01 | 108 | 01 | 143 | 01 | 179 | 01 | 433 | 02 | 124 |
| 01 | 109 | 01 | 144 | 01 | 180 | 01 | 434 | 02 | 125 |
| 01 | 110 | 01 | 146 | 01 | 181 | 01 | 435 | 02 | 126 |
| 01 | 111 | 01 | 147 | 01 | 182 | 01 | 436 | 02 | 127 |
| 01 | 112 | 01 | 148 | 01 | 183 | 01 | 437 | 02 | 128 |
| 01 | 113 | 01 | 149 | 01 | 184 | 01 | 438 | 02 | 129 |
| 01 | 114 | 01 | 150 | 01 | 185 | 01 | 439 | 02 | 130 |
| 01 | 115 | 01 | 151 | 01 | 186 | 01 | 440 | 02 | 131 |
| 01 | 116 | 01 | 152 | 01 | 187 | 02 | 024 | 02 | 132 |
| 01 | 117 | 01 | 153 | 01 | 188 | 02 | 025 | 02 | 133 |
| 01 | 118 | 01 | 154 | 01 | 189 | 02 | 026 | 02 | 134 |
| 01 | 119 | 01 | 155 | 01 | 190 | 02 | 027 | 02 | 135 |
| 01 | 120 | 01 | 156 | 01 | 191 | 02 | 028 | 02 | 136 |
| 01 | 121 | 01 | 157 | 01 | 192 | 02 | 029 | 02 | 137 |
| 01 | 122 | 01 | 158 | 01 | 193 | 02 | 030 | 02 | 138 |
| 01 | 123 | 01 | 159 | 01 | 194 | 02 | 031 | 02 | 139 |
| 01 | 124 | 01 | 160 | 01 | 195 | 02 | 032 | 02 | 140 |
| 01 | 125 | 01 | 161 | 01 | 196 | 02 | 033 | 02 | 141 |
| 01 | 126 | 01 | 162 | 01 | 197 | 02 | 034 |  |  |
| 01 | 127 | 01 | 163 | 01 | 198 | 02 | 035 |  |  |
| 01 | 128 | 01 | 164 | 01 | 199 | 02 | 036 |  |  |
| 01 | 129 | 01 | 165 | 01 | 200 | 02 | 037 |  |  |
| 01 | 130 | 01 | 166 | 01 | 421 | 02 | 038 |  |  |
| 01 | 131 | 01 | 167 | 01 | 422 | 02 | 039 |  |  |
| 01 | 132 | 01 | 168 | 01 | 423 | 02 | 040 |  |  |
| 01 | 133 | 01 | 169 | 01 | 424 | 02 | 041 |  |  |
| 01 | 134 | 01 | 170 | 01 | 425 | 02 | 042 |  |  |

^1^ Parental isolate IPO323 was isolated from the bread wheat cultivar Arminda

^2^ Parental isolate IPO95052 was isolated from an unknown durum wheat cultivar.
