# Supplementary material for: Meiosis Drives Extraordinary Genome Plasticity in the Haploid Fungal Plant Pathogen Mycosphaerella graminicola
Source: PLoS One. 2009 Jun 10;4(6):e5863. doi: 10.1371/journal.pone.0005863 (PMC2689623; doi:10.1371/journal.pone.0005863)
Supplement: Table S3 — The adapter and primer oligonucleotide sequences used for generation of the genomic representation (cloning) from Mycosphaerella graminicola isolates IPO323 and IPO95052 and for hybridization to the micro-arrays (genotyping) of parental and progeny isolates. (0.04 MB DOC) [file pone.0005863.s007.doc]

**Table S3.** The adapter and primer oligonucleotide sequences used for generation of the genomic representation (cloning) from *Mycosphaerella graminicola* isolates IPO323 and IPO95052 and for hybridization to the micro-arrays (genotyping) of parental and progeny isolates.

| **Endonuclease and recognition site** | **Used for** | **Adapter sequences^a^** | **Primer sequences (5’ to 3’)** |  |
| --- | --- | --- | --- | --- |
| *Hind*III  5’-A^↓^AGCTT-3’  3’-TTCGA_↑_A-5’ | Cloning | 5’-CTCGTAGACTGCGTCAC-3’  3’-ATCTGACGCAGTGTCGA -5’ | TAGACTGCGTCACAGCTT |  |
|  |  |  |  |  |
|  | Genotyping | 5’-GTGCTACAGTCGCTGAG-3’  3’-ATGTCAGCGACTCTCGA-5’ | TACAGTCGCTGAGAGCTT | |
|  |  |  |  |  |
| *Bam*HI  5’-G^↓^GATCC-3’  3’-CCTAG_↑_G-5’ | Cloning | 5’-CTCGTAGACTGCGATCA-3’  3’-CATCTGACGCTAGTCTAG-5’ | GTAGACTGCGATCAGATCC |  |
|  |  |  |  |  |
|  | Genotyping | 5’-GTGCTACAGTCGCTAGA-3’  3’-GATGTCAGCGATCTCTAG-5’ | CTACAGTCGCTAGAGATCC | |
|  |  |  |  |  |
| *Mse*I  5’-T^↓^TAA-3’  3’-AAT_↑_T-5’ | Cloning | 5’-ACTCGATCCTCACACGTA  AAGTATAGATCCCA-3’  3’- NH_2_-TTCATATCTAGGGTAT-5’ | ACTCGATCCTCACACGTA |  |
|  |  |  |  |  |
|  | Genotyping | 5’-AGTGCATGGTGAGAGCTA  AACTATACATGGGA-3’  3’- NH_2_-TTGATATGTACCCTAT-5’ | AGTGCATGGTGAGAGCTA | |
|  |  |  |  |  |
| *Rsa*I  5’-GT^↓^AC-3’  3’-CA_↑_TG-5’ | Co-digestion | - | - |  |

^a^ Adapter sequences were formed by annealing the strands whose sequences are listed. Complementary sequences are underlined.
