# Supplementary material for: Meiosis Drives Extraordinary Genome Plasticity in the Haploid Fungal Plant Pathogen Mycosphaerella graminicola
Source: PLoS One. 2009 Jun 10;4(6):e5863. doi: 10.1371/journal.pone.0005863 (PMC2689623; doi:10.1371/journal.pone.0005863)
Supplement: Table S4 — Overview of Mycosphaerella graminicola F1 isolates that lack one or more linkage groups compared to the parental isolates IPO323, IPO94269 and IPO95052. (0.03 MB DOC) [file pone.0005863.s008.doc]

**Table S4.** Overview of *Mycosphaerella graminicola* F_1_ isolates that lack one or more linkage groups compared to the parental isolates IPO323, IPO94269 and IPO95052.

| Missing linkage group | Isolates in cross IPO323 x IPO95052 | Isolates in cross IPO323 x IPO94269^a^ |
| --- | --- | --- |
| 8 | 2026, 2133, 2138, 2137, 2139^b^ | 83, 91 |
| 12 | 2132, 2133, 2138 | 51, 62, 124, 125 |
| 13 | 1158 | 164 |
| 15 | 2024, 2032, 2033 | - |
| 21 | 1114, 1121, 1122, 1127, 1151, 1159, 1170, 1176, 1186, 1200, 2133, 2138, 2137, 2139 | c |
| A | - | 87, 134 and 133 |
| B | 1108, 1169, 1179, 1425, 1438, 2134, 2141 | c |
| C | 1128, 1139, 1179, 2030, 2132 | c |

^a^ In the IPO323 x IPO94269 progeny, isolate #40 is disomic for LG 13 and isolate #51 is disomic for LG 1.

^b^ Underlined isolates are identified twins

^c^ Not assessed, as the LGs only contained IPO323-derived markers.
