# Supplementary material for: Meiosis Drives Extraordinary Genome Plasticity in the Haploid Fungal Plant Pathogen Mycosphaerella graminicola
Source: PLoS One. 2009 Jun 10;4(6):e5863. doi: 10.1371/journal.pone.0005863 (PMC2689623; doi:10.1371/journal.pone.0005863)
Supplement: Table S5 — Primer sequences used to verify the absence of several linkage groups in some progeny isolates of the two crosses. The primers were developed using the sequences of the DArT markers located on these linkage groups. (0.07 MB DOC) [file pone.0005863.s009.doc]

**Table S5.** Primer sequences used to verify the absence of several linkage groups in some progeny isolates of the two crosses. The primers were developed using the sequences of the DArT markers located on these linkage groups.

| Linkage  group | DArT markers | Forward primer (5’-3’) | Reverse primer (5’-3’) |
| --- | --- | --- | --- |
| **LG 8** |  |  |  |
|  | AHMR_04P14 | GCTTAAGTGACTACGAGTGC | CGCAAAGCTTCGATCCATTC |
|  | CABMR_07D02 | GATATTGCTTGGTGGATCCG | ATGTACCGTTCGGCAGAGTC |
|  | ABMR_06L11 | GGATCCCATGTTGTTCAGAG | CCTCGAACGAGTCGGTTTAA |
|  | AHMR_06F24 | GGTAGGGCGAATATTGCTGA | AGTGTCGAGCGGTATCGAGT |
|  | BBMR_12 A13 | ATTCGGATGAGCAGAGCAAG | CTCATCGCCATCATCACATC |
|  | CBBMR_11E02 | CTAGGCAAGGAGATCGAACG | GATGAGCGCCTTGTTTTCTC |
|  | AHMR_03N15 | TGAAGGGAAGTGGATTCTGG | AAGCTTCCAGGGGAAATTGT |
|  | BBMR_13B05 | TCCGAACCCTTCTTGCTCTA | CCAGATACTCCATCGGCATT |
|  | BHMR_12G15 | ACGACTAGACTTTCGCTTCTTG | TTAAGAGCTCGGAAATCGTG |
| **LG 12** |  |  |  |
|  | CABMR_08E04 | TTAAGGACATGGTCAAGCCA | TCTCATCTGTGTGAGGATCC |
|  | CAHMR_04L02 | CGGTTAATAGCTAGAGTCAA | GATCAAGACAGGAAGCTTCG |
|  | AHMR_05I02 | CGTCTACCACTATCCGAGAT | CAGAAAGCTTCGGTCCTGCT |
| **LG 13** |  |  |  |
|  | AHMR_07C04 | GACGCAGGCCAGTCATTTAT | GTTCCAGCTCGCAAAAGCTT |
|  | CAHMR_04E23 | CCGCCTTAATCAGACTATCG | CATTCACTGTGACGAAGCTT |
| **LG 15** |  |  |  |
|  | CABMR_01O19 | GTCACTATCCTCGCCGCATT | CCGGAATAAATGGAGGATCC |
|  | AHMR_08O09 | GTGTAGATTCGCGAGACTGG | GCTTCTTGGAAGCTTTGGTC |
|  | CABMR_06K02 | GGATCCACGAGTAAGCACAA | CGAGCATTAAAGCCTTCACG |
|  | AHMR_03K19 | TTAAACAACCCTCATCTGCC | CGCTTGAAGCTTCACATCAC |
| **LG A** |  |  |  |
|  | CBHMR_10J13** | GAGGCAGGAAGATCGTTAAA | ATGTAGCGGTACCAATCGAC |
|  | CAHMR_08L11 | GCATTTCCTTAGGTTGGACC | CTTGCCTGTGGACTTTCTAG |
| **LG B** |  |  |  |
|  | CAHMR_02A19 | AAGCTTAGCAGCAGAACCCT | CAGGTTGCGATAGGAGTACG |
|  | ABMR_04L19 | ATACAAGACGACGCTTGATG | GGTCTCCAAGGGACATATCT |
|  | ABMR_08E05 | GCAGTAACGACACCGATACA | TATAGAGCTAGCAGGACTGG |
| **LG C** |  |  |  |
|  | CABMR_01C24 | GTCCCTATGCAGAGGATCCT | TTCAACATTAAGGAGGGCGG |
|  | CAHMR_07N06 | CAGTTAAAACTCCATCTCGG | GTAGCTGTAACAAAGCTTGC |
|  | ABMR_01L24 | CTCACGGAACGGATCCAAAG | CATATCGATTCCAACCAGCG |
| **LG 21** | CAHMR_05C20 | GGTAGTGTTGTGCCTTCGTT | GTAGTGAAGCTTGCTGATGG |
|  | CABMR_07P03 | GAATCGGCGTGTGCGCTATC | TTCTCAAAATCCGAGGATCC |
|  | CAHMR_05E06 | CGAATATCGGATGTTAAAAG | AGAGAAGCTTCAAGATATCG |
|  | CHMR_09B06 | TGGTAGCATGGTCGATGGAA | AACGTACCGCATCGATAGAG |
| ***Controls*** |  |  |  |
| **LG 1** | CABMR_07D07 **^a^** | GGATCCGAAACGTCCGAAGA | ACATCCAGAGGAAAGAACGC |
| ***LG 15*** | AHMR_08O09 **^b^** | GTGTAGATTCGCGAGACTGG | GCTTCTTGGAAGCTTTGGTC |

^a^ Primer control used in duplex PCR for LG15.

^b^ Primer control used in duplex PCR for LGs 8, 12, 13, A, B, C and 21.
