# Supplementary material for: Meiosis Drives Extraordinary Genome Plasticity in the Haploid Fungal Plant Pathogen Mycosphaerella graminicola
Source: PLoS One. 2009 Jun 10;4(6):e5863. doi: 10.1371/journal.pone.0005863 (PMC2689623; doi:10.1371/journal.pone.0005863)
Supplement: Table S6 — Primer sequences used to verify the disomy for linkage group 1, isolate #51. The primers were developed around InDels obtained by comparison of BAC-end sequences from parental isolate IPO94269 with the genome sequence of isolate IPO323. (0.04 MB DOC) [file pone.0005863.s010.doc]

**Table S6.** Primer sequences used to verify the disomy for linkage group 1, isolate #51. The primers were developed around InDels obtained by comparison of BAC-end sequences from parental isolate IPO94269 with the genome sequence of isolate IPO323.

| BAC-end IPO94269 | Scaffold 4 position (bp)^*^ | InDel size (bp) | Forward primer (5’-3’) | Reverse primer (5’-3’) |
| --- | --- | --- | --- | --- |
| 05D17 | 366301 | 6 | tgcaggacatcgatcttcac | tatgctcaaatggggcaaag |
| 11O21 | 642878 | 9 | tccacctctctgggctgatt | catttcctgcttctggaggt |
| 06P08 | 1411546 | 9 | ccatccaccgcgtaactaat | atgctgctggccatgagga |
| 04L04 | 1599247 | 10 | gaatacacgggatccattcg | ggcaccgtcaaagcttacat |
| 07C16 | 1913009 | 7 | gacctgggaaatgagctgac | ctcagggacacatgttggtg |
| 04L20 | 2276380 | 7 | gcgaattgttgagaagtcca | tctcgaaggatcagcgacat |
| 13G02 | 2528033 | 7 | cttccttcgtctccttcgtg | acatgggaacagaccggata |
| 12N14 | 2604850 | 7 | tgttgaggagggtgagatga | atcatgactggggtttgtcg |

^*^ Derived from genome assembly IPO323 v 2.5.
