# Supplementary material for: Meiosis Drives Extraordinary Genome Plasticity in the Haploid Fungal Plant Pathogen Mycosphaerella graminicola
Source: PLoS One. 2009 Jun 10;4(6):e5863. doi: 10.1371/journal.pone.0005863 (PMC2689623; doi:10.1371/journal.pone.0005863)
Supplement: Table S7 — Overview of type and number of molecular markers that were scored in the progeny of the cross between Mycosphaerella graminicola isolates IPO323 and IPO94269 before and after grouping. (0.03 MB DOC) [file pone.0005863.s011.doc]

**Table S7.** Overview of type and number of molecular markers that were scored in the progeny of the cross between *Mycosphaerella graminicola* isolates IPO323 and IPO94269 before and after grouping.

| Marker type | Isolate | Complexity reduction method | Number of markers | Unique segregation patterns | Percentage of total no. of unique segregation patterns |
| --- | --- | --- | --- | --- | --- |
| AFLP | IPO323 | 1 | 151 | 93 | 19.66 |
| AFLP | IPO94269 | 1 | 120 | 72 | 15.22 |
| DArT | IPO323 | BMR | 375 | 156 | 32.98 |
|  | IPO95052^2^ | BMR | 183 | 33 | 6.98 |
| DArT | IPO323 | HMR | 383 | 80 | 16.91 |
|  | IPO95052 | HMR | 101 | 28 | 5.92 |
| SSR | - | - | 25+1^3^ | 11 | 2.33 |
| *Mat* and *Avr* | - | - | 2 | 0 | - |
| Sum |  |  | 1341 | 473 | 100% |

^1^AFLP markers were generated using 11 *Eco*RI-*Msp*I primer combinations

^2^ DArT fragment was derived from isolate IPO95052, but segregated in IPO323 x IPO94269

^3^One SSR marker segregated in a diploid fashion and therefore could be positioned on two locations.
