# Supplementary material for: Meiosis Drives Extraordinary Genome Plasticity in the Haploid Fungal Plant Pathogen Mycosphaerella graminicola
Source: PLoS One. 2009 Jun 10;4(6):e5863. doi: 10.1371/journal.pone.0005863 (PMC2689623; doi:10.1371/journal.pone.0005863)
Supplement: Table S8 — Overview of type and number of molecular markers that were scored in the progeny of the cross between Mycosphaerella graminicola isolates IPO323 and IPO95052 before and after grouping. (0.04 MB DOC) [file pone.0005863.s012.doc]

**Table S8.** Overview of type and number of molecular markers that were scored in the progeny of the cross between *Mycosphaerella graminicola* isolates IPO323 and IPO95052 before and after grouping.

| Marker type | Isolate | Complexity reduction method | Number of markers | Unique segregation patterns | Percentage of total no. of unique segregation patterns |
| --- | --- | --- | --- | --- | --- |
| DArT | IPO323 | BMR | 265 | 137 | 27.62 |
|  | IPO95052 | BMR | 258 | 113 | 22.78 |
| DArT | IPO323 | HMR | 296 | 121 | 24.40 |
|  | IPO95052 | HMR | 335 | 120 | 24.19 |
| SSR | - | - | 6 | 4 | 0.81 |
| *Mat* and *Avr* | - | - | 2 | 1 | 0.20 |
| Sum |  |  | 1162 | 496 | 100 % |
