# Supplementary material for: Meiosis Drives Extraordinary Genome Plasticity in the Haploid Fungal Plant Pathogen Mycosphaerella graminicola
Source: PLoS One. 2009 Jun 10;4(6):e5863. doi: 10.1371/journal.pone.0005863 (PMC2689623; doi:10.1371/journal.pone.0005863)
Supplement: Table S9 — Identified twin isolates in the two progenies derived from crosses between either Mycosphaerella graminicola isolates IPO323 and IPO94269 or IPO323 and IPO95052. (0.04 MB DOC) [file pone.0005863.s013.doc]

**Table S9.** Identified twin isolates in the two progenies derived from crosses between either *Mycosphaerella graminicola* isolates IPO323 and IPO94269 or IPO323 and IPO95052.

| IPO323 x IPO94269 | | IPO323 x IPO95052 | |
| --- | --- | --- | --- |
| 9 | 10 | 01101 | 01102 |
| 22 | 27 | 01103 | 01126 |
| 109 | 110 | 01105 | 01106 |
| 111 | 112 | 01109 | 01112 |
| 116 | 117 | 01115 | 01119 |
| 133 | 134 | 01132 | 01133 |
| 148 | 150 | 01142 | 01143 |
| 156 | 158 | 01154 | 01162 |
| 159 | 160 | 01177 | 01184 |
| 174 | 175 | 01196 | 01197 |
| 176 | 178 | 01426 | 01433 |
|  |  | 01429 | 01430 |
|  |  | 01434 | 01437 |
|  |  | 01435 | 01439 |
|  |  | 02034 | 02047 |
|  |  | 02035 | 02043 |
|  |  | 02037 | 02046 |
|  |  | 02041 | 02042 |
|  |  | 02128 | 02131 |
|  |  | 02130 | 02136 |
|  |  | 02133 | 02138 |
|  |  | 02137 | 02139 |
|  |  | 02134 | 02141 |
