# Supplementary material for: Meiosis Drives Extraordinary Genome Plasticity in the Haploid Fungal Plant Pathogen Mycosphaerella graminicola
Source: PLoS One. 2009 Jun 10;4(6):e5863. doi: 10.1371/journal.pone.0005863 (PMC2689623; doi:10.1371/journal.pone.0005863)
Supplement: Table S11 — Overview of the number of markers for both crosses. Mapping was performed using the software package JoinMap 3.0. (0.03 MB DOC) [file pone.0005863.s015.doc]

**Table S11. Overview of the number of markers for both crosses. Mapping was performed using the software package JoinMap 3.0.**

| Genetic map | Total number of markers selected | Unique segregation patterns | Segregation distortion removed  (P ≤ 0.01) | Grouped | Mapped excluding segregation distortion | Mapped including segregation distortion | Total number of markers positioned on map |
| --- | --- | --- | --- | --- | --- | --- | --- |
| IPO323 x IPO94269 | 1341 | 473  35.3 % | 444 | 443 | 441 | 451 | 1317  98.21 % |
| IPO323 x IPO95052 | 1162 | 496  42.7 % | 458 | 457 | 457 | 486 | 1144  98.45 % |
| Bridge | 389 | 263  67.6 % | 243 | 241 | 236 | 251 | 372^a^  95.63 % |

^a^ To construct the bridge map, eight markers that showed translocations were removed and three common markers were placed on the individual linkage maps but not on the bridge map. This resulted in a total of 2078 genetic markers, which are comprised of 1793 DArT markers, 258 AFLP markers, 25 SSR markers and the PCR markers for mating type (*Mat*) and avirulence (*Avr*).
