# Supplementary material for: Meiosis Drives Extraordinary Genome Plasticity in the Haploid Fungal Plant Pathogen Mycosphaerella graminicola
Source: PLoS One. 2009 Jun 10;4(6):e5863. doi: 10.1371/journal.pone.0005863 (PMC2689623; doi:10.1371/journal.pone.0005863)
Supplement: Table S13 — Alignment of the identified linkage groups in the Mycosphaerella graminicola IPO323×IPO94269 and IPO323×IPO95052 mapping populations with the identified chromosomes in the Mycosphaerella graminicola genome sequence. (0.05 MB DOC) [file pone.0005863.s017.doc]

**Table S13.** Alignment of the identified linkage groups in the *Mycosphaerella graminicola* IPO323 x IPO94269 and IPO323 x IPO95052 mapping populations with the identified chromosomes in the Mycosphaerella graminicola genome sequence.

|  |  | IPO323 x IPO94269 | IPO323 x IPO95052 |
| --- | --- | --- | --- |
| **LGs** | **Chromosomes** | **cM** | **cM** |
| 5+10 | 1 | 198.2 | 197.3 |
| 6 | 2 | 160.1 | 150.1 |
| 2 | 3 | 163.7 | 154.3 |
| 1 | 4 | 120.0 | 112.6 |
| 3+22 | 5 | 125.5 | 126.4 |
| 7 | 6 | 117.6 | 136.0 |
| 4+17 | 7 | 133.3 | 117.0 |
| 11+20 | 8 | 80.3 | 93.3 |
| 18+19 | 9 | 141.1 | 130.0 |
| 9 | 10 | 93.2 | 118.1 |
| 14 | 11 | 92.6 | 63.8 |
| 23 | 12 | 105.3 | 92.6 |
| 16 | 13 | 31.7 | 100.0 |
| B | 14 | 23.2 | 46.0 |
| 8 | 15 | 61.1 | 65.7 |
| 15 | 16 | 53.3 | 63.1 |
| 13 | 17 | 43.8 | 25.0 |
| 21 | 18 | 1.7 | 21.0 |
| A | 19 | 20.0 | 60.4 |
| C | 20 | 1.7 | 14.5 |
| 12 | 21 | 58.4 | 40.6 |
| E | - | 6.7 | 8.5 |
| F | - | 20.1 | 9.9 |
| D |  | 1.8 |  |
| Sum |  | 1854.1 | 1946.4 |
