# Supplementary material for: Meiosis Drives Extraordinary Genome Plasticity in the Haploid Fungal Plant Pathogen Mycosphaerella graminicola
Source: PLoS One. 2009 Jun 10;4(6):e5863. doi: 10.1371/journal.pone.0005863 (PMC2689623; doi:10.1371/journal.pone.0005863)
Supplement: Table S14 — DArT and SSR markers that showed translocations between two genetic linkage maps derived from crosses between either Mycosphaerella graminicola isolates IPO323 and IPO94269 or IPO95052. (0.03 MB DOC) [file pone.0005863.s018.doc]

**Table S14.** DArT and SSR markers that showed translocations between two genetic linkage maps derived from crosses between either *Mycosphaerella graminicola* isolates IPO323 and IPO94269 or IPO95052.

| Markers | Position in one parent | Position in other parent |
| --- | --- | --- |
| CHMR_09D04, CHMR10D17 | IPO323; LG 21 | IPO95052; LG 18+19 |
| CHMR_14L04, CHMR_13P21 | IPO323; LG 3+22 | IPO95052; LG 5+10 |
| CBBMR_15H01 | IPO94269; LG 1 | IPO95052; LG 3+22 |
| CBBMR_14G17, CBMR_14D07 | IPO94269; LG 6 (7cM) | IPO95052; LG 6 (79cM) |
| CBBMR_11N22 | IPO94269; LG 7 | IPO95052; LG 9 |
| ggc-0003A/B | IPO323; LG21 | IPO94269; LG 4+17 |
