# Supplementary material for: Meiosis Drives Extraordinary Genome Plasticity in the Haploid Fungal Plant Pathogen Mycosphaerella graminicola
Source: PLoS One. 2009 Jun 10;4(6):e5863. doi: 10.1371/journal.pone.0005863 (PMC2689623; doi:10.1371/journal.pone.0005863)
Supplement: Table S15 — Back crosses and intercrosses of M. graminicola IPO323×IPO94269 progeny isolates with isolates that either lost or gained specific chromosomes. (0.05 MB DOC) [file pone.0005863.s019.doc]

**Table S15.** Back crosses and intercrosses of *M. graminicola* IPO323 X IPO94269 progeny isolates with isolates that either lost or gained specific chromosomes.

| **Type of cross** | **Isolates** | **Chromosome number polymorphisms** | **LG** | **Scaffold^*^** | **Size (Mbp)** | **Success** |
| --- | --- | --- | --- | --- | --- | --- |
| **Back crosses** | 323*40 | P /disomic LG13 | 13 | 16 | 0.58 | Yes |
|  | 323*62 | P /-LG12 | 12 | 21 | 0.39 | Yes |
|  | 323*87 | P/-LGA | A | 17 | 0.55 | Yes |
|  | 323*124 | P/-LG12 | 12 | 21 | 0.39 | Yes |
|  | 94269*51 | P/-LG12+ disomic LG1 | 1 | 4 | 2.88 | Yes |
|  | 94269*83 | P/-LG8 | 8 | 14 | 0.64 | Yes |
|  | 94269*91 | P/-LG8 | 8 | 14 | 0.64 | Yes |
|  | 94269*125 | P/-LG12 | 12 | 21 | 0.39 | Yes |
|  | 94269*133 | P/-LGA | A | 17 | 0.55 | Yes |
|  | 94269*134 | P/-LGA | A | 17 | 0.55 | Yes |
|  | 94269*164 | P/-LG13 | 13 | 16 | 0.58 | Yes |
| **Inter crosses of F1 progeny isolates** | 2133x2137 | -LG8,12,21/-LG8,21 | 8, 12 and 21 | 14, 21 and 20 | 0.64, 0.39, 0.56 | Yes |
|  | 2133x2132 | -LG8,12,21/-LG12,C | 8, 12, 21 and C | 14, 21, 20 and 18 | 0.64, 0.39, 0.56, 0.47 | Yes |
|  | 2132x2024 | -LG21,C/-LG15 | 21, C and 15 | 20, 18 and 15 | 0.56, 0.47, 0.6 | Yes |
|  | 2133x1179 | -LG8,12,21/-LGB,C | 8, 12, 21, B and C | 14, 21, 20, 13 and 18 | 0.64, 0.39, 0.56, 0.77, 0.47 | Yes |
|  | 2133x1158 | -LG8,12,21/-LG13 | 8, 12, 21 and 13 | 14, 21, 20 and 16 | 0.64, 0.39, 0.56, 0.58 | Yes |
|  | 51x124 | -LG12 + disomic LG1/-LG12 | 12 and 1 | 21 and 4 | 0.39, 2.88 | No |
| **Control** | 323x94269 | P/-LG21, -LGC | 21 and C | - | - | Yes |

^*^ Derived from genome assembly IPO323 v 2.5.
